# The Lifetime Health and Economic Burden of Smokeless Tobacco use in Bangladesh, India, and Pakistan: Results From ASTRAMOD

**DOI:** 10.1093/ntr/ntae067

**Published:** 2024-05-08

**Authors:** Kathryn Coyle, Prashant Kumar Singh, Ravi Kaushik, Rumana Huque, Zohaib Khan, Ravi Mehrotra, Kamran Siddiqi, Subhash Pokhrel

**Affiliations:** Health Economics Research Group (HERG), Department of Health Sciences, Brunel University London, Uxbridge, UK; Division of Preventive Oncology and Population Health, ICMR-National Institute of Cancer Prevention and Research, Noida, India; Department of Physiology, Maulana Azad Medical College, New Delhi, India; ARK Foundation, Dhaka, Bangladesh; Office of Research, Innovation, and Commercialization, Khyber Medical University, Peshawar, Pakistan; Centre for Health Innovation and Policy (CHIP) Foundation, Noida, India; Division of Global Public Health, Brunel University London, Uxbridge, UK; Department of Health Sciences and Hull York Medical School, University of York, York, UK; Health Economics Research Group (HERG), Department of Health Sciences, Brunel University London, Uxbridge, UK

## Abstract

**Introduction:**

Under the current policy landscapes, the lifetime health and economic burden of smokeless tobacco (ST) products, consumed by over 297 million ST users in South Asia, is unknown. The aim of this study was to estimate the lifetime health effects and costs attributable to current and future ST use in Bangladesh, India, and Pakistan where the majority of ST users live.

**Aims and Methods:**

We developed a Markov-based state-transition model (ASTRAMOD) to predict the lifetime costs of treatment of four diseases (oral, pharyngeal, esophageal cancers, and stroke) and disability-adjusted life years (DALYs), attributable to the current and future use of ST under existing ST policy scenario. Country-specific Global Adult Tobacco Surveys, life tables, and meta-analyses of South Asian and South East Asian studies were used to populate the model. A probabilistic sensitivity analysis evaluated the uncertainty in model predictions.

**Results:**

If there were no change in the current ST policies, the lifetime ST-attributable treatment costs would be over US$19 billion in India, over US$1.5 billion in Bangladesh, and over US$3 billion in Pakistan. For all countries, the attributable costs are higher for younger cohorts with costs declining with increasing age for those over 50. The model predicted that a typical 15-year-old male adoloscent would gain 0.07–0.18 life years, avert 0.07–0.19 DALYs, and generate a cost-savings of US$7–21 on healthcare spending if ST policies were changed to eliminate ST use.

**Conclusions:**

Policy interventions aimed at decreasing the uptake of ST and increasing quitting success have the potential to substantially decrease the economic and health burden of ST.

**Implications:**

This study provides the most comprehensive estimates of the lifetime health and economic burden of ST by 5-year age and sex cohorts. This is also the first study that highlights the scale of health and economic burden of ST in Bangladesh, India, and Pakistan if there were no changes in the current ST policies. Policymakers and practitioners can use the reported data to justify their decisions to improve current ST policies and practices in their country. Researchers can use the ASTRAMOD methodology to estimate the impact of future ST policy changes.

## Introduction

South and Southeast Asia is home to a large majority (>85%) of the 356 million users of smokeless tobacco (ST) globally.^1^ ST is associated with an increased risk of oral cancers, and mortality related to cardiovascular and cerebrovascular diseases.^2^ Policymakers are, however, faced with decisions regarding where the most impactful investments can be made.^3^ Understanding the lifetime health and economic burden of ST in South Asia could not only quantify the impact of current and future ST use if *status quo* in ST policymaking remained, but also serve as a baseline against which the impact of any future change in ST policy could be objectively measured.

Studies on the burden of ST in South Asia are sparse. John et al. (2009) used a prevalence-based attributable risk approach to estimate the economic (direct medical and indirect morbidity but not premature mortality) costs of smoking and ST use in India.^4^ In 2017, John (2019) also estimated the costs of diseases and deaths attributable specifically to bidi smoking in India.^5^ Amarasinghe et al.^6^ used a prevalence-based cost-of-illness approach to estimate direct treatment costs (government and out-of-pocket) and indirect costs of lost earnings due to mortality and morbidity.^6^ Smoking attributable fractions were used to derive the direct cost of smoking and indirect morbidity and mortality costs in Pakistan.^7^ In Bangladesh, a population-attributable risk was estimated to capture the relative morbidity and mortality risks of tobacco use, and then it was used as a multiplier to estimate health expenditure.^8^ Most recently, John et al. (2021) updated the Indian estimates and added indirect mortality costs of premature deaths.^9^

Whilst direct comparison is difficult due to differences in methods, data sources, and included outcomes, all studies demonstrated significant economic burden of current ST use and disparity between men and women in ST-attributable costs. ST use in India for the year 2004 amounted to US$285 million in direct medical care costs with additional US$104 million indirectly (cost of caregivers and the value of work loss).^4^ This is 23% of the total economic cost attributable to all types of tobacco use in India and women contributed more to the cost of ST (31%) than to smoked tobacco (7%). The revised estimates for the year 2017–2018 showed that 26% of the total tobacco-attributable costs (US$27.5 billion) was due to ST alone.^5^ In Bangladesh, total direct and indirect costs attributable to both smoking and ST use rose from 135.8 billion BDT (US$1.6 billion) in 2004 to 305.6 BDT (US$3.6 billion) in 2018 (separate cost estimates for ST are not available).^8^ These estimates considered more (29.6%) Bangladeshi women using ST compared to men (21.5%). In Pakistan, smoking cost US$3.85 billion in 2019 of which indirect costs (morbidity and mortality) accounted for 70%, men had 77% and 35–64 age group had 86% of the total costs.^7^ Although no costs specific to ST use are available for Pakistan, wider tobacco control policies are being advocated on the back of the above study.

Most of the existing studies are thus estimates of ST burden for a single year, consistent with cost-of-illness studies generally. There has been no study, however, that examined the burden incorporating the projected future use, quitting, and relapsing behaviors that could occur in one’s lifetime in response to the current (and arguably and inadequate) ST control policies in a country. Therefore, in this study, we sought to answer the following question: *What would be the lifetime costs and health impacts of ST use if the ST policy status quo in Bangladesh, India, and Pakistan continued?* It was expected that quantifying the lifetime costs of ST use in this way would provide the country policymakers with one of the critical evidences that they require as a benchmark (or the baseline) against which they can measure the impact of changing current policies.

This study was conducted for three countries—Bangladesh, India, and Pakistan—that together carry 82% of ST-related global disease burden.^10^ This study was a part of ASTRA (Addressing ST use and building Research Capacity in South Asia), a Global Health Research Group, that was set up with the funding from UK’s National Institute for Health and Care Research (NIHR) to generate new knowledge around prevention of premature deaths, disabilities, and economic loss due to ST use in adults and adolescents.^11^

## Materials and Methods

### Study Objective

We developed a Markov-based state-transition model to allow estimation of the net burden of ST over the life course. This model facilitated the valuation of the disease costs and health impacts (disability and death) associated with ST use based on the current consumption rate for three South Asian countries, Bangladesh, India, and Pakistan.

### Population

The group of interest was the current adult population (aged 15 and above) as of the year 2020. (Supplementary Appendix A). This reflects the age at which persistent ST uptake generally begins. For each country, the population was stratified into 5-year cohorts (ie, from 15 to 19 years to 70 to 74 years) and by sex (male and female).^12^ Estimates of the burden are obtained for each age-sex cohort, with estimates for the total population obtained by weighting the results from each stratum.

### Comparator

To estimate the net burden of ST, analysis compared lifetime outcomes based on current usage of ST incorporating projected uptake, quitting, and relapse behaviors to a scenario in which no ST is consumed (the baseline).

### Outcomes

The disease burden of ST was assessed through disability-adjusted life years (DALYs), years of life lost, and the cost of treatment of ST-related illnesses. A healthcare perspective, including both costs borne by the healthcare system and by individuals was taken.^13^ Each cohort was modeled over their lifetime (to 89 years of age, due to data uncertainty in the estimates above this age) with a discount rate of 3% per annum for costs and outcomes.^14^

### Economic Model (ASTRAMOD)

**Figure 1.**
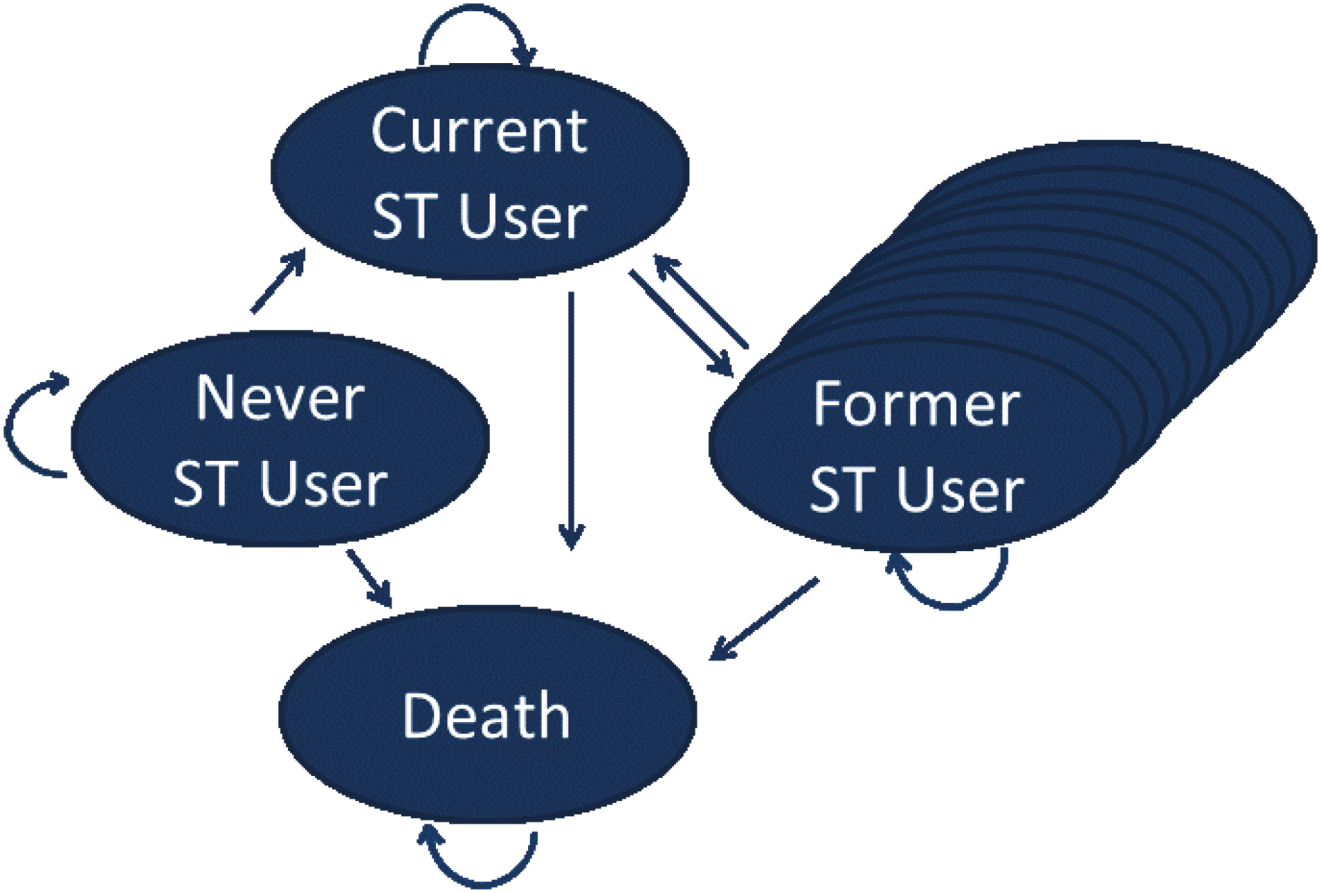
Model structure. *Each oval represents a primary state in the model. This is a prevalence-based model in which the states are related to smokeless tobacco (ST) use rather than diseases. The multiple ovals for former ST users represent tunnel states which reflect the time in years since quitting ST. The risk of relapse decreases with time since quitting as does the risk of ST related diseases and mortality. Four ST-attributable diseases (oral, oesophageal and pharyngeal cancer and stroke) for current and former ST users were included in the model. Disability weights derived for each of the four associated diseases from the 2016 Global Burden of Disease study were included in the model. Each oval represents a primary state in the model. This is a prevalence based model in which the states are related to smokeless tobacco (ST) use rather than diseases. The multiple ovals for former ST users represent tunnel states which reflect the time in years since quitting ST. The risk of relapse decreases with time since quitting as does the risk of ST related diseases and mortality. Four ST-attributable diseases (oral, oesophageal and pharyngeal cancer and stroke) for current and former ST users were included in the model. Disability weights derived for each of the four associated diseases from the 2016 Global Burden of Disease study were included in the model.

A series of Markov-state-transition models, of identical structure, were used to estimate long-term outcomes and costs for each cohort. Individuals were categorized as falling into one of four primary states within the model; never user, current user, former user, and dead (Figure 1). In this prevalence-based model, the states are related to ST use, rather than diseases. Patients could develop ST-related diseases with the total prevalence for the age/sex cycle equal to the reported prevalence for the specific country. Odds ratio for the risk of disease are used to apportion the prevalence between those who are current, former, and never ST users (Supplementary Appendix D for a detailed explanation of these calculations). With this approach, the diseases are not mutually exclusive. Recovery from disease could occur; however, this is not explicitly modeled, but rather reflected in the age/sex-specific prevalence adjusted for ST use.

The arrows in Figure 1 indicate how the members of the cohort can move from one state to another with each cycle of the model. The former ST user state has multiple ovals behind it to reflect the set of tunnel states that have been implemented for former ST users to allow for a reduction in the risk of disease and mortality with time since quitting ST and the probability of relapse which varies with time since quitting. The cycle length was 1 year which is consistent with data regarding quitting and relapse. Each cohort was distributed across the three ST user states based on the currently reported rates of ST use within the populations.^15–17^ The cohort was subjected to a set of transition probabilities within each cycle allowing them to either remain in their current state or move to one of the other model states, with death being an absorbing state. Mortality is modeled independent of disease; however, the same approach as was taken with ST diseases to adjust the age/sex/country-specific mortality by the relative risk of mortality for current and former versus never users of ST is employed.

### Transition Probabilities

Four sets of age- and sex-specific annual transition probabilities for each country were implemented within the model. These included: The probability of initiation of ST, the probability of making a successful quit attempt, long-term annual probabilities of relapse, and the probability of mortality specific to ST use status of an individual; that is, differential mortality was assumed for current, former, and never ST users. These probabilities were estimated using data from country-specific Global Adult Tobacco Survey’s and UN actuarial life tables.^15–20^ Prevalence was not kept the same based on the last survey data, rather, the past survey data has been used to estimate the transition probabilities between the four model states and it is these transition probabilities which are used to estimate the future prevalence of ST use (For methodological details and input data tables, Supplementary Appendix B, C, and D)

### Prevalence of ST-Related Diseases

Based on epidemiological data from the Global Burden of Disease study, the model estimated, for each cycle, the prevalence of ST-related diseases through application of the ST-related population attributable risk fractions relating to current and former ST users.^21^ The four diseases included in the model (oral, esophageal and pharyngeal cancer, and stroke) were selected based on both the underlying biological plausibility of the association with ST use and the proportion of the expected associated burden supported by the current evidence. They were selected based on a review of the published literature and consultation with experts.^20,22,23^ The population-attributable fraction of each of the co-morbid diseases for ST users was calculated using data regarding the disease prevalence from the Global Burden of Disease Study, the prevalence of users, former users, and never users from country-specific surveys and the relative effects (odds ratios) of ST on the prevalence of each disease. The same values for relative effects were used for all countries and were based on two recent meta-analyses of studies conducted in South East Asian and Indian populations.^20,22^ (For details, see Supplementary Appendix D).

### Costs

The costs within the model were those associated with the four diseases. Specific details regarding the costing estimates can be found in Supplementary Appendix E and F. Healthcare costs funded by both the healthcare system and out-of-pocket expenditures by patients were included. Costs were inflated to the year 2019 and converted to U.S. dollars to allow comparison between countries using World Bank Conversion rates.^24^ (Supplementary Appendix G).

### Utility Values

Disability weights for each of the four associated diseases were derived from the 2016 Global Burden of Disease study.^25^ As the disability weights are stage-specific for cancer and severity-specific for stroke, the average weight associated with a prevalent case of the disease was calculated by weighting by the time spent in each state for cancer and by the severity distribution of prevalent stroke. (Supplementary Appendix H).

### Key Assumptions

A number of assumptions were required for the analysis as is the case with all modeling studies. Key assumptions are listed here with additional rationale provided in Supplementary Appendix I. Relative risks of death in ST users sourced from the literature were used to adjust the population-based mortality rates and the risk was assumed to decrease with increased time since quitting ST. The same proportional decline in risk with time since quitting as was seen with smoked tobacco was applied to ST. Relative risks for former users who quit less than 5 years ago and who quit 5–10 years ago were estimated (Supplementary Appendix I for more details about the actual approach taken). The risk returned to that of never-users 10 years after quitting. The prevalence of each disease was assumed to be independent of the prevalence of other diseases and in the presence of multiple diseases, the disutility was assumed to be additive, given the lack of data to support otherwise. ST was assumed to not impact mortality or disease prevalence for people aged below 35 years. The prevalence of former ST users was assumed to remain at currently reported age- and sex-specific rates over the life course. Due to data limitations, the impact of ST was assessed through its association with the four diseases on which the meta-analyses reported the strongest evidence specific to the countries of interest.

### Handling Uncertainty

Probabilistic sensitivity analysis involving Monte Carlo simulation was implemented within the model to assess the effect of parameter uncertainty on the calculated outcomes using standard methods and conservative assumptions (Table 1). A set of 5000 estimates of outcomes and costs were drawn and implemented to estimate the 95% credible intervals for the estimates.

**Table 1.** Model Input Values

Parameter	Category	Value	Confidence interval	Distribution^^^	Reference
Relative risk of mortality vs. non-users					
	current users	1.25	1.08 to 1.44	Log normal (0.22, 0.07)	^20^
	former ST users up to 5 years	1.09	1.03 to 1.15	Log normal (0.08, 0.10)	^19,20^
	former ST users 5 to 10 years	1.02	1.01 to 1.04	Log normal (0.02, 0.10)	^19,20^
	former ST users 10 plus years	1.00		equal to non-users	^19,20^
Relative risk of disease vs. non-users					
Esophageal cancer	current users former users 0–4 years former users 5–10 years	3.17 1.77 1.21	2.77 to 3.64 1.42 to 2.11 0.98 to 1.45	Log normal (1.15, 0.07) Log normal (0.56, 0.10) Log normal (0.18, 0.10)	^22^
Oral cancer	current users former users 0–4 years former users 5–10 years	5.55 2.61 1.45	5.07 to 6.07 2.10 to 3.12 1.16 to 1.73	Lognormal (1.71, 0.05) Lognormal (0.95, 0.10) Lognormal (0.36, 0.10)	^22^
Pharyngeal cancer	current users former users 0–4 years former users 5–10 years	2.69 1.56 1.16	2.28 to 3.17 1.26 to 1.87 0.93 to 1.38	Lognormal (0.99, 0.08) Lognormal 0.44, 0.10) Lognormal (0.14, 0.10)	^22^
Stroke	current users former users 0–4 years former users 5–10 years	1.37 1.13 1.04	1.14 to 1.84 0.91 to 1.35 0.83 to 1.24	Lognormal (0.31, 0.12) Lognormal (0.12, 0.10) Lognormal (0.03, 0.10)	^20^
Proportion of prevalent strokes incident within 1 year		10.0%		Beta (5, 45)	^21^
Disutility weights					
Oral cancer	Males 15 to 49 years 50 to 69 years 70 years and above	0.164 0.172 0.196	0.062 to 0.266 0.068 to 0.276 0.087 to 0.305	Beta (8.20, 41.80) Beta (8.60, 41.40) Beta (9.80, 40.20)	^21,25,26^
Esophageal cancer	Females 15 to 49 years 50 to 69 years 70 years and above	0.143 0.165 0.191	0.047 to 0.239 0.063 to 0.267 0.083 to 0.299	Beta (7.15,42.85) Beta (8.25. 41.75) Beta (9.55, 40.45)	^21,25,26^
Pharyngeal cancer	Males 15 to 49 years 50 to 69 years 70 years and above	0.207 0.236 0.370	0.096 to 0.318 0.119 to 0.353 0.237 to 0.503	Beta (10.35, 39.65) Beta (11.80, 38.20) Beta (18.50, 31.50)	^21,25,26^
Stroke	Females 15 to 49 years 50 to 69 years 70 years and above	0.210 0.236 0.375	0.098 to 0.322 0.119 to 0.353 0.242 to 0.508	Beta (10.50, 39.50) Beta (11.80, 38.20) Beta (18.75, 31.25)	^25,27^
Disease costs (U.S. dollars)					
Cancer	India Pakistan	$2164	$1619 to $2408	Gamma (100, 1523.68)	^4,5,8,28^
	Bangladesh	$791	$602 to $896	Gamma (100, 667.90)	
Incident stroke	India Pakistan	$821	$614 to $913	Gamma (100, 578.04)	^4,5,8,28^
	Bangladesh	$601	$458 to $681	Gamma (100, 507.77)	
Prevalent stroke^*^	India Pakistan	$82	$39 to $114	Gamma (100, 57.80)	^4,5,8,28^
	Bangladesh	$60	$29 to $85	Gamma (100, 50.78)	

^*^estimated at 10% of the cost of incident stroke. See Supplementary Appendix F for further details.

^^^beta distributions are represented by alpha and beta, gamma distributions by scale and shape parameters and log normal distributions by the log of the mean and standard error.

## Results

**Figure 2.**
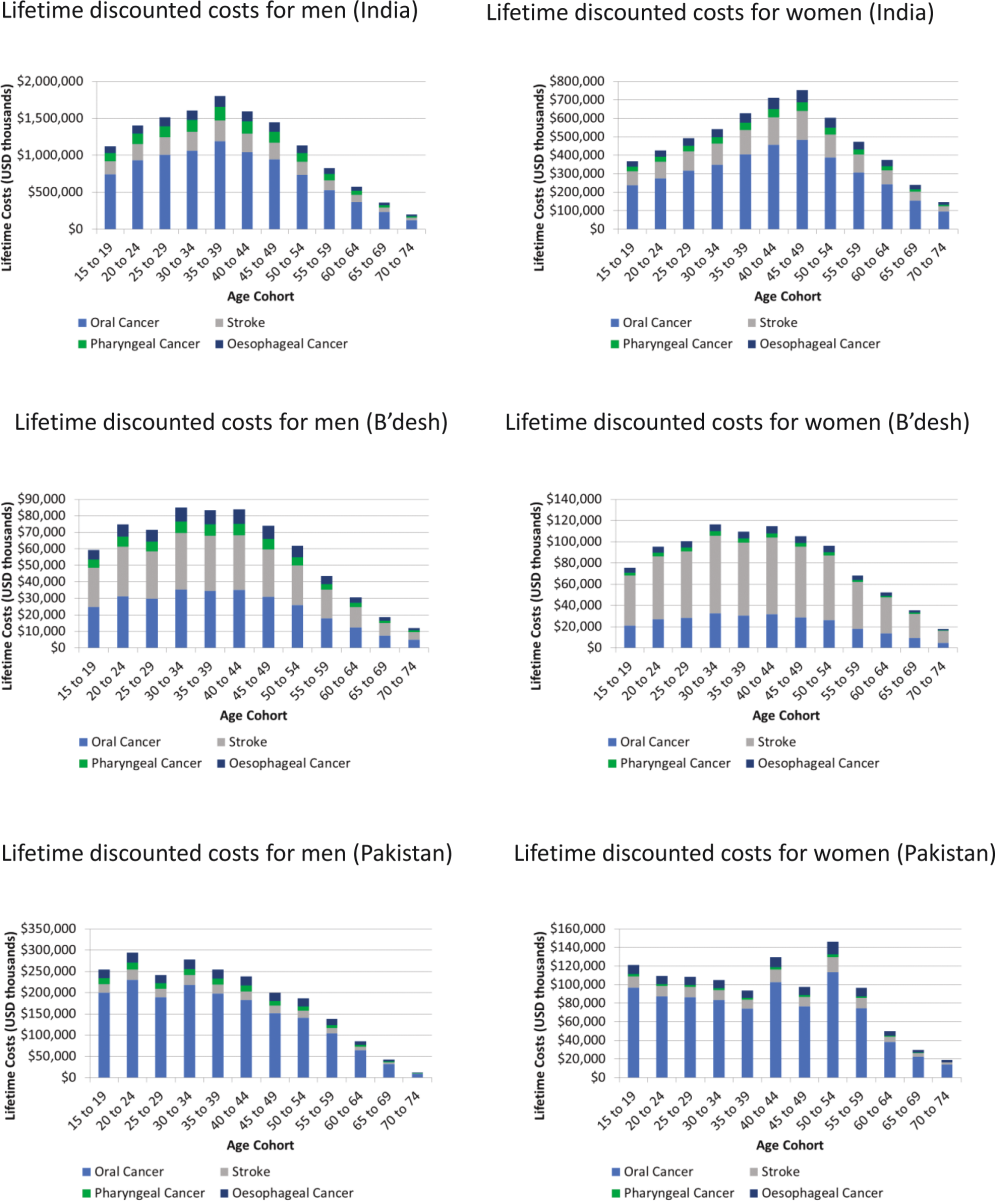
Lifetime discounted costs attributable to smokeless tobacco (US$, thousands).

The model predicted that the lifetime healthcare costs of ST use in Bangladesh, India, and Pakistan would be over US$1.5 billion, US$19 billion, and US$3.3 billion respectively. The total disease costs attributable to ST for each country by 5-year cohort for men are presented in Figure 2. For all countries, the attributable costs are higher for younger cohorts with costs declining with increasing age for those over 50. The greatest costs for men in India were in the 35 to 39-year-old cohort with total lifetime discounted costs of US$1.803 billion (95% credible interval: $1.148 to $2.064 billion). The greatest costs for men in Bangladesh were for the cohorts ranging from 30 to 44 years of age with costs ranging from US$83 million to US$85 million over the lifetime of each of the 5-year cohorts. The attributable costs for men in Pakistan peaked in the 20 to 24-year-old cohort and the 30 to 34-year-old cohort with costs of US$294 million and US$278 million, respectively.

On average, for a 15-year-old male in India, assuming the same trajectory of ST use over the life course as is currently evident, if ST use were eliminated, there would be an estimated gain of 0.18 life years and 0.19 DALYs averted per individual within this population. Additionally, savings of US$18.06 in healthcare spending per individual within the population could be generated. In Pakistan, the corresponding life year gains and DALYs averted would be 0.069 and 0.072 per individual, with healthcare savings of US$20.89. For Bangladesh, the corresponding life year gains and DALYs averted would be 0.18 and 0.19 per individual and healthcare savings of US$7.86.

In both India and Pakistan, oral cancer costs are the largest contributor to the overall burden of ST. In Bangladesh, however, attributable treatment costs for stroke are approximately equivalent to oral cancer for men and are more than double those of oral cancer for women (Figure 2).

The ST-attributable costs per male individual within the population are presented in Supplementary Appendix J. In all three countries the costs per individual are lower in younger age groups (15 to 29 years), increase to peak in middle age (30 to 59 years), and decline in older age groups (60 plus years), although not to the same extent as the total attributable costs. In comparing the trend in total and individual costs, it is evident that the significant population size at younger ages is contributing to the large overall burden in these cohorts.

The attributable costs follow a similar pattern for ST burden in women with the peak burden occurring in the middle-aged cohorts (30 to 59 years). As expected, due to lower usage of ST by women in all three countries, the costs for women are lower than those for men, although still substantial. Peak attributable costs occur in middle-age cohorts from 40 to 49 with total lifetime costs of approximately US$700 million per 5-year cohort for India. The costs in Bangladesh peak slightly earlier in the cohorts ranging from 30 to 44 years with total lifetime costs of between US$110 and US$117 million per 5-year cohort. The attributable costs in Pakistan for cohorts ranging from 15 to 59 years ranged from US$93 million to US$146 million per 5-year cohort. When comparing the total costs with the costs per individual, a similar pattern to that seen in men emerges with the ratio of costs in the younger cohorts (15 to 29 years) relative to the middle-aged cohorts (30 to 59 years) being larger due to lower relative costs in younger cohorts. This again points to the impact of the greater population in younger age ranges on the total costs.

When burden is measured in disability-adjusted life years (DALYs) lost, in India it increases from 11.6 million in the 15 to 19-year cohort, peaking in the 35 to 39-year male cohort at approximately 19 million DALYs lost and then declines in older cohorts (see Supplementary Appendix H, and for credibility intervals, Supplementary Appendix M). When examining the DALYs lost per individual, this decline in the older age brackets is not seen, suggesting that the overall burden decline is due to reduced population at older ages (60 plus years). The DALYs lost per individual are lower for younger cohorts but comparable for middle and older cohorts due to the impact of discounting. (Supplementary Appendix L). A similar pattern is seen in Bangladesh with the burden ranging from approximately 1.4 million DALYs lost in the 15- to 19-year male cohort to a peak of approximately 2.1 million DALYs lost in the 40 to 44 years cohort. The results for Pakistan are more variable across the age brackets ranging from 621 000 to 1 022 000 DALYs lost per 5-year male cohort in ages ranging from 15 to 59 and again declining in older ages.

For women in India, the pattern over the age cohorts is similar, but with the peak burden occurring slightly later in the 45 to 49 years cohort and the magnitude of the DALY burden is less than half that seen in men. In Bangladesh, there is a similar pattern of DALYs lost across the age cohorts in men and women; however, unlike in India, the DALYs lost is slightly higher in women as compared with men. The attributable DALYs lost in Pakistan for women is more variable across the age cohorts with peaks in the 40 to 44 and the 50 to 54 aged cohorts and, similar to India, a lower burden for women as compared with men.

**Figure 3.**
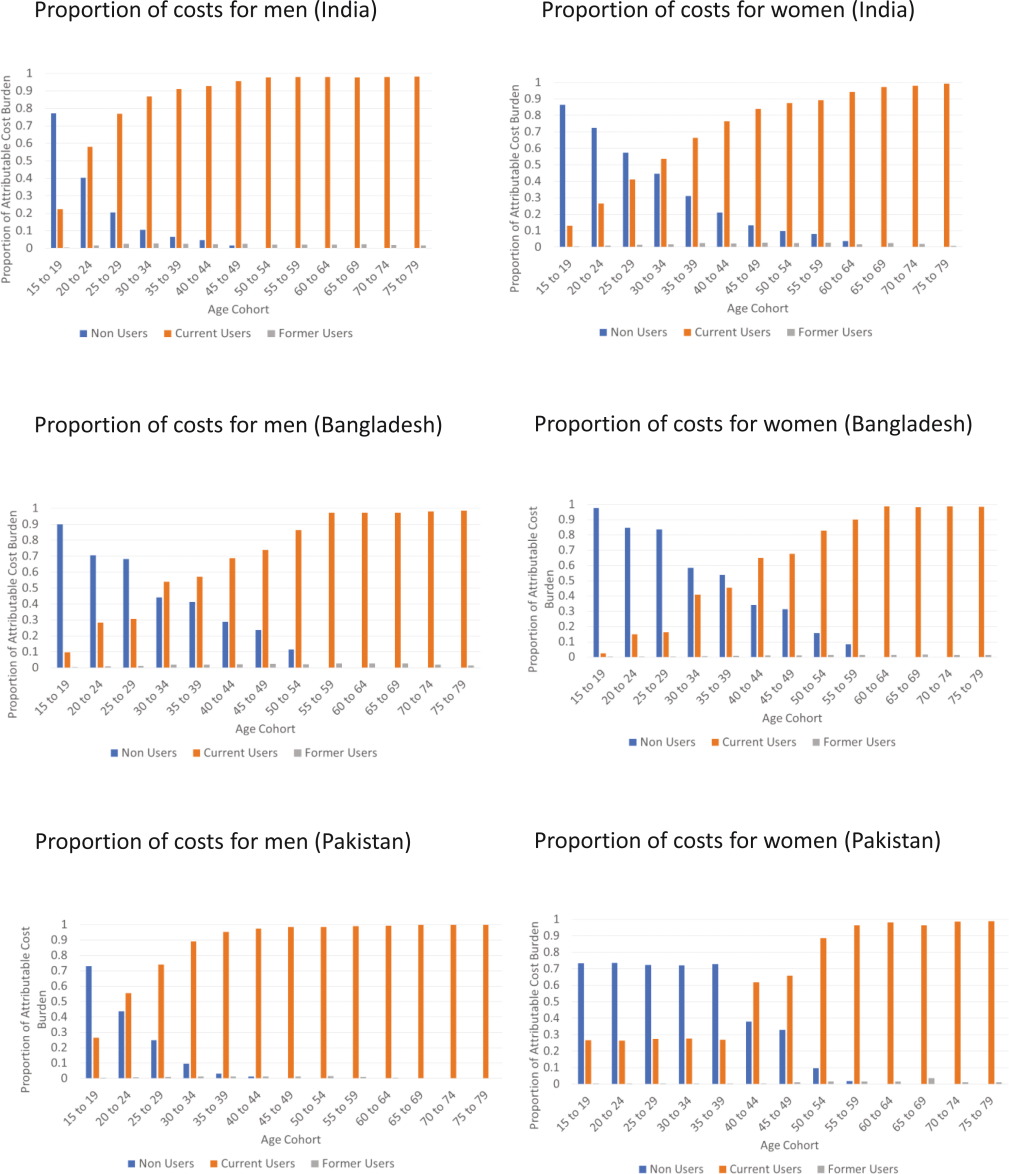
Proportion of costs attributable to non-users, current users and former users of smokeless tobacco.

The proportion of the overall cost burden that is attributable to those in each cohort who are currently non-users, current users, and former users are presented in Figure 3. For all countries, for both men and women in the younger age brackets the greater part of the burden is born by those who are not current users of ST, but go on to initiate ST use at some point in the future. In older age cohorts (55 years and older), the burden is borne primarily by current users. The shift in burden from non-users to current users occurs in the middle-age cohorts (30 to 59 years), although the point at which this occurs differs by sex and between countries. In general, women non-users bear the greater part of the burden further into middle age than men.

## Discussion

### New Knowledge and Policy Implications

This is the first study to comprehensively estimate the attributable burden of ST for South East Asian countries through modeling the impact on health, quality of life, and healthcare costs borne by 5-year age- and sex-specific cohorts over the life course. If the current usage patterns and policy *status quo* continue, ST is currently and will be even more so in the future, responsible for significant mortality and morbidity. The overall (absolute) burden is greatest for India due to the size of its ST users, with the highest burden borne by those in middle age, although per individual (relative) burden is comparable across the three countries. The burden is almost double for men compared with women in India and Pakistan; however, in Bangladesh, the burden is generally slightly greater for women compared with men.

With respect to age, current non-users in younger age ranges—who the model predicts will go on to take up ST in the future under the existing policy landscapes—account for a greater portion of the healthcare costs than current users. This is unlike in middle age where current users bear the largest share of the costs. The lower lifetime costs in the younger cohorts are due to the time delay in developing ST-related diseases (individuals must use ST for a period of time before they develop disease) and due to the impact of discounting on future costs and outcomes. Better understanding of the dynamics of the healthcare costs of ST within the populations thus allows for improved targeting of interventions. For example, in younger age cohorts, more may be gained through targeting non-users with interventions aimed at reducing uptake of ST whereas in middle age interventions targeted at cessation may be most efficient.

The current analysis has focused on estimation of the total healthcare costs associated with ST which has been shown to be substantial. Although the figures presented as healthcare costs are large, they are in fact conservative as cautious approaches were applied in selecting the best available estimates of relative risks and unit costs to model the outcomes. Nuances around the overall healthcare costs are important to consider, particularly in relation to how such a burden is being distributed to various age and sex groups. Clearly, interventions aimed at reducing uptake and increasing discontinuation of ST have the potential to positively impact health and reduce associated healthcare spending if they are shown to be effective and cost-effective.

### Comparison With the Literature

In an Indian study, the economic burden of ST was estimated at US$285 million for the year 2004 including only direct medical costs of treating tobacco-related disease.^4^ Indirect costs of ST, which include the cost of caregivers and value of work loss due to illness, amounted to US$104 million. A more recent study published in 2014 estimated the annual burden for 2011 of direct medical costs associated with ST in India at just over US$800 million.^29^ The attributable cost of all tobacco (smoked and smokeless) has also been estimated for Bangladesh at BDT 305.6 billion for 2018 (~US$3.6 billion) including both public and private healthcare expenditure, lost productivity costs for patients and caregivers and the costs associated with premature mortality.^30^ The burden for 2016 has also been estimated for the whole of the World Health Organizations Southeast Asian Region D as 5 366 257 DALYs lost, of which India accounts for 74% and Bangladesh 5%.^31^ Differences in the methodological approach taken for these studies versus the current study make a direct comparison of the results unrealistic. A key difference to note is that our model-based estimates are lifetime (as opposed to just the annual) burden of ST involving current and future use as well as relapsing behaviors counted together. In addition, our model offers a unique opportunity to provide the baseline against which countries can measure the impact of any ST policy changes in the future.

### Study Limitations

There are potential limitations to this study many of which are related to data availability. Economic modeling studies specific to India, Bangladesh, or Pakistan are limited within the literature, and we found no previous studies specific to ST. A contributing factor to this scarcity may be the challenge of locating appropriate data with which to populate the model. Specifically, the significant portion of costs borne by individuals in addition to challenges in attaining estimates of the government expenditure per case of disease meant that there is substantial uncertainty in the values used within the model. Lack of data regarding lost productivity and caregiver costs is likely to result in an underestimation of the true burden of ST. Although majority of input data were country-specific, some cost data (particularly for Pakistan) was not available and assumptions were needed to be made in such cases. This uncertainty may be reduced in future studies through either the conduct of costing studies or through increasing access to government costing data.

The burden of ST estimated within the current model is based only on four diseases for which considerable evidence is available to support the association with ST. Furthermore, one of the meta-analyses that provided model input data was based on South East Asian population. There are additional diseases (eg, cardiovascular disease and gastric cancer) for which there is evidence of an increased risk of ST; however, they were not included as the studies were not conducted on the relevant populations of this analysis or the quality of the evidence was not adequate.^22,32^ As such, the estimated cost is likely an underestimate of the true extent of the burden of ST.

Although from each country’s Global Adult Tobacco Surveys survey, it is evident that there are significant differences in the extent of use of different types of ST both between countries and between different regions within countries, the association between ST and disease was informed by values that were not specific to individual types of ST. There is evidence within the literature suggesting differential toxicities associated with different types of ST; however, the evidence was too limited to allow inclusion within the model.

A number of assumptions were required to estimate the risk of disease and mortality in those who had quit ST due to the lack of data on the reduction in risk with time since quitting. Should more data become available regarding how the risk changes over time, this would improve the accuracy of the predictions of the model.

## Conclusion

This study estimated lifetime healthcare costs and declined quality of life of ST use in Bangladesh, India, and Pakistan. The estimated substantial lifetime costs of ST use can be interpreted as the costs that would be accrued if there were no changes in the current ST policies. Positive changes in the current ST policies are needed to: (1) prevent people living in all three study countries from continuing to bear substantial burden of ST, both in terms of healthcare costs and declined quality of life; (2) avoid young people who are current non-users of ST to carry the most burden as they are predicted to take up ST at some point in their life; and (3) prevent women bearing the increasing burden of ST use.

## Supplementary material

Supplementary material is available at *Nicotine and Tobacco Research* online.

ntae067_suppl_Supplementary_Appendix

## Data Availability

The data that were used in the ASTRAMOD model are provided in various appendices under the Supplementary Materials. The corresponding author can be contacted for any future adaptation of ASTRAMOD.

## References

[1] Mehrotra R , Yadav A, Sinha DN, et al Smokeless tobacco control in 180 countries across the globe: call to action for full implementation of WHO FCTC measures. Lancet Oncol. 2019;20(4):e208–e217. 30942182 10.1016/S1470-2045(19)30084-1

[2] Siddiqi K , Husain S, Vidyasagaran A, et al Global burden of disease due to smokeless tobacco consumption in adults: an updated analysis of data from 127 countries. BMC Med. 2020;18(1):1–22. 32782007 10.1186/s12916-020-01677-9 PMC7422596

[3] John RM , Dauchy E, Goodchild M. Estimated impact of the GST on tobacco products in India. Tob Control. 2019;28(5):506–512. 30219796 10.1136/tobaccocontrol-2018-054479

[4] John RM , Sung HY, Max W. Economic cost of tobacco use in India, 2004. Tob Control. 2009;18(2):138–143. 19131453 10.1136/tc.2008.027466 PMC2655042

[5] John RM. Economic costs of diseases and deaths attributable to bidi smoking in India, 2017. Tob Control. 2019;28(5):513–518. 30337413 10.1136/tobaccocontrol-2018-054493

[6] Amarasinghe H , Ranaweera S, Ranasinghe T, et al Economic cost of tobacco-related cancers in Sri Lanka. Tob Control. 2018;27(5):542–546. 29079585 10.1136/tobaccocontrol-2017-053791 PMC6109234

[7] Nayab D , Nasir M, Memon JA, Siddique O. The Economic Cost of Tobacco-Induced Diseases in Pakistan. Islamabad: Pakistan Institute of Development Economics (PIDE); 2021.

[8] Faruque G , Ahmed M, Huq I, et al The Economic Cost of Tobacco Use in Bangladesh: A Health Cost Approach. Dhaka: Bangladesh Cancer Society; 2020.

[9] John RM , Sinha P, Munish VG, Tullu FT. Economic costs of diseases and deaths attributable to tobacco use in India, 2017–2018. Nicotine Tob Res. 2021;23(2):294–301. 32805055 10.1093/ntr/ntaa154

[10] Siddiqi K , Shah S, Abbas SM, et al Global burden of disease due to smokeless tobacco consumption in adults: analysis of data from 113 countries. BMC Med. 2015;13(1):194. 26278072 10.1186/s12916-015-0424-2 PMC4538761

[11] Readshaw A , Mehrotra R, Mishu M, et al Addressing smokeless tobacco use and building research capacity in South Asia (ASTRA). J Glob Health. 2020;10(1):10327. 10.7189/jogh.10.010327 PMC7101025 32257149

[12] United States Census Bureau. International Programs - Information Gateway - International Database. 2020. https://www.census.gov/data-tools/demo/idb/#/dashboard?COUNTRY_YEAR=2024&COUNTRY_YR_ANIM=2024. Accessed December 8, 2020.

[13] The World Bank Group. Out-of-pocket expenditure (% of current health expenditure), World Health Organization Global Health Expenditure database. 2020. https://data.worldbank.org/indicator/SH.XPD.OOPC.CH.ZS?view=chart. Accessed December 9, 2020.

[14] Claxton K , Revill P, Sculpher M, Wilkinson T, Cairns J, Briggs A. The Gates Reference Case: What It Is, Why It’s Important, and How to Use It. York: Centre for Health Economics; 2014.

[15] Tata Institute of Social Sciences, Ministry of Health and Family Welfare Government of India. Global Adult Tobacco Survey GATS 2 India 2016-2017. New Delhi: Ministry of Health and Family Welfare; 2017. https://ntcp.mohfw.gov.in/assets/document/surveys-reports-publications/Global-Adult-Tobacco-Survey-Second-Round-India-2016-2017.pdf

[16] World Health Organization Country Office for Bangladesh, Ministry of Health and Family Welfare. Global adult tobacco survey: bangladesh report 2009. Dhaka: World Health Organisation; 2009. https://extranet.who.int/ncdsmicrodata/index.php/catalog/259

[17] Pakistan Health Research Council, World Health Organization. Global adult tobacco survey: Pakistan 2014. Islamabad: World Health Organisation; 2016. https://extranet.who.int/ncdsmicrodata/index.php/catalog/257

[18] United Nations/ DESA/ Population Division. World population prospects 2019. 2019. https://population.un.org/wpp/Download/Standard/Mortality/. Accessed October 10, 2019.

[19] Cao Y , Kenfield S, Song Y, et al Cigarette smoking cessation and total and cause-specific mortality: a 22-year follow-up study among US male physicians. Arch Intern Med. 2011;171(21):1956–1959. 22123811 10.1001/archinternmed.2011.539 PMC3229033

[20] Sinha DN , Suliankatchi RA, Gupta PC, et al Global burden of all-cause and cause-specific mortality due to smokeless tobacco use: systematic review and meta-analysis. Tob Control. 2018;27(1):35–42. 27903956 10.1136/tobaccocontrol-2016-053302

[21] Institute for Health Metrics and Evaluation. Global health data exchange, global burden of disease results tool. 2020. http://ghdx.healthdata.org/gbd-results-tool. Accessed November 9, 2020.

[22] Sinha DN , Abdulkader RS, Gupta PC. Smokeless tobacco-associated cancers: a systematic review and meta-analysis of Indian studies. Int J Cancer. 2016;138(6):1368–1379. 26443187 10.1002/ijc.29884

[23] University of Oxford. Oxford centre for evidence-based medicine: levels of evidence. 2009. https://www.cebm.ox.ac.uk/resources/levels-of-evidence/oxford-centre-for-evidence-based-medicine-levels-of-evidence-march-2009. Accessed December 10, 2020.

[24] The World Bank Group. Official exchange rate (local currency unit per US$, period average) | Data. 2020. https://data.worldbank.org/indicator/PA.NUS.FCRF. Accessed December 10, 2020.

[25] . Institute for Health Metrics and Evaluation (IHME). Global Burden of Disease Study 2016 (GBD 2016) Disability Weights | GHDx. 2016. http://ghdx.healthdata.org/record/ihme-data/gbd-2016-disability-weights. Accessed May 26, 2020.

[26] Fitzmaurice C , Allen C, Barber RM, et al; Global Burden of Disease Cancer Collaboration. Global, regional, and national cancer incidence, mortality, years of life lost, years lived with disability, and disability-adjusted life-years for 32 cancer groups, 1990 to 2015: a systematic analysis for the global burden of disease study global burden. JAMA Oncol. 2017;3(4):524–548. 27918777 10.1001/jamaoncol.2016.5688 PMC6103527

[27] Pandian JD , Sudhan P. Stroke epidemiology and stroke care services in India. J Stroke. 2013;15(3):128–134. 24396806 10.5853/jos.2013.15.3.128 PMC3859004

[28] Ministry of Statistics and Programme Implementation. National Sample Survey (NSS). New Delhi: Ministry of Statistics and Programme Implementation; 2020.

[29] John R , Rout S, Kumar B, Arora M. Economic Burden of Tobacco Related Diseases in India. New Delhi: Ministry of Health and Family Welfare, Government of India; 2014.

[30] Nargis N , Faruque GM, Ahmed M, et al A comprehensive economic assessment of the health effects of tobacco use and implications for tobacco control in Bangladesh. Tob Control. 2021;31(6):723–729. 33653817 10.1136/tobaccocontrol-2020-056175

[31] Fitzmaurice C , Akinyemiju TF, Al Lami FH, et al; Global Burden of Disease Cancer Collaboration. Global, regional, and national cancer incidence, mortality, years of life lost, years lived with disability, and disability-adjusted life-years for 29 cancer groups, 1990 to 2016: a systematic analysis for the global burden of disease study. JAMA Oncol. 2018;4(11):1553–1568. 29860482 10.1001/jamaoncol.2018.2706 PMC6248091

[32] Ram Rohit TA. Smoking, smokeless tobacco consumption & coronary artery disease – a case control study. Natl J Community Med. 2012;3(2):264–268.

