# Supplementary material for: The Lifetime Health and Economic Burden of Smokeless Tobacco use in Bangladesh, India, and Pakistan: Results From ASTRAMOD
Source: Nicotine Tob Res. 2024 May 8;27(4):684–92. doi: 10.1093/ntr/ntae067 (PMC11931212; doi:10.1093/ntr/ntae067)
Supplement: ntae067_suppl_Supplementary_Appendix [file ntae067_suppl_supplementary_appendix.docx]

**Supplementary Materials, Appendices**

**Appendix A: Population Numbers**^1^

**Table A.1: Population by Age and Sex for India, Pakistan and Bangladesh in 2020.**

| **Sex** | **Age** | **Population** | | |
| --- | --- | --- | --- | --- |
|  |  | **India** | **Pakistan** | **Bangladesh** |
| Male | 15 to 19 | 62153754 | 12190318 | 7548975 |
| Male | 20 to 24 | 61269777 | 10928887 | 7637495 |
| Male | 25 to 29 | 57794800 | 9728501 | 6721030 |
| Male | 30 to 34 | 53489399 | 8515298 | 6084009 |
| Male | 35 to 39 | 49987699 | 7303756 | 5567743 |
| Male | 40 to 44 | 46115904 | 6214017 | 4921882 |
| Male | 45 to 49 | 41980723 | 5239727 | 4496155 |
| Male | 50 to 54 | 35907142 | 4588082 | 3903448 |
| Male | 55 to 59 | 29320623 | 3676910 | 3316781 |
| Male | 60 to 64 | 23124194 | 2849746 | 2625044 |
| Male | 65 to 69 | 17324501 | 1984934 | 2048125 |
| Male | 70 to 74 | 12027804 | 1295059 | 1468349 |
| Male | 75 to 79 | 7415100 | 866902 | 943729 |
| Male | 80 to 84 | 3676445 | 457569 | 502813 |
| Male | 85 to 89 | 1292368 | 160138 | 198018 |
| Male | 90 to 94 | 282732 | 33464 | 49562 |
| Male | 95 to 99 | 33550 | 3876 | 7113 |
| Male | 100 + | 1959 | 223 | 497 |
| Female | 15 to 19 | 54704937 | 11583293 | 7321305 |
| Female | 20 to 24 | 54034843 | 10369683 | 7680645 |
| Female | 25 to 29 | 51429767 | 9251314 | 7126810 |
| Female | 30 to 34 | 48939213 | 8120855 | 6668869 |
| Female | 35 to 39 | 46481566 | 6942042 | 6072248 |
| Female | 40 to 44 | 43539933 | 5877631 | 5475289 |
| Female | 45 to 49 | 40396572 | 4910379 | 5045754 |
| Female | 50 to 54 | 35055268 | 4339825 | 4146673 |
| Female | 55 to 59 | 29052650 | 3537181 | 3372606 |
| Female | 60 to 64 | 23394388 | 2886812 | 2743250 |
| Female | 65 to 69 | 18037720 | 2154337 | 2177827 |
| Female | 70 to 74 | 13112207 | 1513377 | 1614365 |
| Female | 75 to 79 | 8646670 | 1056402 | 1089388 |
| Female | 80 to 84 | 4727348 | 579674 | 619154 |
| Female | 85 to 89 | 1908421 | 214414 | 279065 |
| Female | 90 to 94 | 495294 | 46565 | 83634 |
| Female | 95 to 99 | 71278 | 5511 | 14747 |
| Female | 100 + | 5037 | 315 | 1231 |

# Appendix B: Details Regarding Transition Probabilities

The following were required for the economic model:

1. The distribution of the cohort upon entering the model with respect to their ST use status: current, former and never users and the time since quitting ST for former users
2. The annual uptake of ST
3. The annual probability of making a quit attempt and quit attempt success
4. The annual probability of relapse to reinitiate ST in years after quitting ST

**B.1 Distribution of Cohort Upon Entering Model**

A cohort of individuals enters the model at the start of the age range. The proportion of the cohort beginning in each of the model states (never, current and former ST users) is sourced from analysis of the GATs survey data.^2–4^ If we consider, for example, the model for 15 to 19 year old males for India, 96.13% reported not using ST at age 14, 3.83% reported using ST at age 14 and 0.04% reported being former users of ST. This is the assumed distribution of the cohort between the model states at the start of the model. An explanation of how these values were derived from the GATs data is provided below.

The percentage of individuals who initiated ST use before the age range of each of the respective models can be sourced from the GATS survey. The values can be found in Table B.1 below.

**Table B.1: Percentage of Total Population Who Initiated ST Before Age Range (includes all ever users)**^2–4^

| **Sex** | **Age Range** | **India** | **Pakistan** | **Bangladesh** |
| --- | --- | --- | --- | --- |
| Male | 15 to 19 | 0.0387 (beta: 38.59, 959.37) | 0.0379 (beta: 5.72, 145.17) | 0.0369 (beta: 6.74, 176.02) |
| Male | 20 to 24 | 0.2163 (beta: 216.74, 785.30) | 0.0834 (beta: 13.09, 143.85) | 0.1328 (beta: 34.55, 225.60) |
| Male | 25 to 29 | 0.3135 (beta: 417.93, 915.34) | 0.1059 (beta: 19.00, 160.49) | 0.1292 (beta: 36.24, 244.12) |
| Male | 30 to 34 | 0.3668 (beta: 591.34, 1020.64) | 0.1429 (beta: 32.39, 194.22) | 0.2931 (beta: 110.62, 266.78) |
| Male | 35 to 39 | 0.4121 (beta: 659.42, 940.91) | 0.1497 (beta: 34.08, 193.51) | 0.2897 (beta: 111.44, 273.21) |
| Male | 40 to 44 | 0.3744 (beta: 416.77, 696.30) | 0.1603 (beta: 33.03, 172.99) | 0.3708 (beta: 134.77, 228.71) |
| Male | 45 to 49 | 0.3724 (beta: 456.68, 769.59) | 0.1621 (beta: 32.91, 170.13) | 0.3695 (beta: 116.85, 199.37) |
| Male | 50 to 54 | 0.3560 (beta: 257.24, 465.35) | 0.1775 (beta: 12.34, 57.17) | 0.4136 (beta: 91.69, 130.02) |
| Male | 55 to 59 | 0.3464 (beta: 284.26, 536.36) | 0.1775 (beta: 12.34, 57.17) | 0.4655 (beta: 123.24, 141.49) |
| Male | 60 to 64 | 0.3589 (beta: 233.29, 416.72) | 0.1589 (beta: 5.94, 31.45) | 0.4951 (beta: 67.70, 69.05) |
| Male | 65 to 69 | 0.3758 (beta: 220.01, 365.43) | 0.1589 (beta: 5.94, 31.45) | 0.4951 (beta: 67.70, 69.05) |
| Male | 70 to 74 | 0.3949 (beta: 137.07, 210.03) | 0.1018 (beta: 5.05, 44.58) | 0.5863 (beta: 60.12, 42.42) |
| Male | 75 to 79 | 0.3459 (beta: 56.51, 106.87) | 0.1018 (beta: 5.05, 44.58) | 0.5863 (beta: 60.11, 42.42) |
| Male | 80 to 84 | 0.3767 (beta: 46.80, 77.43) | 0.1604 (beta: 2.76, 14.45) | 0.5145 (beta: 17.07, 16.10) |
| Male | 85 to 89 | 0.3780 (beta: 18.02, 29.65) | 0.1604 (beta: 2.76, 14.45) | 0.5145 (beta: 17.07, 16.10) |
| Female | 15 to 19 | 0.0144 (beta: 16.86, 1081.79) | 0.0153 (beta: 1.80, 115.36) | 0.0060 (beta: 1.59, 265.21) |
| Female | 20 to 24 | 0.0417 (beta: 75.27, 1731.27) | 0.0160 (beta: 5.15, 315.84) | 0.0630 (beta: 21.93, 326.24) |
| Female | 25 to 29 | 0.0700 (beta: 145.35, 1930.26) | 0.0144 (beta: 4.14, 284.10) | 0.0863 (beta: 62.87, 665.39) |
| Female | 30 to 34 | 0.0924 (beta: 162.58, 1597.45) | 0.0157 (beta: 6.06, 381.28) | 0.2082 (beta: 81.45, 309.79) |
| Female | 35 to 39 | 0.1281 (beta: 241.21, 1641.67) | 0.0099 (beta: 2.07, 207.90) | 0.2430 (beta: 128.94, 401.74) |
| Female | 40 to 44 | 0.1689 (beta: 175.95, 866.05) | 0.0474 (beta: 11.35, 228.17) | 0.4146 (beta: 123.47, 174.34) |
| Female | 45 to 49 | 0.2033 (beta: 253.10, 992.05) | 0.0454 (beta: 9.44, 198.56) | 0.4150 (beta: 118.57, 167.13) |
| Female | 50 to 54 | 0.2061 (beta: 187.57, 722.61) | 0.1125 (beta: 11.14, 87.86) | 0.5768 (beta: 128.71, 94.44) |
| Female | 55 to 59 | 0.2164 (beta: 181.74, 658.17) | 0.1191 (beta: 12.24, 90.53) | 0.5728 (beta: 100.71, 75.11) |
| Female | 60 to 64 | 0.2551 (beta: 175.70, 512.96) | 0.0927 (beta: 8.18, 80.08) | 0.6949 (beta: 109.29, 47.99) |
| Female | 65 to 69 | 0.2749 (beta: 109.91, 289.92) | 0.0927 (beta: 8.18, 80.08) | 0.6949 (beta: 109.29, 47.99) |
| Female | 70 to 74 | 0.3046 (beta: 105.45, 240.74) | 0.1272 (beta: 2.59, 17.77) | 0.5935 (beta: 34.92, 23.91) |
| Female | 75 to 79 | 0.2919 (beta: 44.75, 108.55) | 0.1272 (beta: 2.59, 17.77) | 0.5935 (beta: 34.92, 23.91) |
| Female | 80 to 84 | 0.3778 (beta: 37.38, 61.56) | 0.1905 (beta: 2.69, 11.45) | 0.6296 (beta: 24.66, 14.51) |
| Female | 85 to 89 | 0.3199 (beta: 14.01, 29.78) | 0.1905 (beta: 2.69, 11.45) | 0.6296 (beta: 24.66, 14.51) |

These values represent ever users of ST and include both those who initiated ST use and continue to use ST at the starting age range of the model and those who initiated ST but also quit ST use before the starting age range of the model (former users). Data from the GATs survey was used to specify the proportion that are current users as opposed to those that are former users upon entering the models. This distribution is provided in Table B.2 below.

To provide more stable estimates given limited data for Pakistan and Bangladesh, average values across age ranges were used. In cases where uncertainty values were not available for the probabilistic distributions, a conservative small sample size of 50 was assumed.

Note: The figures in the tables B.2-B.5 below only applies to the distribution of current and former smokers in the year prior to entering the model. Where there was small sample size within the GATs surveys for this question, the results may be prone to bias if the individual age brackets were used. In such cases, the age brackets were therefore combined in order to provide more stable estimates (hence the variable age ranges reported in the tables). Given that it only impacts the distribution of the population upon entering the model and not how the cohort transitions through the model, it will have minimal impact on the results.

**Table B.2: Distribution of Current and Former Users in Interval Before Start of Model**^2–4^

| **Sex** | **Age** | **Daily ST** | **Occasional ST** | **Former ST** |
| --- | --- | --- | --- | --- |
| **India** |  |  |  |  |
| Male | 15 to 19 | 95.580 | 3.452 | **0.971 (beta: 2.647, 270.025)** |
| Male | 20 to 24 | 94.170 | 3.796 | **2.035 (beta: 6.664, 320.717)** |
| Male | 25 to 29 | 93.860 | 3.006 | **3.100 (beta: 10.315, 322.453)** |
| Male | 30 to 34 | 92.190 | 4.504 | **3.302 (beta: 16.812, 492.394)** |
| Male | 35 to 39 | 94.120 | 3.368 | **2.515 (beta: 22.487, 871.5657)** |
| Male | 40 to 44 | 93.510 | 4.070 | **2.420 (beta: 20.953, 845.009)** |
| Male | 45 to 49 | 92.530 | 3.572 | **3.898 (beta: 16.344, 402.926)** |
| Male | 50 to 54 | 92.470 | 3.091 | **4.441 (beta: 17.078, 367.480)** |
| Male | 55 to 59 | 94.230 | 1.927 | **3.843 (beta: 9.842, 246.273)** |
| Male | 60 to 64 | 92.680 | 2.207 | **5.110 (beta: 16.500, 306.375)** |
| Male | 65 to 69 | 91.240 | 2.126 | **6.635 (beta: 9.224, 129.802)** |
| Male | 70 to 74 | 83.410 | 6.656 | **9.934 (beta: 11.208, 101.622)** |
| Male | 75 to 79 | 78.210 | 7.008 | **14.785 (beta: 12.345, 71.151)** |
| Male | 80 to 84 | 88.290 | 4.393 | **7.318 (beta: 3.889, 49.252)** |
| Female | 15 to 84 | 90.9304 | 3.77329 | **5.296 (beta: 62.355, 1114.97)** |
| **Pakistan** |  |  |  |  |
| Male | 15 to 24 | 90.3482 | 8.356885 | **1.2949 (beta: 0.647, 49.353)*** |
| Male | 25 to 44 | 93.53428 | 4.298575 | **2.1672 (beta: 1.084, 48.916)*** |
| Male | 45 to 64 | 93.63018 | 0.761743 | **5.6081 (beta: 2.804, 47.196)*** |
| Male | 65 to 84 | 81.8308 | 0 | **18.1692 (beta: 9.085, 40.915)*** |
| Female | 15 to 54 | 94.3242 | 1.59506 | **0** |
| Female | 55 to 59 | 93.8284 | 0 | **6.17156 (beta: 3.08578, 46.914)*** |
| Female | 60 to 84 | 91.927 | 0 | **8.07301 (beta: 4.037, 45.964)*** |
| **Bangladesh** |  |  |  |  |
| Male | 15 to 84 | 90.1799 | 3.7139 | **6.10625 (beta: 26.489, 407.314)** |
| Female | 15 to 84 | 98.4258 | 0.302266 | **1.27193 (beta: 12.082, 937.837)** |

*assumed sample size of 50

As the risk of mortality, morbidity and relapse is dependant on the time since quitting, the distribution of former users across states reflecting their time since quitting was also required. Former ST users begin in the model state that reflects their time since quitting ST. (Table B.3 and B.4)

Note: The “time since quitting” as reported below is specific to the population distribution only upon entering the model. It relates only to the variability in the population at the entry into the model rather than uncertainty and it does not in any way affect how the cohort transitions through the model. There was little data to inform these distributions and as it will have very little impact on the results it was not considered uncertain.

**Table B.3: India and Bangladesh: Time Since Quitting for Former Users Upon Entering the Model (the distribution for Bangladesh was equated to the distribution for India as information for Bangladesh was not available)**^2^

| **Age at Entering Model** | **Years Since Quitting** | | | | | | | | | |
| --- | --- | --- | --- | --- | --- | --- | --- | --- | --- | --- |
|  | **1** | **2** | **3** | **4** | **5** | **6** | **7** | **8** | **9** | **10 plus** |
| Males |  |  |  |  |  |  |  |  |  |  |
| 15 to 19 | 0.690 | 0.310 | 0.000 | 0.000 | 0.000 | 0.000 | 0.000 | 0.000 | 0.000 | 0.000 |
| 20 to 24 | 0.385 | 0.390 | 0.092 | 0.133 | 0.000 | 0.000 | 0.000 | 0.000 | 0.000 | 0.000 |
| 25 to 29 | 0.187 | 0.135 | 0.364 | 0.049 | 0.021 | 0.180 | 0.007 | 0.000 | 0.000 | 0.056 |
| 30 to 34 | 0.162 | 0.196 | 0.011 | 0.150 | 0.169 | 0.000 | 0.117 | 0.000 | 0.120 | 0.072 |
| 35 to 39 | 0.179 | 0.058 | 0.125 | 0.045 | 0.078 | 0.178 | 0.066 | 0.000 | 0.034 | 0.236 |
| 40 to 44 | 0.040 | 0.198 | 0.050 | 0.182 | 0.146 | 0.000 | 0.112 | 0.000 | 0.013 | 0.258 |
| 45 to 49 | 0.101 | 0.033 | 0.236 | 0.201 | 0.057 | 0.030 | 0.021 | 0.000 | 0.140 | 0.181 |
| 50 to 54 | 0.036 | 0.017 | 0.040 | 0.065 | 0.076 | 0.022 | 0.183 | 0.055 | 0.086 | 0.420 |
| 55 to 59 | 0.037 | 0.146 | 0.090 | 0.043 | 0.000 | 0.165 | 0.027 | 0.300 | 0.000 | 0.193 |
| 60 to 64 | 0.082 | 0.172 | 0.041 | 0.021 | 0.199 | 0.000 | 0.000 | 0.000 | 0.137 | 0.348 |
| 65 to 69 | 0.318 | 0.033 | 0.077 | 0.091 | 0.000 | 0.014 | 0.017 | 0.000 | 0.048 | 0.402 |
| 70 to 74 | 0.010 | 0.014 | 0.023 | 0.064 | 0.034 | 0.002 | 0.206 | 0.031 | 0.080 | 0.535 |
| 75 to 79 | 0.019 | 0.043 | 0.014 | 0.044 | 0.090 | 0.022 | 0.000 | 0.000 | 0.015 | 0.754 |
| 80 to 84 | 0.000 | 0.000 | 0.000 | 0.012 | 0.193 | 0.000 | 0.004 | 0.000 | 0.000 | 0.790 |
| Females |  |  |  |  |  |  |  |  |  |  |
| 15 to 19 | 0.000 | 0.498 | 0.502 | 0.000 | 0.000 | 0.000 | 0.000 | 0.000 | 0.000 | 0.000 |
| 20 to 24 | 0.112 | 0.510 | 0.099 | 0.000 | 0.279 | 0.000 | 0.000 | 0.000 | 0.000 | 0.000 |
| 25 to 29 | 0.024 | 0.196 | 0.128 | 0.000 | 0.198 | 0.145 | 0.000 | 0.308 | 0.000 | 0.000 |
| 30 to 34 | 0.053 | 0.141 | 0.095 | 0.127 | 0.047 | 0.024 | 0.000 | 0.000 | 0.095 | 0.419 |
| 35 to 39 | 0.217 | 0.620 | 0.000 | 0.036 | 0.000 | 0.000 | 0.007 | 0.054 | 0.001 | 0.064 |
| 40 to 44 | 0.065 | 0.036 | 0.005 | 0.000 | 0.330 | 0.086 | 0.132 | 0.028 | 0.052 | 0.265 |
| 45 to 49 | 0.326 | 0.267 | 0.045 | 0.038 | 0.000 | 0.000 | 0.028 | 0.000 | 0.000 | 0.297 |
| 50 to 54 | 0.281 | 0.043 | 0.008 | 0.150 | 0.000 | 0.147 | 0.000 | 0.000 | 0.000 | 0.370 |
| 55 to 59 | 0.177 | 0.137 | 0.285 | 0.101 | 0.042 | 0.103 | 0.000 | 0.000 | 0.007 | 0.147 |
| 60 to 64 | 0.016 | 0.038 | 0.080 | 0.016 | 0.012 | 0.000 | 0.020 | 0.000 | 0.176 | 0.641 |
| 65 to 69 | 0.105 | 0.136 | 0.407 | 0.186 | 0.043 | 0.001 | 0.000 | 0.000 | 0.000 | 0.120 |
| 70 to 74 | 0.254 | 0.030 | 0.003 | 0.000 | 0.012 | 0.000 | 0.000 | 0.000 | 0.153 | 0.549 |
| 75 to 79 | 0.000 | 0.000 | 0.000 | 0.489 | 0.008 | 0.000 | 0.000 | 0.396 | 0.000 | 0.107 |
| 80 to 84 | 0.000 | 0.000 | 0.000 | 0.000 | 0.041 | 0.203 | 0.054 | 0.000 | 0.000 | 0.701 |

**Table B.4: Pakistan - Time Since Quitting for Former Users Upon Entering the Model**^4^

| **Age at Entering Model** | **Years Since Quitting** | | | | | | | | | |
| --- | --- | --- | --- | --- | --- | --- | --- | --- | --- | --- |
|  | **1** | **2** | **3** | **4** | **5** | **6** | **7** | **8** | **9** | **10 plus** |
| Males |  |  |  |  |  |  |  |  |  |  |
| 15 to 19 | 0.000 | 1.000 | 0.000 | 0.000 | 0.000 | 0.000 | 0.000 | 0.000 | 0.000 | 0.000 |
| 20 to 24 | 0.000 | 1.000 | 0.000 | 0.000 | 0.000 | 0.000 | 0.000 | 0.000 | 0.000 | 0.000 |
| 25 to 29 | 0.000 | 0.000 | 0.000 | 0.000 | 0.024 | 0.976 | 0.000 | 0.000 | 0.000 | 0.000 |
| 30 to 34 | 0.000 | 0.000 | 0.000 | 0.000 | 0.024 | 0.976 | 0.000 | 0.000 | 0.000 | 0.000 |
| 35 to 39 | 0.000 | 0.000 | 0.000 | 0.000 | 0.024 | 0.976 | 0.000 | 0.000 | 0.000 | 0.000 |
| 40 to 44 | 0.000 | 0.000 | 0.000 | 0.000 | 0.000 | 0.515 | 0.000 | 0.000 | 0.485 | 0.000 |
| 45 to 49 | 0.000 | 0.000 | 0.000 | 0.000 | 0.000 | 0.515 | 0.000 | 0.000 | 0.485 | 0.000 |
| 50 to 54 | 0.000 | 0.000 | 0.000 | 0.000 | 0.000 | 0.800 | 0.000 | 0.179 | 0.021 | 0.000 |
| 55 to 59 | 0.000 | 0.000 | 0.000 | 0.000 | 0.000 | 0.000 | 0.101 | 0.000 | 0.000 | 0.899 |
| 60 to 64 | 0.000 | 0.000 | 0.000 | 0.000 | 0.000 | 0.000 | 0.000 | 0.000 | 0.000 | 1.000 |
| 65 to 69 | 0.000 | 0.000 | 0.000 | 0.000 | 0.000 | 0.000 | 0.000 | 0.000 | 0.000 | 1.000 |
| 70 to 74 | 0.000 | 0.000 | 0.000 | 0.000 | 0.000 | 0.000 | 0.000 | 0.000 | 0.000 | 1.000 |
| 75 to 79 | 0.000 | 0.000 | 0.000 | 0.000 | 0.000 | 0.000 | 0.000 | 0.000 | 0.000 | 1.000 |
| 80 to 84 | 0.000 | 0.000 | 0.000 | 0.012 | 0.193 | 0.000 | 0.004 | 0.000 | 0.000 | 0.790 |
| Females |  |  |  |  |  |  |  |  |  |  |
| 15 to 19 | 0.000 | 0.000 | 0.000 | 0.000 | 0.000 | 0.000 | 0.000 | 0.000 | 0.000 | 0.000 |
| 20 to 24 | 0.000 | 0.000 | 0.000 | 0.000 | 0.000 | 0.000 | 0.000 | 0.000 | 0.000 | 0.000 |
| 25 to 29 | 0.000 | 0.000 | 0.000 | 0.000 | 0.000 | 0.000 | 0.000 | 0.000 | 0.000 | 0.000 |
| 30 to 34 | 0.000 | 0.000 | 0.000 | 0.000 | 0.000 | 0.000 | 0.000 | 0.000 | 0.000 | 0.000 |
| 35 to 39 | 0.000 | 0.000 | 0.000 | 0.000 | 0.000 | 0.000 | 0.000 | 0.000 | 0.000 | 0.000 |
| 40 to 44 | 0.000 | 0.000 | 0.000 | 0.000 | 0.000 | 0.000 | 0.000 | 0.000 | 0.000 | 0.000 |
| 45 to 49 | 0.000 | 0.000 | 0.000 | 0.000 | 0.000 | 0.000 | 0.000 | 0.000 | 0.000 | 0.000 |
| 50 to 54 | 0.000 | 0.000 | 0.000 | 0.000 | 0.000 | 0.000 | 0.000 | 0.000 | 0.000 | 0.000 |
| 55 to 59 | 0.000 | 0.000 | 0.000 | 0.000 | 0.000 | 0.000 | 0.000 | 0.000 | 0.000 | 1.000 |
| 60 to 64 | 0.000 | 0.000 | 0.000 | 0.000 | 0.000 | 0.000 | 0.000 | 0.000 | 0.000 | 1.000 |
| 65 to 69 | 0.000 | 0.000 | 0.000 | 0.000 | 1.000 | 0.000 | 0.000 | 0.000 | 0.000 | 0.000 |
| 70 to 74 | 0.000 | 0.000 | 0.000 | 0.000 | 0.000 | 0.000 | 0.000 | 0.000 | 0.000 | 1.000 |
| 75 to 79 | 0.000 | 0.000 | 0.000 | 0.000 | 0.000 | 0.000 | 0.000 | 0.000 | 0.000 | 1.000 |
| 80 to 84 | 0.000 | 0.000 | 0.000 | 0.000 | 0.000 | 0.000 | 0.000 | 0.000 | 0.000 | 1.000 |

**B.2 Annual Uptake of ST**

The rate at which individuals initiate ST in each of the age brackets was estimated based on data from the GATS.^2–4^ As data is available to allow calculation of the number of individuals who were using ST at the start of the age interval and the number of individuals who initiated ST within the age interval, the rate of ST uptake within each age interval can be calculated. We assume that the rate of uptake of ST in each age bracket is consistent over time and we estimate the uptake in further age ranges by converting the rates to transition probabilities. This is equivalent to assuming that current 15 to 19 years olds will initiate ST at the same rate when they are between 20 to 24 years of age as the current cohort of 20 to 24 year olds report initiating ST within the survey. This incorporates an assumption that the best available information to predict the rate of future uptake is the rate of current uptake in the respective age brackets thereby enabling estimation of the uptake of ST or the transitions from never ST user to current/former users over the model timeframe. The resultant estimated probability of uptake of ST by age range and sex for each country is reported in Table B.5.

**Table B.5: Annual probability of uptake of smokeless tobacco by sex and age bracket**^2–4^

| **Sex** | **Age range** | **India** | **Pakistan** | **Bangladesh** |
| --- | --- | --- | --- | --- |
| Males | 15 to 19 | 0.0283 (beta: 67.35, 2311.48) | 0.0060 (beta: 2.95, 485.90) | 0.0089 (beta: 3.41, 379.02) |
|  | 20 to 24 | 0.0301 (beta: 57.59, 1858.34) | 0.0153 (beta: 4.99, 320.73) | 0.0200 (beta: 7.11, 349.18) |
|  | 25 to 29 | 0.0193 (beta: 33.49, 1702.70) | 0.0055 (beta: 2.34, 420.60) | 0.0216 (beta: 9.01, 408.58) |
|  | 30 to 34 | 0.0051 (beta: 18.04, 3512.56) | 0.0039 (beta: 2.40, 616.16) | 0.0155 (beta: 6.52, 415.29) |
|  | 35 to 39 | 0.0042 (beta: 8.94, 2121.66) | 0.0007 (beta: 12.77, 17847.12) | 0.0173 (beta: 9.75, 552.42) |
|  | 40 to 44 | 0.0053 (beta: 10.94, 2049.99) | 0.0008 (beta: 17.72, 21018.20) | 0.0227 (beta: 9.66, 416.59) |
|  | 45 to 49 | 0.0034 (beta: 6.72, 1984.79) | 0 | 0.0234 (beta: 5.68, 236.59) |
|  | 50 to 54 | 0 | 0 | 0.0364 (beta: 7.41, 196.03) |
| Females | 15 to 19 | 0.0052 (beta: 13.91, 2643.02) | 0.0024 (beta: 0.94, 386.54) | 0.0049 (beta: 3.56, 719.57) |
|  | 20 to 24 | 0.0046 (beta: 20.88, 4537.21) | 0.0005 (beta: 0.60, 1105.76) | 0.0219 (beta: 16.07, 717.54) |
|  | 25 to 29 | 0.0053 (beta: 19.08, 3611.30) | 0.0012 (beta: 0.75, 617.00) | 0.0120 (beta: 16.85, 1389.55) |
|  | 30 to 34 | 0.0050 (beta: 20.63, 4097.61) | 0.0000 | 0.0499 (beta: 33.48, 637.43) |
|  | 35 to 39 | 0.0041 (beta: 17.06, 4103.70) | 0.0023 (beta: 1.04, 441.09) | 0.0327 (beta: 26.05, 770.19) |
|  | 40 to 44 | 0.0041 (beta: 9.78, 2390.08) | 0.0021 (beta: 4.37, 2082.84) | 0.0327 (beta: 13.60, 402.79) |
|  | 45 to 49 | 0.0048 (beta: 11.71, 2426.28) | 0.0029 (beta: 1.26, 429.11) | 0.0324 (beta: 11.69, 349.49) |
|  | 50 to 54 | 0.0019 (beta: 3.71, 1919.08) | 0.0039 (beta: 0.86, 218.46) | 0.0495 (beta: 7.74, 148.56) |
|  | 55 to 59 | 0.0026 (beta: 4.04, 1539.39) | 0.0009 (beta: 0.69, 753.79) | 0.0531 (beta: 4.95, 88.25) |
|  | 60 to 64 | 0.0049 (beta: 4.48, 913.31) | 0 | 0 |
|  | 65 to 69 | 0 | 0 | 0 |

**B.3 Annual Probability of Quit Attempts and Quit Success**

The transitions between current and former users requires estimation of the probability of current ST users making a quit attempt each year, the proportion of quit attempts that are successful and the rate of relapse from former ST user to current ST user in subsequent years. The GATS surveys provide data to allow estimation of the proportion of individuals making a quit attempt each year and the proportion that are successful.^2–4^ Relapse in the first year of quitting can therefore be estimated directly from the responses to the GATS survey. (Table B.6 and B.7) The modelling approach taken incorporates the assumption of a consistent rate of quit attempts and quit success over time based on the age and sex of the cohort; i.e. as the cohort that enters the model between the ages of 15 and 19 progresses through the model, they are assumed to quit at the quit rate of current 20 to 24 year olds for cycles in which they are 20 to 24 years. This assumption is grounded in the understanding that our best prediction of the future is based on the current trend by age and sex.

**Table B.6: Annual Probability of No Quit Attempt By Age, Sex and Country (Quit Attempt probability equals 1 minus this value)**

| **Sex** | **Age Range** | **Annual Probability of No Quit Attempt** |
| --- | --- | --- |
| **India** |  |  |
| Males | 15 to 19 | 0.572 (beta: 533.03, 399.35) |
|  | 20 to 24 | 0.587 (beta: 709.40, 498.19) |
|  | 25 to 29 | 0.603 (beta: 956.77, 629.35) |
|  | 30 to 34 | 0.619 (beta: 1289.26, 793.61) |
|  | 35 to 39 | 0.635 (beta: 1678.67, 965.97) |
|  | 40 to 44 | 0.651 (beta: 1993.02, 1070.76) |
|  | 45 to 49 | 0.666 (beta: 2033.90, 1018.74) |
|  | 50 to 54 | 0.682 (1769.80, 825.06) |
|  | 55 to 59 | 0.698 (beta: 1382.85, 598.86) |
|  | 60 to 64 | 0.714 (beta: 1031.74, 414.15) |
|  | 65 to 69 | 0.729 (beta: 764.06, 283.55) |
|  | 70 to 74 | 0.745 (beta: 571.41, 195.48) |
|  | 75 to 79 | 0.761 (beta: 433.89, 136.37) |
|  | 80 to 84 | 0.777 (beta: 334.56, 96.22) |
|  | 85 to 89 | 0.792 (beta: 261.42, 68.49) |
| Females | 15 to 19 | 0.627 (beta: 289.94, 172.18) |
|  | 20 to 24 | 0.642 (beta: 375.26, 209.65) |
|  | 25 to 29 | 0.656 (beta: 492.28, 258.47) |
|  | 30 to 34 | 0.670 (beta: 648.42, 319.55) |
|  | 35 to 39 | 0.684 (beta: 838.15, 387.17) |
|  | 40 to 44 | 0.698 (beta: 1018.38, 440.25) |
|  | 45 to 49 | 0.712 (beta: 1100.16, 444.30) |
|  | 50 to 54 | 0.726 (beta: 1023.69, 385.42) |
|  | 55 to 59 | 0.741 (beta: 839.42, 293.96) |
|  | 60 to 64 | 0.755 (beta: 640.46, 208.08) |
|  | 65 to 69 | 0.769 (beta: 476.10, 143.07) |
|  | 70 to 74 | 0.783 (beta: 353.72, 97.98) |
|  | 75 to 79 | 0.797 (beta: 265.44, 67.51) |
|  | 80 to 84 | 0.811 (beta: 201.76, 46.90) |
|  | 85 to 89 | 0.826 (beta: 155.21, 32.80) |
| **Pakistan** |  |  |
| Males | 15 to 24 | 0.809 (beta: 19.02, 4.48) |
|  | 25 to 44 | 0.788 (beta: 74.41, 19.99) |
|  | 45 to 64 | 0.837 (beta: 42.60, 8.33) |
|  | 65 to 79 | 0.933 (beta: 7.14, 0.516) |
|  | 80 to 89 | 1 |
| Females | 15 to 24 | 0.986 (beta: 38.41, 0.56) |
|  | 25 to 44 | 0.877 (beta: 47.77, 6.70) |
|  | 45 to 64 | 0.580 (beta: 26.90, 19.46) |
|  | 65 to 79 | 0.595 (beta: 3.16, 2.15) |
|  | 80 to 89 | 1 |
| **Bangladesh** |  |  |
| Males | 15 to 79 | 0.731 (beta: 348.37, 128.48) |
|  | 80 to 89 | 1 |
| Females | 15 to 79 | 0.705 (beta: 432.05, 180.46) |
|  | 80 to 89 | 1 |

**Table B.7: Probability that Quit Attempt is Successful**

| **Sex** | **Country** | | |
| --- | --- | --- | --- |
|  | **India** | **Pakistan** | **Bangladesh** |
| Males | 0.065 (beta: 44.50, 645.38) | 0.063 (beta: 5.65, 84.53) | 0.083 (beta: 15.45, 170.63) |
| Females | 0.080 (beta: 27.61, 315.79) | 0.053 (beta: 0.67, 11.94) | 0.055 (beta: 6.38, 109.82) |

**B.4 The annual probability of relapse to reinitiate ST in years after quitting ST**

It is clear given the very low percentage of former ST users over the life course that there is continued relapse by former ST users in subsequent years. Data regarding relapse in subsequent years was not collected as part of the GATS surveys but was estimated through model calibration.

The percentage of former ST users is persistently low over the life course (generally <5%) in India. (Figure B.1 and B.2) Although based on more limited data, similar low percentages were observed for Pakistan and Bangladesh.

**Figure B.1 India: Percentage of males who are former smokeless tobacco users by age**^2^

**Figure B.2 India: Percentage of females who are former smokeless tobacco users by age**^2^

The percentage of former ST users by age and sex has been persistently low over time based on the comparison of data from the 2009/2010 GATs and the 2016/2017 GATs surveys for India. (See Figures B.3 and B.4) As such, within the model we assume that the percentage of former ST users by age will be consistent over the long term and calibrate the model to reflect the reported prevalence.

**Figure B.3 India: Percentage of males who are former ST users – 2009/2010 GATS survey compared with 2016/2017 GATS survey**^2,5^

**Figure B.4 India: Percentage of females who are former ST users – 2009/2010 GATS survey**^2,5^

The relapse rates subsequent to the year of quitting were programmed after incorporating differential mortality by ST use status within the model. (see Appendix C for details) Decreasing relapse rates specific to year 2, year 3 and for year 4 onwards subsequent to the quit year were estimated. The proportional decrease in relapse rates from year 2 to year 3 and year 4 onwards was based on the shape of the relapse curve for those who have quit smoking.^6^ An adjustment factor was applied to the relapse probabilities for smoking relapse (0.14 in Year 2, 0.10 in Year 3 and 0.02 in Year 4 onwards) to minimise the difference between the model estimates of the prevalence of former ST users by age and sex cohort from the GATs estimates. The adjustment factor was calibrated using the Solver function in Excel. (Table B.8)

**Table B.8: Relapse Rates Year 2, 3 and 4 through 10 years since quitting (SE estimated at 10% of the mean)**

| **Sex** | **Age Range** | **Relapse Rates by Year Since Quitting** | | |
| --- | --- | --- | --- | --- |
|  |  | **Year 2** | **Year 3** | **Year 4 to 10** |
| **India** |  |  |  |  |
| Male | 15 to 19 | 0.645  (lognormal: -0.444, 0.100) | 0.465  (lognormal: -0.770, 0.100) | 0.111  (lognormal: -2.203, 0.100) |
| Male | 20 to 24 | 0.670  (lognormal: -0.405, 0.100) | 0.484  (lognormal: -0.731, 0.100) | 0.115  (lognormal: -2.164, 0.100) |
| Male | 25 to 29 | 0.668  (lognormal: -0.409, 0.100) | 0.482  (lognormal: -0.735, 0.100) | 0.115  (lognormal: -2.168, 0.100) |
| Male | 30 to 34 | 0.653  (lognormal: -0.431, 0.100) | 0.472  (lognormal: -0.757, 0.100) | 0.112  (lognormal: -2.190, 0.100) |
| Male | 35 to 39 | 0.643  (lognormal: -0.447, 0.100) | 0.464  (lognormal: -0.772, 0.100) | 0.111  (lognormal: -2.206, 0.100) |
| Male | 40 to 44 | 0.572  (lognormal: -0.564, 0.100) | 0.413  (lognormal: -0.890, 0.100) | 0.098  (lognormal: -2.323, 0.100) |
| Male | 45 to 49 | 0.547  (lognormal: -0.607, 0.100) | 0.395  (lognormal: -0.933, 0.100) | 0.094  (lognormal: -2.367, 0.100) |
| Male | 50 to 54 | 0.511  (lognormal: -0.676, 0.100) | 0.369  (lognormal: -1.002, 0.100) | 0.088  (lognormal: -2.435, 0.100) |
| Male | 55 to 59 | 0.404  (lognormal: -0.910, 0.100) | 0.292  (lognormal: -1.236, 0.100) | 0.070  (lognormal: -2.670, 0.100) |
| Male | 60 to 64 | 0.384  (lognormal: -0.963, 0.100) | 0.277  (lognormal: -1.289, 0.100) | 0.066  (lognormal: -2.711, 0.100) |
| Male | 65 to 69 | 0.349  (lognormal: -1.058, 0.100) | 0.252  (lognormal: -1.384, 0.100) | 0.060  (lognormal: -2.817, 0.100) |
| Male | 70 to 74 | 0.684  (lognormal: -0.384, 0.100) | 0.494  (lognormal: -0.710, 0.100) | 0.11  8 (lognormal: -2.143, 0.100) |
| Male | 75 to 79 | 0.990  (lognormal: -0.015, 0.100) | 0.715  (lognormal: -0.341, 0.100) | 0.170  (lognormal: -1.774, 0.100) |
| Male | 80 to 84 | 0.000 | 0.000 | 0.000 |
| Female | 15 to 19 | 0.512  (lognormal: -0.674, 0.100) | 0.370  (lognormal: -1.000, 0.100) | 0.088  (lognormal: -2.434, 0.100) |
| Female | 20 to 24 | 0.516  (lognormal: -0.667, 0.100) | 0.372  (lognormal: -0.993, 0.100) | 0.089  (lognormal: -2.434, 0.100) |
| Female | 25 to 29 | 0.533  (lognormal: -0.635, 0.100) | 0.384  (lognormal: -0.961, 0.100) | 0.092  (lognormal: -2.394, 0.100) |
| Female | 30 to 34 | 0.527  (lognormal: -0.645, 0.100) | 0.381  (lognormal: -0.971, 0.100) | 0.091  (lognormal: -2.404, 0.100) |
| Female | 35 to 39 | 0.512  (lognormal: -0.675, 0.100) | 0.369  (lognormal: -1.001, 0.100) | 0.088  (lognormal: -2.424, 0.100) |
| Female | 40 to 44 | 0.567  (lognormal: -0.572, 0.100) | 0.409  (lognormal: -0.898, 0.100) | 0.098  (lognormal: -2.331, 0.100) |
| Female | 45 to 49 | 0.538  (lognormal: -0.626, 0.100) | 0.388  (lognormal: -0.952, 0.100) | 0.093  (lognormal: -2.385, 0.100) |
| Female | 50 to 54 | 0.490  (lognormal: -0.718, 0.100) | 0.354  (lognormal: -1.044, 0.100) | 0.084  (lognormal: -2.477, 0.100) |
| Female | 55 to 59 | 0.430  (lognormal: -0.848, 0.100) | 0.311  (lognormal: -1.174, 0.100) | 0.074  (lognormal: -2.607, 0.100) |
| Female | 60 to 64 | 0.503  (lognormal: -0.691, 0.100) | 0.363  (lognormal: -1.017, 0.100) | 0.087  (lognormal: -2.450, 0.100) |
| Female | 65 to 69 | 0.347  (lognormal: -1.064, 0.100) | 0.250  (lognormal: -1.390, 0.100) | 0.060  (lognormal: -2.823, 0.100) |
| Female | 70 to 74 | 0.317  (lognormal: -1.153, 0.100) | 0.229  (lognormal: -1.478, 0.100) | 0.055  (lognormal: -2.912, 0.100) |
| Female | 75 to 79 | 0.000 | 0.000 | 0.000 |
| Female | 80 to 84 | 0.000 | 0.000 | 0.000 |
| **Pakistan** |  |  |  |  |
| Male | 15 to 19 | 0.513  (lognormal: -0.672, 0.100) | 0.370  (lognormal: -0.999, 0.100) | 0.088  (lognormal: -2.431, 0.100) |
| Male | 20 to 24 | 0.532  (lognormal: -0.635, 0.100) | 0.384  (lognormal: -0.961, 0.100) | 0.092  (lognormal: -2.394, 0.100) |
| Male | 25 to 29 | 0.465  (lognormal: -0.772, 0.100) | 0.335  (lognormal: -1.098, 0.100) | 0.080  (lognormal: -2.531, 0.100) |
| Male | 30 to 34 | 0.469  (lognormal: -0.763, 0.100) | 0.338  (lognormal: -1.088, 0.100) | 0.081  (lognormal: -2.522, 0.100) |
| Male | 35 to 39 | 0.412  (lognormal: -0.891, 0.100) | 0.298  (lognormal: -1.217, 0.100) | 0.071  (lognormal: -2.650, 0.100) |
| Male | 40 to 44 | 0.392  (lognormal: -0.942, 0.100) | 0.283  (lognormal: -1.268, 0.100) | 0.067  (lognormal: -2.701, 0.100) |
| Male | 45 to 49 | 0.471  (lognormal: -0.758, 0.100) | 0.340  (lognormal: -1.084, 0.100) | 0.081  (lognormal: -2.517, 0.100) |
| Male | 50 to 54 | 0.449  (lognormal: -0.805, 0.100) | 0.324  (lognormal: -1.131, 0.100) | 0.077  (lognormal: -2.564, 0.100) |
| Male | 55 to 59 | 0.477  (lognormal: -0.744, 0.100) | 0.345  (lognormal: -1.040, 0.100) | 0.082  (lognormal: -2.504, 0.100) |
| Male | 60 to 64 | 0.201  (lognormal: -1.608, 0.100) | 0.145  (lognormal: -1.934, 0.100) | 0.035  (lognormal: -3.367, 0.100) |
| Male | 65 to 69 | 1.000  (lognormal: -0.005, 0.100) | 0.722  (lognormal: -0.331, 0.100) | 0.172  (lognormal: -1.764, 0.100) |
| Male | 70 to 74 | 1.000  (lognormal: -0.005, 0.100) | 0.722  (lognormal: -0.331, 0.100) | 0.172  (lognormal: -1.764, 0.100) |
| Male | 75 to 79 | 0.990  (lognormal: -0.015, 0.100) | 0.715  (lognormal: -0.341, 0.100) | 0.170  (lognormal: -1.774, 0.100) |
| Male | 80 to 84 | 0.927  (lognormal: -0.081, 0.100) | 0.669  (lognormal: -0.407, 0.100) | 0.160  (lognormal: -1.840, 0.100) |
| Female | 15 to 19 | 0.509  (lognormal: -0.681, 0.100) | 0.367  (lognormal: -1.007, 0.100) | 0.088  (lognormal: -2.440, 0.100) |
| Female | 20 to 24 | 0.458  (lognormal: -0.786, 0.100) | 0.330  (lognormal: -1.112, 0.100) | 0.079  (lognormal: -2.545, 0.100) |
| Female | 25 to 29 | 0.435  (lognormal: -0.837, 0.100) | 0.314  (lognormal: -1.163, 0.100) | 0.075  (lognormal: -2.596, 0.100) |
| Female | 30 to 34 | 0.405  (lognormal: -0.908, 0.100) | 0.293  (lognormal: -1.234, 0.100) | 0.070  (lognormal: -2.667, 0.100) |
| Female | 35 to 39 | 0.367  (lognormal: -1.007, 0.100) | 0.265  (lognormal: -1.333, 0.100) | 0.063  (lognormal: -2.766, 0.100) |
| Female | 40 to 44 | 0.489  (lognormal: -0.720, 0.100) | 0.353  (lognormal: -1.047, 0.100) | 0.084  (lognormal: -2.480, 0.100) |
| Female | 45 to 49 | 0.429  (lognormal: -0.851, 0.100) | 0.310  (lognormal: -1.177, 0.100) | 0.074  (lognormal: -2.610, 0.100) |
| Female | 50 to 54 | 0.569  (lognormal: -0.569, 0.100) | 0.411  (lognormal: -0.895, 0.100) | 0.098  (lognormal: -2.328, 0.100) |
| Female | 55 to 59 | 0.861  (lognormal: -0.154, 0.100) | 0.622  (lognormal: -0.480, 0.100) | 0.148  (lognormal: -1.913, 0.100) |
| Female | 60 to 64 | 0.747  (lognormal: -0.296, 0.100) | 0.539  (lognormal: -0.622, 0.100) | 0.129  (lognormal: -2.056, 0.100) |
| Female | 65 to 69 | 0.520  (lognormal: -0.660, 0.100) | 0.375  (lognormal: -0.985, 0.100) | 0.090  (lognormal: -2.418, 0.100) |
| Female | 70 to 74 | 1.000  (lognormal: -0.005, 0.100) | 0.722  (lognormal: -0.331, 0.100) | 0.172  (lognormal: -1.764, 0.100) |
| Female | 75 to 79 | 1.000  (lognormal: -0.005, 0.100) | 0.722  (lognormal: -0.331, 0.100) | 0.172  (lognormal: -1.764, 0.100) |
| Female | 80 to 84 | 0.000 | 0.000 | 0.000 |
| **Bangladesh** |  |  |  |  |
| Male | 15 to 19 | 0.607  (lognormal: -0.505, 0.100) | 0.438  (lognormal: -0.831, 0.100) | 0.104  (lognormal: -2.264, 0.100) |
| Male | 20 to 24 | 0.633  (lognormal: -0.462, 0.100) | 0.457  (lognormal: -0.788, 0.100) | 0.109  (lognormal: -2.221, 0.100) |
| Male | 25 to 29 | 0.599  (lognormal: -0.518, 0.100) | 0.432  (lognormal: -0.843, 0.100) | 0.103  (lognormal: -2.277, 0.100) |
| Male | 30 to 34 | 0.652  (lognormal: -0.433, 0.100) | 0.471  (lognormal: -0.759, 0.100) | 0.112  (lognormal: -2.192, 0.100) |
| Male | 35 to 39 | 0.624  (lognormal: -0.477, 0.100) | 0.450  (lognormal: -0.803, 0.100) | 0.107  (lognormal: -2.236, 0.100) |
| Male | 40 to 44 | 0.636  (lognormal: -0.457, 0.100) | 0.459  (lognormal: -0.783, 0.100) | 0.110  (lognormal: -2.216, 0.100) |
| Male | 45 to 49 | 0.581  (lognormal: -0.549, 0.100) | 0.419  (lognormal: -0.875, 0.100) | 0.100  (lognormal: -2.308, 0.100) |
| Male | 50 to 54 | 0.585  (lognormal: -0.541, 0.100) | 0.422  (lognormal: -0.867, 0.100) | 0.101  (lognormal: -2.300, 0.100) |
| Male | 55 to 59 | 0.511  (lognormal: -0.676, 0.100) | 0.369  (lognormal: -1.002, 0.100) | 0.088  (lognormal: -2.435, 0.100) |
| Male | 60 to 64 | 0.507  (lognormal: -0.684, 0.100) | 0.366  (lognormal: -1.010, 0.100) | 0.087  (lognormal: -2.443, 0.100) |
| Male | 65 to 69 | 0.443  (lognormal: -0.819, 0.100) | 0.320  (lognormal: -1.145, 0.100) | 0.076  (lognormal: -2.578, 0.100) |
| Male | 70 to 74 | 0.631  (lognormal: -0.466, 0.100) | 0.455  (lognormal: -0.792, 0.100) | 0.109  (lognormal: -2.225, 0.100) |
| Male | 75 to 79 | 0.374  (lognormal: 0.988, 0.100) | 0.270  (lognormal: -1.314, 0.100) | 0.064  (lognormal: -2.747, 0.100) |
| Male | 80 to 84 | 0.000 | 0.000 | 0.000 |
| Female | 15 to 19 | 0.854  (lognormal: -0.163, 0.100) | 0.617  (lognormal: -0.488, 0.100) | 0.147  (lognormal: -1.922, 0.100) |
| Female | 20 to 24 | 0.860  (lognormal: -0.156, 0.100) | 0.621  (lognormal: -0.482, 0.100) | 0.148  (lognormal: -1.915, 0.100) |
| Female | 25 to 29 | 0.854  (lognormal: -0.162, 0.100) | 0.617  (lognormal: -0.488, 0.100) | 0.147  (lognormal: -1.821, 0.100) |
| Female | 30 to 34 | 0.880  (lognormal: -0.133, 0.100) | 0.635  (lognormal: -0.459, 0.100) | 0.151  (lognormal: -1.892, 0.100) |
| Female | 35 to 39 | 0.841  (lognormal: -0.178, 0.100) | 0.607  (lognormal: -0.504, 0.100) | 0.145  (lognormal: -1.937, 0.100) |
| Female | 40 to 44 | 0.895  (lognormal: -0.116, 0.100) | 0.646  (lognormal: -0.442, 0.100) | 0.154  (lognormal: -1.875, 0.100) |
| Female | 45 to 49 | 0.861  (lognormal: -0.155, 0.100) | 0.622  (lognormal: -0.481, 0.100) | 0.148  (lognormal: -1.914, 0.100) |
| Female | 50 to 54 | 0.920  (lognormal: -0.088, 0.100) | 0.664  (lognormal: -0.414, 0.100) | 0.158  (lognormal: -1.847, 0.100) |
| Female | 55 to 59 | 0.862  (lognormal: 0.154, 0.100) | 0.622  (lognormal: -0.480, 0.100) | 0.148  (lognormal: -1.913, 0.100) |
| Female | 60 to 64 | 1.000  (lognormal: -0.005, 0.100) | 0.722  (lognormal: -0.331, 0.100) | 0.172  (lognormal: -1.764, 0.100) |
| Female | 65 to 69 | 0.902  (lognormal: -0.108, 0.100) | 0.651  (lognormal: -0.434, 0.100) | 0.155  (lognormal: -1.867, 0.100) |
| Female | 70 to 74 | 0.916  (lognormal: -0.092, 0.100) | 0.661  (lognormal: -0.418, 0.100) | 0.158  (lognormal: -1.852, 0.100) |
| Female | 75 to 79 | 0.830  (lognormal: -0.192, 0.100) | 0.599  (lognormal: -0.518, 0.100) | 0.143  (lognormal: -1.951, 0.100) |
| Female | 80 to 84 | 0.000 | 0.000 | 0.000 |

**Appendix C: Differential Mortality**

Mortality data was sourced from standard life tables and the attributable mortality for never, current and former ST users was calculated as a function of both the prevalence of never, current and former ST users and the relative risk of mortality for current and former ST users versus never ST users.^7,8^ The United Nations actuarial life tables provided the estimated mortality probabilities.^4^ ST prevalence was sourced from the country specific GATS surveys.

A recently published systematic review and meta-analysis of observational studies reported a significant association between ST and all cause mortality with an estimated odds ratio of 1.25 (95% CI 1.08 to 1.44) based on studies specific to South East Asia.^9^ No studies were located regarding the relative risk of mortality in former ST users. There is evidence that the risk of ST related diseases are reduced in former ST users versus current users; however, the rate of the reduction in the mortality risk has not been documented. As such, we used data regarding the reduction in risk of mortality in people who have quit smoking to inform the estimated mortality in former ST users.^10^ Relative to current smokers, the risk of mortality is reduced by ~65% over the first 5 years since quitting, by ~90% over years 5 to 10 and approaches those of non users at 10 or more years since quitting. We assumed the same proportional reduction in risk of mortality for former ST users resulting in a relative risk of 1.09 for 1 to 4 years since quitting, 1.03 for years 5 to 9 and 1 for years 10 plus versus never ST users. The mathematical calculations for differential mortality by smokeless tobacco use status are consistent with the calculation of differential disease prevalence and are provided below in Appendix D.

**Table C.1: Mortality Rates by Sex, Age and ST Use Status**

| **Sex** | **Age Range** | **Current ST Users** | **Former ST** | | | **Non ST User** |
| --- | --- | --- | --- | --- | --- | --- |
|  |  |  | **0 to 4 years since quitting** | **5 to 9 years since quitting** | **10 plus years since quitting** |  |
| **India** | | | | | | |
| Male | 15 to 19 | 0.0010502 | 0.0010502 | 0.0010502 | 0.0010502 | 0.0010502 |
| Male | 20 to 24 | 0.0016998 | 0.0016998 | 0.0016998 | 0.0016998 | 0.0016998 |
| Male | 25 to 29 | 0.0020625 | 0.0020625 | 0.0020625 | 0.0020625 | 0.0020625 |
| Male | 30 to 34 | 0.0026621 | 0.0026621 | 0.0026621 | 0.0026621 | 0.0026621 |
| Male | 35 to 39 | 0.0042347 | 0.0036877 | 0.0034708 | 0.0033877 | 0.0033877 |
| Male | 40 to 44 | 0.0056431 | 0.0049128 | 0.0046252 | 0.0045145 | 0.0045145 |
| Male | 45 to 49 | 0.0081185 | 0.0070678 | 0.0066540 | 0.0064948 | 0.0064948 |
| Male | 50 to 54 | 0.0113376 | 0.0098704 | 0.0092924 | 0.0090701 | 0.0090701 |
| Male | 55 to 59 | 0.0171634 | 0.0149422 | 0.0140672 | 0.0137308 | 0.0137307 |
| Male | 60 to 64 | 0.0255757 | 0.0222659 | 0.0209620 | 0.0204605 | 0.0204605 |
| Male | 65 to 69 | 0.0405017 | 0.0352603 | 0.0331955 | 0.0324014 | 0.0324013 |
| Male | 70 to 74 | 0.0606799 | 0.0528272 | 0.0497337 | 0.0485439 | 0.0485439 |
| Male | 75 to 79 | 0.0885944 | 0.0771293 | 0.0726127 | 0.0708756 | 0.0708756 |
| Male | 80 to 84 | 0.1210409 | 0.1053768 | 0.0992061 | 0.0968327 | 0.0968327 |
| Male | 85 to 89 | 0.2011385 | 0.1751088 | 0.1648547 | 0.1609108 | 0.1609108 |
| Female | 15 to 19 | 0.0011768 | 0.0011768 | 0.0011768 | 0.0011768 | 0.0011768 |
| Female | 20 to 24 | 0.0015367 | 0.0015367 | 0.0015367 | 0.0015367 | 0.0015367 |
| Female | 25 to 29 | 0.0015387 | 0.0015387 | 0.0015387 | 0.0015387 | 0.0015387 |
| Female | 30 to 34 | 0.0016434 | 0.0016434 | 0.0016434 | 0.0016434 | 0.0016434 |
| Female | 35 to 39 | 0.0024630 | 0.0021442 | 0.0020187 | 0.0019703 | 0.0019703 |
| Female | 40 to 44 | 0.0031812 | 0.0027695 | 0.0026073 | 0.0025450 | 0.0025450 |
| Female | 45 to 49 | 0.0046187 | 0.0040210 | 0.0037855 | 0.0036950 | 0.0036950 |
| Female | 50 to 54 | 0.0069444 | 0.0060457 | 0.0056917 | 0.0055555 | 0.0055555 |
| Female | 55 to 59 | 0.0111647 | 0.0097198 | 0.0091507 | 0.0089318 | 0.0089318 |
| Female | 60 to 64 | 0.0199884 | 0.0174016 | 0.0163826 | 0.0159907 | 0.0159907 |
| Female | 65 to 69 | 0.0327508 | 0.0285125 | 0.0268428 | 0.0262007 | 0.0262007 |
| Female | 70 to 74 | 0.0518061 | 0.0451018 | 0.0424607 | 0.0414449 | 0.0414449 |
| Female | 75 to 79 | 0.0757571 | 0.0659532 | 0.0620911 | 0.0606056 | 0.0606056 |
| Female | 80 to 84 | 0.1136284 | 0.0989236 | 0.0931308 | 0.0909027 | 0.0909027 |
| Female | 85 to 89 | 0.1981204 | 0.1724813 | 0.1623810 | 0.1584963 | 0.1584963 |
| **Pakistan** | | | | | | |
| Male | 15 to 19 | 0.0049057 | 0.0049057 | 0.0049057 | 0.0049057 | 0.0049057 |
| Male | 20 to 24 | 0.0060797 | 0.0060797 | 0.0060797 | 0.0060797 | 0.0060797 |
| Male | 25 to 29 | 0.0079361 | 0.0079360 | 0.0079361 | 0.0079360 | 0.0079360 |
| Male | 30 to 34 | 0.0099300 | 0.0099300 | 0.0099300 | 0.0099300 | 0.0099300 |
| Male | 35 to 39 | 0.0031682 | 0.0027582 | 0.0025967 | 0.0025346 | 0.0025346 |
| Male | 40 to 44 | 0.0044493 | 0.0038734 | 0.0036467 | 0.0035594 | 0.0035594 |
| Male | 45 to 49 | 0.0066181 | 0.0057616 | 0.0054242 | 0.0052945 | 0.0052945 |
| Male | 50 to 54 | 0.0096360 | 0.0083890 | 0.0078977 | 0.0077088 | 0.0077088 |
| Male | 55 to 59 | 0.0153522 | 0.0133654 | 0.0125828 | 0.0122818 | 0.0122818 |
| Male | 60 to 64 | 0.0244831 | 0.0213147 | 0.0200666 | 0.0195865 | 0.0195865 |
| Male | 65 to 69 | 0.0348259 | 0.0303190 | 0.0285436 | 0.0278607 | 0.0278607 |
| Male | 70 to 74 | 0.0598584 | 0.0521120 | 0.0490604 | 0.0478867 | 0.0478867 |
| Male | 75 to 79 | 0.0942963 | 0.0820932 | 0.0772860 | 0.0754371 | 0.0754371 |
| Male | 80 to 84 | 0.1380391 | 0.1201751 | 0.1131379 | 0.1104312 | 0.1104313 |
| Male | 85 to 89 | 0.2145864 | 0.1868163 | 0.1758767 | 0.1716691 | 0.1716691 |
| Female | 15 to 19 | 0.0040761 | 0.0040761 | 0.0040761 | 0.0040761 | 0.0040761 |
| Female | 20 to 24 | 0.0059947 | 0.0059947 | 0.0059947 | 0.0059947 | 0.0059947 |
| Female | 25 to 29 | 0.0054784 | 0.0054784 | 0.0054784 | 0.0054784 | 0.0054784 |
| Female | 30 to 34 | 0.0069375 | 0.0069375 | 0.0069375 | 0.0069375 | 0.0069375 |
| Female | 35 to 39 | 0.0024023 | 0.0020914 | 0.0019690 | 0.0019219 | 0.0019219 |
| Female | 40 to 44 | 0.0033002 | 0.0028730 | 0.0027048 | 0.0026401 | 0.0026401 |
| Female | 45 to 49 | 0.0050981 | 0.0044383 | 0.0041785 | 0.0040785 | 0.0040785 |
| Female | 50 to 54 | 0.0073906 | 0.0064341 | 0.0060574 | 0.0059125 | 0.0059125 |
| Female | 55 to 59 | 0.0126703 | 0.0110306 | 0.0103847 | 0.0101362 | 0.0101362 |
| Female | 60 to 64 | 0.0228084 | 0.0198567 | 0.0186939 | 0.0182467 | 0.0182467 |
| Female | 65 to 69 | 0.0345992 | 0.0301216 | 0.0283578 | 0.0276793 | 0.0276793 |
| Female | 70 to 74 | 0.0516330 | 0.0449510 | 0.0423188 | 0.0413064 | 0.0413064 |
| Female | 75 to 79 | 0.0907694 | 0.0790228 | 0.0743953 | 0.0726155 | 0.0726155 |
| Female | 80 to 84 | 0.1389405 | 0.1209600 | 0.1138768 | 0.1111524 | 0.1111524 |
| Female | 85 to 89 | 0.2350303 | 0.2046146 | 0.1926327 | 0.1880242 | 0.1880242 |
| **Bangladesh** | | | | | | |
| Male | 15 to 19 | 0.0040761 | 0.0040761 | 0.0040761 | 0.0040761 | 0.0040761 |
| Male | 20 to 24 | 0.0059948 | 0.0059947 | 0.0059947 | 0.0059947 | 0.0059947 |
| Male | 25 to 29 | 0.0054784 | 0.0054784 | 0.0054784 | 0.0054784 | 0.0054784 |
| Male | 30 to 34 | 0.0069375 | 0.0069375 | 0.0069375 | 0.0069375 | 0.0069375 |
| Male | 35 to 39 | 0.0022707 | 0.0019768 | 0.0018610 | 0.0018165 | 0.0018165 |
| Male | 40 to 44 | 0.0029307 | 0.0025514 | 0.0024020 | 0.0023446 | 0.0023446 |
| Male | 45 to 49 | 0.0048133 | 0.0041904 | 0.0039450 | 0.0038507 | 0.0038507 |
| Male | 50 to 54 | 0.0072608 | 0.0063211 | 0.0059510 | 0.0058086 | 0.0058086 |
| Male | 55 to 59 | 0.0111867 | 0.0097390 | 0.0091687 | 0.0089493 | 0.0089493 |
| Male | 60 to 64 | 0.0187779 | 0.0163478 | 0.0153905 | 0.0150223 | 0.0150223 |
| Male | 65 to 69 | 0.0346575 | 0.0301724 | 0.0284056 | 0.0277260 | 0.0277260 |
| Male | 70 to 74 | 0.0484832 | 0.0422088 | 0.0397372 | 0.0387865 | 0.0387865 |
| Male | 75 to 79 | 0.0731634 | 0.0636952 | 0.0599653 | 0.0585307 | 0.0585307 |
| Male | 80 to 84 | 0.1483584 | 0.1291591 | 0.1215957 | 0.1186867 | 0.1186867 |
| Male | 85 to 89 | 0.2509614 | 0.2184840 | 0.2056899 | 0.2007691 | 0.2007691 |
| Female | 15 to 19 | 0.0049780 | 0.0049780 | 0.0049780 | 0.0049780 | 0.0049780 |
| Female | 20 to 24 | 0.0044040 | 0.0044040 | 0.0044040 | 0.0044040 | 0.0044040 |
| Female | 25 to 29 | 0.0048430 | 0.0048430 | 0.0048430 | 0.0048430 | 0.0048430 |
| Female | 30 to 34 | 0.0056770 | 0.0056770 | 0.0056770 | 0.0056770 | 0.0056770 |
| Female | 35 to 39 | 0.0019241 | 0.0016751 | 0.0015770 | 0.0015393 | 0.0015393 |
| Female | 40 to 44 | 0.0023630 | 0.0020572 | 0.0019367 | 0.0018904 | 0.0018904 |
| Female | 45 to 49 | 0.0040773 | 0.0035496 | 0.0033418 | 0.0032618 | 0.0032618 |
| Female | 50 to 54 | 0.0053019 | 0.0046158 | 0.0043455 | 0.0042415 | 0.0042415 |
| Female | 55 to 59 | 0.0092799 | 0.0080790 | 0.0076059 | 0.0074239 | 0.0074239 |
| Female | 60 to 64 | 0.0147542 | 0.0128449 | 0.0120927 | 0.0118034 | 0.0118034 |
| Female | 65 to 69 | 0.0224206 | 0.0195191 | 0.0183761 | 0.0179365 | 0.0179365 |
| Female | 70 to 74 | 0.0422197 | 0.0367560 | 0.0346036 | 0.0337758 | 0.0337758 |
| Female | 75 to 79 | 0.0588059 | 0.0511957 | 0.0481978 | 0.0470447 | 0.0470447 |
| Female | 80 to 84 | 0.0882512 | 0.0768305 | 0.0723314 | 0.0706010 | 0.0706010 |
| Female | 85 to 89 | 0.1278200 | 0.1112786 | 0.1047622 | 0.1022560 | 0.1022560 |

# Appendix D: Adjusting Disease Prevalence and Mortality for ST Use Status^11^

To estimate the age and sex specific disease prevalence by ST use status the following was required:

- Prevalence of the disease, by age and sex in the general population (regardless of ST status) (A) (see Table D.1)
- The odds ratios of each co-morbidity by ST status (ST users versus non users (B) and former ST users versus non-users: 0 to 4 years since quitting (C), 5 to 10 years since quitting (D) and 10 plus years since quitting (E))
- The prevalence of ST use (F).

These can be used to calculate the prevalence of each co-morbidity for current ST users (G), former ST user users in the first 4 years of after quitting (H), former ST users 5 to 10 years after quitting (I), former ST users after 10 plus years after quitting (J) and non ST users (K) by ensuring the following equation is satisfied:

(G*F1)+(H*F2)+(I*F3)+(J*F4)+(K*F5) = A

Where G:K = odds ratio B, H:K = odds ratio C, I:K = odds ratio D, J:K = odds ratio E

This can be illustrated with the example of a 35 year old male from India with oral cancer. The prevalence of oral cancer is 0.0304. The relative risk of oral cancer by ST use status versus non ST users is 5.55 for current ST users, 2.61 for former users who quit 0 to 4 years ago, 1.45 for former users who quit 5 to 10 years ago and 1 for former users who quit more than 10 years ago. The prevalence of ST use for men 35 years of age is 0.3712 for current users, 0.017029 for former users (0 to 4 years since quitting), 0.007234 for former users 5 to 10 years since quitting, 0.006337 for former users 10 plus years since quitting and 0.5981 for non ST users.

If we substitute the prevalence of ST use and the actual prevalence rate:

(G*0.3712)+(H*0.017029)+(I*0.007234)+(J*0.006337)+(K*0.5981)=0.0304

Substituting the odds ratios

(K*5.55*0.3712)+(K*2.61*0.017029)+(K*1.45*0.007234)+(K*1*0.006337)+(K*0.5981)

=0.0304

Solving for K

K=0.0304 / ((5.55*0.3712)+(2.61*0.017029)+(1.45*0.007234)+0.006337+0.5981)

K=0.01119

G=0.06208

H=0.02915

I= 0.01618

J= 0.01119

This process was repeated for each age and gender for all co-morbidities.

**Table D.1: Disease Prevalence**^12^

| **India** |  |  |  |  |  |
| --- | --- | --- | --- | --- | --- |
| **Sex** | **Age** | **Disease** | **Prevalence** | **SE** | **Distribution** |
| Male | 15 to 19 | oesophageal cancer | 0.00E+00 | 0.00E+00 |  |
| Male | 20 to 24 | oesophageal cancer | 2.30E-06 | 3.28E-07 | beta(49.35, 2.14E+07) |
| Male | 25 to 29 | oesophageal cancer | 4.05E-06 | 5.18E-07 | beta(61.20, 1.51E+07) |
| Male | 30 to 34 | oesophageal cancer | 7.70E-06 | 1.02E-06 | beta(57.52, 7.47E+06) |
| Male | 35 to 39 | oesophageal cancer | 1.81E-05 | 2.86E-06 | beta(40.06, 2.21E+06) |
| Male | 40 to 44 | oesophageal cancer | 3.22E-05 | 6.05E-06 | beta(28.25, 8.78E+05) |
| Male | 45 to 49 | oesophageal cancer | 7.76E-05 | 1.61E-05 | beta(23.08, 2.97E+05) |
| Male | 50 to 54 | oesophageal cancer | 1.37E-04 | 2.68E-05 | beta(26.33, 1.92E+05) |
| Male | 55 to 59 | oesophageal cancer | 2.22E-04 | 4.30E-05 | beta(26.61, 1.20E+05) |
| Male | 60 to 64 | oesophageal cancer | 2.46E-04 | 5.15E-05 | beta(22.84, 9.28E+04) |
| Male | 65 to 69 | oesophageal cancer | 2.63E-04 | 5.82E-05 | beta(20.36, 7.75E+04) |
| Male | 70 to 74 | oesophageal cancer | 2.51E-04 | 5.59E-05 | beta(20.07, 8.00E+04) |
| Male | 75 to 79 | oesophageal cancer | 2.60E-04 | 5.49E-05 | beta(22.48, 8.64E+04) |
| Male | 80 to 84 | oesophageal cancer | 2.43E-04 | 4.57E-05 | beta(28.32, 1.16E+05) |
| Male | 85 to 89 | oesophageal cancer | 2.40E-04 | 4.09E-05 | beta(34.43, 1.43E+05) |
| Female | 15 to 19 | oesophageal cancer | 0.00E+00 | 0.00E+00 |  |
| Female | 20 to 24 | oesophageal cancer | 3.63E-06 | 5.95E-07 | beta(37.09, 1.02E+07) |
| Female | 25 to 29 | oesophageal cancer | 5.53E-06 | 8.64E-07 | beta(40.86, 7.39E+06) |
| Female | 30 to 34 | oesophageal cancer | 6.50E-06 | 9.88E-07 | beta(43.22, 6.65E+06) |
| Female | 35 to 39 | oesophageal cancer | 1.13E-05 | 1.55E-06 | beta(53.20, 4.69E+06) |
| Female | 40 to 44 | oesophageal cancer | 2.99E-05 | 4.22E-06 | beta(50.33, 1.68E+06) |
| Female | 45 to 49 | oesophageal cancer | 5.65E-05 | 8.16E-06 | beta(47.87, 8.48E+05) |
| Female | 50 to 54 | oesophageal cancer | 1.08E-04 | 1.77E-05 | beta(37.38, 3.46E+05) |
| Female | 55 to 59 | oesophageal cancer | 1.36E-04 | 2.11E-05 | beta(41.21, 3.04E+05) |
| Female | 60 to 64 | oesophageal cancer | 1.39E-04 | 2.50E-05 | beta(30.86, 2.23E+05) |
| Female | 65 to 69 | oesophageal cancer | 1.54E-04 | 2.77E-05 | beta(30.99, 2.01E+05) |
| Female | 70 to 74 | oesophageal cancer | 1.95E-04 | 3.41E-05 | beta(32.62, 1.68E+05) |
| Female | 75 to 79 | oesophageal cancer | 1.99E-04 | 3.55E-05 | beta(31.26, 1.57E+05) |
| Female | 80 to 84 | oesophageal cancer | 1.86E-04 | 2.92E-05 | beta(40.54, 2.18E+05) |
| Female | 85 to 89 | oesophageal cancer | 1.78E-04 | 3.29E-05 | beta(29.40, 1.65E+05) |
| Male | 15 to 19 | oral cancer | 2.42E-05 | 4.90E-06 | beta(24.36, 1.01E+06) |
| Male | 20 to 24 | oral cancer | 4.52E-05 | 7.51E-06 | beta(36.25, 8.02E+05) |
| Male | 25 to 29 | oral cancer | 8.20E-05 | 1.19E-05 | beta(47.19, 5.75E+05) |
| Male | 30 to 34 | oral cancer | 1.50E-04 | 2.05E-05 | beta(53.51, 3.57E+05) |
| Male | 35 to 39 | oral cancer | 3.04E-04 | 4.08E-05 | beta(55.54, 1.83E+05) |
| Male | 40 to 44 | oral cancer | 3.58E-04 | 5.23E-05 | beta(46.82, 1.31E+05) |
| Male | 45 to 49 | oral cancer | 5.37E-04 | 7.85E-05 | beta(46.82, 8.71E+04) |
| Male | 50 to 54 | oral cancer | 7.92E-04 | 1.12E-04 | beta(50.08, 6.32E+04) |
| Male | 55 to 59 | oral cancer | 1.08E-03 | 1.50E-04 | beta(51.91, 4.79E+04) |
| Male | 60 to 64 | oral cancer | 1.11E-03 | 1.52E-04 | beta(53.42, 4.81E+04) |
| Male | 65 to 69 | oral cancer | 1.24E-03 | 1.66E-04 | beta(56.06, 4.50E+04) |
| Male | 70 to 74 | oral cancer | 1.17E-03 | 1.53E-04 | beta(58.82, 5.01E+04) |
| Male | 75 to 79 | oral cancer | 1.11E-03 | 1.42E-04 | beta(61.42, 5.51E+04) |
| Male | 80 to 84 | oral cancer | 6.97E-04 | 8.57E-05 | beta(66.18, 9.48E+04) |
| Male | 85 to 89 | oral cancer | 5.22E-04 | 5.82E-05 | beta(80.18, 1.54E+05) |
| Female | 15 to 19 | oral cancer | 2.65E-05 | 3.16E-06 | beta(70.41, 2.66E+06) |
| Female | 20 to 24 | oral cancer | 5.11E-05 | 7.30E-06 | beta(49.07, 9.59E+05) |
| Female | 25 to 29 | oral cancer | 6.14E-05 | 9.00E-06 | beta(46.54, 7.58E+05) |
| Female | 30 to 34 | oral cancer | 1.02E-04 | 1.39E-05 | beta(53.92, 5.28E+05) |
| Female | 35 to 39 | oral cancer | 1.43E-04 | 1.92E-05 | beta(55.86, 3.90E+05) |
| Female | 40 to 44 | oral cancer | 2.12E-04 | 2.86E-05 | beta(54.81, 2.59E+05) |
| Female | 45 to 49 | oral cancer | 3.06E-04 | 4.11E-05 | beta(55.66, 1.82E+05) |
| Female | 50 to 54 | oral cancer | 4.90E-04 | 8.50E-05 | beta(33.26, 6.78E+04) |
| Female | 55 to 59 | oral cancer | 6.01E-04 | 8.49E-05 | beta(50.02, 8.32E+04) |
| Female | 60 to 64 | oral cancer | 7.28E-04 | 9.00E-05 | beta(65.36, 8.98E+04) |
| Female | 65 to 69 | oral cancer | 8.09E-04 | 8.99E-05 | beta(80.98, 1.00E+05) |
| Female | 70 to 74 | oral cancer | 8.45E-04 | 9.83E-05 | beta(73.83, 8.73E+04) |
| Female | 75 to 79 | oral cancer | 8.85E-04 | 9.83E-05 | beta(80.97, 9.14E+04) |
| Female | 80 to 84 | oral cancer | 6.19E-04 | 7.36E-05 | beta(70.67, 1.14E+05) |
| Female | 85 to 89 | oral cancer | 4.86E-04 | 5.82E-05 | beta(69.67, 1.43E+05) |
| Male | 15 to 19 | nasopharyngeal cancer | 5.66E-06 | 0.0000009 | beta(36.77, 6.49E+06) |
| Male | 20 to 24 | nasopharyngeal cancer | 7.25E-06 | 0.0000011 | beta(46.01, 6.34E+06) |
| Male | 25 to 29 | nasopharyngeal cancer | 1.06E-05 | 0.0000013 | beta(66.03, 6.25E+06) |
| Male | 30 to 34 | nasopharyngeal cancer | 1.77E-05 | 0.0000019 | beta(89.41, 5.06E+06) |
| Male | 35 to 39 | nasopharyngeal cancer | 3.09E-05 | 0.0000035 | beta(80.33, 2.60E+06) |
| Male | 40 to 44 | nasopharyngeal cancer | 4.64E-05 | 0.0000057 | beta(67.30, 1.45E+06) |
| Male | 45 to 49 | nasopharyngeal cancer | 7.29E-05 | 0.0000092 | beta(62.72, 8.60E+05) |
| Male | 50 to 54 | nasopharyngeal cancer | 1.00E-04 | 0.0000131 | beta(58.65, 5.86E+05) |
| Male | 55 to 59 | nasopharyngeal cancer | 1.31E-04 | 0.0000178 | beta(54.11, 4.13E+05) |
| Male | 60 to 64 | nasopharyngeal cancer | 1.23E-04 | 0.0000153 | beta(65.30, 5.29E+05) |
| Male | 65 to 69 | nasopharyngeal cancer | 1.19E-04 | 0.0000138 | beta(75.32, 6.31E+05) |
| Male | 70 to 74 | nasopharyngeal cancer | 1.09E-04 | 0.0000122 | beta(79.43, 7.31E+05) |
| Male | 75 to 79 | nasopharyngeal cancer | 7.74E-05 | 0.0000084 | beta(84.59, 1.09E+06) |
| Male | 80 to 84 | nasopharyngeal cancer | 5.36E-05 | 0.0000063 | beta(71.51, 1.33E+06) |
| Male | 85 to 89 | nasopharyngeal cancer | 4.39E-05 | 0.0000045 | beta(93.05, 2.12E+06) |
| Female | 15 to 19 | nasopharyngeal cancer | 5.82E-06 | 0.0000007 | beta(69.76, 1.20E+07) |
| Female | 20 to 24 | nasopharyngeal cancer | 7.75E-06 | 0.0000011 | beta(47.57, 6.14E+06) |
| Female | 25 to 29 | nasopharyngeal cancer | 8.35E-06 | 0.0000012 | beta(45.40, 5.44E+06) |
| Female | 30 to 34 | nasopharyngeal cancer | 1.12E-05 | 0.0000016 | beta(50.44, 4.52E+06) |
| Female | 35 to 39 | nasopharyngeal cancer | 1.67E-05 | 0.0000025 | beta(43.92, 2.63E+06) |
| Female | 40 to 44 | nasopharyngeal cancer | 2.60E-05 | 0.0000041 | beta(39.88, 1.53E+06) |
| Female | 45 to 49 | nasopharyngeal cancer | 3.44E-05 | 0.0000048 | beta(51.32, 1.49E+06) |
| Female | 50 to 54 | nasopharyngeal cancer | 5.64E-05 | 0.0000091 | beta(38.40, 6.81E+05) |
| Female | 55 to 59 | nasopharyngeal cancer | 5.94E-05 | 0.0000088 | beta(45.53, 7.66E+05) |
| Female | 60 to 64 | nasopharyngeal cancer | 5.97E-05 | 0.0000079 | beta(56.49, 9.47E+05) |
| Female | 65 to 69 | nasopharyngeal cancer | 5.76E-05 | 0.0000069 | beta(70.17, 1.22E+06) |
| Female | 70 to 74 | nasopharyngeal cancer | 5.64E-05 | 0.0000066 | beta(72.83, 1.29E+06) |
| Female | 75 to 79 | nasopharyngeal cancer | 4.53E-05 | 0.0000052 | beta(77.42, 1.71E+06) |
| Female | 80 to 84 | nasopharyngeal cancer | 3.25E-05 | 0.0000039 | beta(69.04, 2.12E+06) |
| Female | 85 to 89 | nasopharyngeal cancer | 3.22E-05 | 0.0000042 | beta(57.89, 1.80E+06) |
| Male | 15 to 19 | other pharyngeal cancer | 0.00E+00 | 0.0000000 |  |
| Male | 20 to 24 | other pharyngeal cancer | 4.44E-06 | 0.0000008 | beta(31.58, 7.11E+06) |
| Male | 25 to 29 | other pharyngeal cancer | 4.82E-06 | 0.0000007 | beta(52.96, 1.10E+07) |
| Male | 30 to 34 | other pharyngeal cancer | 9.28E-06 | 0.0000011 | beta(75.64, 8.15E+06) |
| Male | 35 to 39 | other pharyngeal cancer | 1.82E-05 | 0.0000022 | beta(68.45, 3.76E+06) |
| Male | 40 to 44 | other pharyngeal cancer | 3.60E-05 | 0.0000046 | beta(60.35, 1.68E+06) |
| Male | 45 to 49 | other pharyngeal cancer | 6.59E-05 | 0.0000087 | beta(57.57, 8.74E+05) |
| Male | 50 to 54 | other pharyngeal cancer | 1.07E-04 | 0.0000138 | beta(59.36, 5.57E+05) |
| Male | 55 to 59 | other pharyngeal cancer | 1.53E-04 | 0.0000226 | beta(45.89, 2.99E+05) |
| Male | 60 to 64 | other pharyngeal cancer | 1.89E-04 | 0.0000240 | beta(61.79, 3.27E+05) |
| Male | 65 to 69 | other pharyngeal cancer | 1.97E-04 | 0.0000238 | beta(68.80, 3.49E+05) |
| Male | 70 to 74 | other pharyngeal cancer | 2.04E-04 | 0.0000247 | beta(68.30, 3.35E+05) |
| Male | 75 to 79 | other pharyngeal cancer | 1.97E-04 | 0.0000227 | beta(75.46, 3.82E+05) |
| Male | 80 to 84 | other pharyngeal cancer | 2.22E-04 | 0.0000271 | beta(66.76, 3.01E+05) |
| Male | 85 to 89 | other pharyngeal cancer | 1.99E-04 | 0.0000212 | beta(88.43, 4.43E+05) |
| Female | 15 to 19 | other pharyngeal cancer | 0.00E+00 | 0.0000000 |  |
| Female | 20 to 24 | other pharyngeal cancer | 4.99E-06 | 0.0000009 | beta(32.91, 6.59E+06) |
| Female | 25 to 29 | other pharyngeal cancer | 5.92E-06 | 0.0000009 | beta(39.98, 6.76E+06) |
| Female | 30 to 34 | other pharyngeal cancer | 6.04E-06 | 0.0000009 | beta(47.81, 7.92E+06) |
| Female | 35 to 39 | other pharyngeal cancer | 1.06E-05 | 0.0000016 | beta(46.40, 4.39E+06) |
| Female | 40 to 44 | other pharyngeal cancer | 1.63E-05 | 0.0000024 | beta(46.30, 2.84E+06) |
| Female | 45 to 49 | other pharyngeal cancer | 2.50E-05 | 0.0000035 | beta(52.15, 2.08E+06) |
| Female | 50 to 54 | other pharyngeal cancer | 4.92E-05 | 0.0000082 | beta(36.29, 7.38E+05) |
| Female | 55 to 59 | other pharyngeal cancer | 5.51E-05 | 0.0000077 | beta(51.02, 9.27E+05) |
| Female | 60 to 64 | other pharyngeal cancer | 7.12E-05 | 0.0000095 | beta(56.73, 7.96E+05) |
| Female | 65 to 69 | other pharyngeal cancer | 6.99E-05 | 0.0000090 | beta(60.35, 8.64E+05) |
| Female | 70 to 74 | other pharyngeal cancer | 8.17E-05 | 0.0000105 | beta(60.12, 7.35E+05) |
| Female | 75 to 79 | other pharyngeal cancer | 7.05E-05 | 0.0000094 | beta(56.39, 8.00E+05) |
| Female | 80 to 84 | other pharyngeal cancer | 9.22E-05 | 0.0000138 | beta(44.58, 4.83E+05) |
| Female | 85 to 89 | other pharyngeal cancer | 9.00E-05 | 0.0000140 | beta(41.62, 4.62E+05) |
| Male | 15 to 19 | stroke | 1.71E-03 | 0.0002506 | beta(46.55, 2.72E+04) |
| Male | 20 to 24 | stroke | 2.24E-03 | 0.0002860 | beta(60.94, 2.72E+04) |
| Male | 25 to 29 | stroke | 2.91E-03 | 0.0003167 | beta(84.19, 2.88E+04) |
| Male | 30 to 34 | stroke | 3.94E-03 | 0.0003785 | beta(107.72, 2.73E+04) |
| Male | 35 to 39 | stroke | 5.43E-03 | 0.0004640 | beta(136.24, 2.49E+04) |
| Male | 40 to 44 | stroke | 7.66E-03 | 0.0006192 | beta(151.83, 1.97E+04) |
| Male | 45 to 49 | stroke | 1.09E-02 | 0.0008343 | beta(169.62, 1.54E+04) |
| Male | 50 to 54 | stroke | 1.53E-02 | 0.0013475 | beta(126.77, 8.16E+03) |
| Male | 55 to 59 | stroke | 2.07E-02 | 0.0016514 | beta(154.15, 7.29E+03) |
| Male | 60 to 64 | stroke | 2.63E-02 | 0.0022085 | beta(138.13, 5.11E+03) |
| Male | 65 to 69 | stroke | 2.98E-02 | 0.0028386 | beta(107.20, 3.48E+03) |
| Male | 70 to 74 | stroke | 3.08E-02 | 0.0037471 | beta(65.55, 2.06E+03) |
| Male | 75 to 79 | stroke | 2.99E-02 | 0.0039185 | beta(56.34, 1.83E+03) |
| Male | 80 to 84 | stroke | 2.72E-02 | 0.0040930 | beta(43.05, 1.54E+03) |
| Male | 85 to 89 | stroke | 2.03E-02 | 0.0030793 | beta(42.49, 2.05E+03) |
| Female | 15 to 19 | stroke | 2.19E-03 | 0.0002985 | beta(53.60, 2.44E+04) |
| Female | 20 to 24 | stroke | 2.86E-03 | 0.0003411 | beta(70.26, 2.45E+04) |
| Female | 25 to 29 | stroke | 3.63E-03 | 0.0003643 | beta(99.10, 2.72E+04) |
| Female | 30 to 34 | stroke | 4.66E-03 | 0.0004349 | beta(114.32, 2.44E+04) |
| Female | 35 to 39 | stroke | 6.30E-03 | 0.0005260 | beta(142.45, 2.25E+04) |
| Female | 40 to 44 | stroke | 8.45E-03 | 0.0006683 | beta(158.66, 1.86E+04) |
| Female | 45 to 49 | stroke | 1.16E-02 | 0.0008613 | beta(178.56, 1.52E+04) |
| Female | 50 to 54 | stroke | 1.56E-02 | 0.0012386 | beta(155.57, 9.84E+03) |
| Female | 55 to 59 | stroke | 2.01E-02 | 0.0014585 | beta(186.98, 9.09E+03) |
| Female | 60 to 64 | stroke | 2.46E-02 | 0.0018645 | beta(169.23, 6.72E+03) |
| Female | 65 to 69 | stroke | 2.70E-02 | 0.0022860 | beta(136.11, 4.90E+03) |
| Female | 70 to 74 | stroke | 2.79E-02 | 0.0029378 | beta(87.45, 3.05E+03) |
| Female | 75 to 79 | stroke | 2.71E-02 | 0.0031572 | beta(71.70, 2.57E+03) |
| Female | 80 to 84 | stroke | 2.45E-02 | 0.0031786 | beta(57.70, 2.30E+03) |
| Female | 85 to 89 | stroke | 1.85E-02 | 0.0025420 | beta(51.99, 2.76E+03) |
| **Pakistan** |  |  |  |  |  |
| Male | 15 to 19 | oesophageal cancer | 0.00E+00 | 0.0000000 |  |
| Male | 20 to 24 | oesophageal cancer | 2.85E-06 | 0.0000008 | beta(12.08, 4.24E+06) |
| Male | 25 to 29 | oesophageal cancer | 5.61E-06 | 0.0000014 | beta(15.77, 2.81E+06) |
| Male | 30 to 34 | oesophageal cancer | 1.19E-05 | 0.0000024 | beta(25.14, 2.12E+06) |
| Male | 35 to 39 | oesophageal cancer | 3.21E-05 | 0.0000067 | beta(22.72, 7.09E+05) |
| Male | 40 to 44 | oesophageal cancer | 7.52E-05 | 0.0000165 | beta(20.85, 2.77E+05) |
| Male | 45 to 49 | oesophageal cancer | 1.66E-04 | 0.0000367 | beta(20.58, 1.24E+05) |
| Male | 50 to 54 | oesophageal cancer | 3.00E-04 | 0.0000649 | beta(21.32, 7.11E+04) |
| Male | 55 to 59 | oesophageal cancer | 4.21E-04 | 0.0000907 | beta(21.54, 5.12E+04) |
| Male | 60 to 64 | oesophageal cancer | 5.16E-04 | 0.0001040 | beta(24.61, 4.77E+04) |
| Male | 65 to 69 | oesophageal cancer | 5.57E-04 | 0.0000998 | beta(31.14, 5.58E+04) |
| Male | 70 to 74 | oesophageal cancer | 5.19E-04 | 0.0000881 | beta(34.72, 6.68E+04) |
| Male | 75 to 79 | oesophageal cancer | 4.70E-04 | 0.0000847 | beta(30.81, 6.55E+04) |
| Male | 80 to 84 | oesophageal cancer | 3.98E-04 | 0.0000673 | beta(34.92, 8.78E+04) |
| Male | 85 to 89 | oesophageal cancer | 4.04E-04 | 0.0000652 | beta(38.38, 9.49E+04) |
| Female | 15 to 19 | oesophageal cancer | 0.00E+00 | 0.0000000 |  |
| Female | 20 to 24 | oesophageal cancer | 6.09E-06 | 0.0000016 | beta(15.03, 2.47E+06) |
| Female | 25 to 29 | oesophageal cancer | 1.20E-05 | 0.0000031 | beta(14.81, 1.24E+06) |
| Female | 30 to 34 | oesophageal cancer | 1.71E-05 | 0.0000046 | beta(13.73, 8.05E+05) |
| Female | 35 to 39 | oesophageal cancer | 3.28E-05 | 0.0000086 | beta(14.39, 4.39E+05) |
| Female | 40 to 44 | oesophageal cancer | 8.14E-05 | 0.0000199 | beta(16.74, 2.06E+05) |
| Female | 45 to 49 | oesophageal cancer | 1.46E-04 | 0.0000343 | beta(18.23, 1.25E+05) |
| Female | 50 to 54 | oesophageal cancer | 2.46E-04 | 0.0000500 | beta(24.14, 9.82E+04) |
| Female | 55 to 59 | oesophageal cancer | 3.74E-04 | 0.0000812 | beta(21.20, 5.67E+04) |
| Female | 60 to 64 | oesophageal cancer | 4.24E-04 | 0.0000854 | beta(24.62, 5.81E+04) |
| Female | 65 to 69 | oesophageal cancer | 5.03E-04 | 0.0000964 | beta(27.25, 5.41E+04) |
| Female | 70 to 74 | oesophageal cancer | 5.34E-04 | 0.0000965 | beta(30.55, 5.72E+04) |
| Female | 75 to 79 | oesophageal cancer | 5.15E-04 | 0.0000943 | beta(29.74, 5.78E+04) |
| Female | 80 to 84 | oesophageal cancer | 4.73E-04 | 0.0000991 | beta(22.79, 4.82E+04) |
| Female | 85 to 89 | oesophageal cancer | 5.08E-04 | 0.0000996 | beta(25.95, 5.11E+04) |
| Male | 15 to 19 | oral cancer | 8.11E-05 | 0.0000247 | beta(10.75, 1.33E+05) |
| Male | 20 to 24 | oral cancer | 1.14E-04 | 0.0000313 | beta(13.32, 1.17E+05) |
| Male | 25 to 29 | oral cancer | 1.95E-04 | 0.0000529 | beta(13.54, 6.96E+04) |
| Male | 30 to 34 | oral cancer | 3.29E-04 | 0.0000846 | beta(15.14, 4.59E+04) |
| Male | 35 to 39 | oral cancer | 6.50E-04 | 0.0001671 | beta(15.11, 2.32E+04) |
| Male | 40 to 44 | oral cancer | 9.32E-04 | 0.0002511 | beta(13.77, 1.48E+04) |
| Male | 45 to 49 | oral cancer | 1.23E-03 | 0.0003085 | beta(15.92, 1.29E+04) |
| Male | 50 to 54 | oral cancer | 1.66E-03 | 0.0004296 | beta(14.95, 8.98E+03) |
| Male | 55 to 59 | oral cancer | 1.92E-03 | 0.0004857 | beta(15.66, 8.12E+03) |
| Male | 60 to 64 | oral cancer | 2.21E-03 | 0.0005133 | beta(18.51, 8.35E+03) |
| Male | 65 to 69 | oral cancer | 2.29E-03 | 0.0004866 | beta(22.05, 9.62E+03) |
| Male | 70 to 74 | oral cancer | 2.08E-03 | 0.0004695 | beta(19.49, 9.37E+03) |
| Male | 75 to 79 | oral cancer | 1.78E-03 | 0.0003801 | beta(21.96, 1.23E+04) |
| Male | 80 to 84 | oral cancer | 1.08E-03 | 0.0002171 | beta(24.58, 2.28E+04) |
| Male | 85 to 89 | oral cancer | 8.87E-04 | 0.0001673 | beta(28.04, 3.16E+04) |
| Female | 15 to 19 | oral cancer | 9.11E-05 | 0.0000219 | beta(17.36, 1.91E+05) |
| Female | 20 to 24 | oral cancer | 2.03E-04 | 0.0000487 | beta(17.38, 8.55E+04) |
| Female | 25 to 29 | oral cancer | 3.04E-04 | 0.0000743 | beta(16.78, 5.51E+04) |
| Female | 30 to 34 | oral cancer | 4.69E-04 | 0.0001250 | beta(14.09, 3.00E+04) |
| Female | 35 to 39 | oral cancer | 6.37E-04 | 0.0001684 | beta(14.28, 2.24E+04) |
| Female | 40 to 44 | oral cancer | 8.24E-04 | 0.0002131 | beta(14.94, 1.81E+04) |
| Female | 45 to 49 | oral cancer | 1.10E-03 | 0.0002595 | beta(18.02, 1.63E+04) |
| Female | 50 to 54 | oral cancer | 1.55E-03 | 0.0003717 | beta(17.36, 1.12E+04) |
| Female | 55 to 59 | oral cancer | 1.88E-03 | 0.0004308 | beta(18.93, 1.01E+04) |
| Female | 60 to 64 | oral cancer | 2.19E-03 | 0.0004874 | beta(20.23, 9.20E+03) |
| Female | 65 to 69 | oral cancer | 2.24E-03 | 0.0004867 | beta(21.21, 9.43E+03) |
| Female | 70 to 74 | oral cancer | 2.11E-03 | 0.0004545 | beta(21.60, 1.02E+04) |
| Female | 75 to 79 | oral cancer | 1.89E-03 | 0.0004005 | beta(22.18, 1.17E+04) |
| Female | 80 to 84 | oral cancer | 1.22E-03 | 0.0002785 | beta(19.06, 1.56E+04) |
| Female | 85 to 89 | oral cancer | 1.04E-03 | 0.0002019 | beta(26.40, 2.54E+04) |
| Male | 15 to 19 | nasopharyngeal cancer | 1.23E-05 | 0.0000036 | beta(11.78, 9.55E+05) |
| Male | 20 to 24 | nasopharyngeal cancer | 1.36E-05 | 0.0000038 | beta(12.84, 9.45E+05) |
| Male | 25 to 29 | nasopharyngeal cancer | 1.93E-05 | 0.0000050 | beta(14.56, 7.56E+05) |
| Male | 30 to 34 | nasopharyngeal cancer | 2.89E-05 | 0.0000065 | beta(19.80, 6.85E+05) |
| Male | 35 to 39 | nasopharyngeal cancer | 4.83E-05 | 0.0000113 | beta(18.36, 3.80E+05) |
| Male | 40 to 44 | nasopharyngeal cancer | 8.13E-05 | 0.0000181 | beta(20.13, 2.47E+05) |
| Male | 45 to 49 | nasopharyngeal cancer | 1.19E-04 | 0.0000274 | beta(18.94, 1.59E+05) |
| Male | 50 to 54 | nasopharyngeal cancer | 1.63E-04 | 0.0000370 | beta(19.48, 1.19E+05) |
| Male | 55 to 59 | nasopharyngeal cancer | 1.95E-04 | 0.0000434 | beta(20.24, 1.04E+05) |
| Male | 60 to 64 | nasopharyngeal cancer | 1.87E-04 | 0.0000384 | beta(23.72, 1.27E+05) |
| Male | 65 to 69 | nasopharyngeal cancer | 1.69E-04 | 0.0000330 | beta(26.35, 1.56E+05) |
| Male | 70 to 74 | nasopharyngeal cancer | 1.54E-04 | 0.0000303 | beta(25.72, 1.67E+05) |
| Male | 75 to 79 | nasopharyngeal cancer | 1.03E-04 | 0.0000224 | beta(20.95, 2.04E+05) |
| Male | 80 to 84 | nasopharyngeal cancer | 6.65E-05 | 0.0000147 | beta(20.36, 3.06E+05) |
| Male | 85 to 89 | nasopharyngeal cancer | 5.16E-05 | 0.0000122 | beta(17.99, 3.49E+05) |
| Female | 15 to 19 | nasopharyngeal cancer | 5.94E-06 | 0.0000015 | beta(15.16, 2.55E+06) |
| Female | 20 to 24 | nasopharyngeal cancer | 8.42E-06 | 0.0000026 | beta(10.88, 1.29E+06) |
| Female | 25 to 29 | nasopharyngeal cancer | 1.01E-05 | 0.0000025 | beta(16.57, 1.64E+06) |
| Female | 30 to 34 | nasopharyngeal cancer | 1.42E-05 | 0.0000037 | beta(14.40, 1.02E+06) |
| Female | 35 to 39 | nasopharyngeal cancer | 2.18E-05 | 0.0000059 | beta(13.66, 6.27E+05) |
| Female | 40 to 44 | nasopharyngeal cancer | 3.18E-05 | 0.0000081 | beta(15.47, 4.87E+05) |
| Female | 45 to 49 | nasopharyngeal cancer | 4.22E-05 | 0.0000094 | beta(20.11, 4.76E+05) |
| Female | 50 to 54 | nasopharyngeal cancer | 5.63E-05 | 0.0000125 | beta(20.41, 3.63E+05) |
| Female | 55 to 59 | nasopharyngeal cancer | 6.57E-05 | 0.0000142 | beta(21.40, 3.26E+05) |
| Female | 60 to 64 | nasopharyngeal cancer | 6.44E-05 | 0.0000134 | beta(23.24, 3.61E+05) |
| Female | 65 to 69 | nasopharyngeal cancer | 5.88E-05 | 0.0000115 | beta(26.01, 4.42E+05) |
| Female | 70 to 74 | nasopharyngeal cancer | 5.92E-05 | 0.0000115 | beta(26.31, 4.44E+05) |
| Female | 75 to 79 | nasopharyngeal cancer | 4.41E-05 | 0.0000096 | beta(21.04, 4.77E+05) |
| Female | 80 to 84 | nasopharyngeal cancer | 2.82E-05 | 0.0000064 | beta(19.16, 6.79E+05) |
| Female | 85 to 89 | nasopharyngeal cancer | 2.61E-05 | 0.0000059 | beta(19.71, 7.55E+05) |
| Male | 15 to 19 | other pharyngeal cancer | 0.00E+00 | 0.0000000 |  |
| Male | 20 to 24 | other pharyngeal cancer | 1.51E-06 | 0.0000004 | beta(14.40, 9.56E+06) |
| Male | 25 to 29 | other pharyngeal cancer | 2.19E-06 | 0.0000005 | beta(17.75, 8.12E+06) |
| Male | 30 to 34 | other pharyngeal cancer | 4.34E-06 | 0.0000010 | beta(20.34, 4.69E+06) |
| Male | 35 to 39 | other pharyngeal cancer | 1.12E-05 | 0.0000024 | beta(21.21, 1.90E+06) |
| Male | 40 to 44 | other pharyngeal cancer | 2.99E-05 | 0.0000069 | beta(18.92, 6.34E+05) |
| Male | 45 to 49 | other pharyngeal cancer | 5.79E-05 | 0.0000132 | beta(19.34, 3.34E+05) |
| Male | 50 to 54 | other pharyngeal cancer | 1.01E-04 | 0.0000225 | beta(20.01, 1.99E+05) |
| Male | 55 to 59 | other pharyngeal cancer | 1.38E-04 | 0.0000333 | beta(17.17, 1.25E+05) |
| Male | 60 to 64 | other pharyngeal cancer | 1.63E-04 | 0.0000380 | beta(18.45, 1.13E+05) |
| Male | 65 to 69 | other pharyngeal cancer | 1.57E-04 | 0.0000342 | beta(21.02, 1.34E+05) |
| Male | 70 to 74 | other pharyngeal cancer | 1.38E-04 | 0.0000276 | beta(25.01, 1.81E+05) |
| Male | 75 to 79 | other pharyngeal cancer | 1.05E-04 | 0.0000211 | beta(24.78, 2.35E+05) |
| Male | 80 to 84 | other pharyngeal cancer | 8.74E-05 | 0.0000189 | beta(21.27, 2.43E+05) |
| Male | 85 to 89 | other pharyngeal cancer | 7.41E-05 | 0.0000139 | beta(28.32, 3.82E+05) |
| Female | 15 to 19 | other pharyngeal cancer | 0.00E+00 | 0.0000000 |  |
| Female | 20 to 24 | other pharyngeal cancer | 2.37E-06 | 0.0000007 | beta(10.19, 4.30E+06) |
| Female | 25 to 29 | other pharyngeal cancer | 3.62E-06 | 0.0000010 | beta(13.10, 3.62E+06) |
| Female | 30 to 34 | other pharyngeal cancer | 4.72E-06 | 0.0000013 | beta(13.59, 2.88E+06) |
| Female | 35 to 39 | other pharyngeal cancer | 8.33E-06 | 0.0000022 | beta(13.98, 1.68E+06) |
| Female | 40 to 44 | other pharyngeal cancer | 1.20E-05 | 0.0000032 | beta(14.39, 1.20E+06) |
| Female | 45 to 49 | other pharyngeal cancer | 2.02E-05 | 0.0000053 | beta(14.33, 7.10E+05) |
| Female | 50 to 54 | other pharyngeal cancer | 3.35E-05 | 0.0000075 | beta(20.10, 6.01E+05) |
| Female | 55 to 59 | other pharyngeal cancer | 4.12E-05 | 0.0000084 | beta(23.88, 5.80E+05) |
| Female | 60 to 64 | other pharyngeal cancer | 5.15E-05 | 0.0000097 | beta(28.27, 5.49E+05) |
| Female | 65 to 69 | other pharyngeal cancer | 4.88E-05 | 0.0000092 | beta(27.90, 5.72E+05) |
| Female | 70 to 74 | other pharyngeal cancer | 5.01E-05 | 0.0000089 | beta(31.91, 6.37E+05) |
| Female | 75 to 79 | other pharyngeal cancer | 3.82E-05 | 0.0000075 | beta(25.66, 6.72E+05) |
| Female | 80 to 84 | other pharyngeal cancer | 3.77E-05 | 0.0000087 | beta(18.91, 5.02E+05) |
| Female | 85 to 89 | other pharyngeal cancer | 3.60E-05 | 0.0000074 | beta(23.88, 6.63E+05) |
| Male | 15 to 19 | stroke | 1.95E-03 | 0.0003166 | beta(38.01, 1.94E+04) |
| Male | 20 to 24 | stroke | 2.51E-03 | 0.0003752 | beta(44.66, 1.77E+04) |
| Male | 25 to 29 | stroke | 3.44E-03 | 0.0004315 | beta(63.47, 1.84E+04) |
| Male | 30 to 34 | stroke | 4.67E-03 | 0.0005083 | beta(84.09, 1.79E+04) |
| Male | 35 to 39 | stroke | 6.78E-03 | 0.0006333 | beta(113.78, 1.67E+04) |
| Male | 40 to 44 | stroke | 9.67E-03 | 0.0008822 | beta(119.03, 1.22E+04) |
| Male | 45 to 49 | stroke | 1.42E-02 | 0.0012149 | beta(134.07, 9.33E+03) |
| Male | 50 to 54 | stroke | 2.00E-02 | 0.0018832 | beta(110.67, 5.42E+03) |
| Male | 55 to 59 | stroke | 2.70E-02 | 0.0023219 | beta(131.94, 4.75E+03) |
| Male | 60 to 64 | stroke | 3.46E-02 | 0.0030561 | beta(123.65, 3.45E+03) |
| Male | 65 to 69 | stroke | 3.97E-02 | 0.0038954 | beta(99.51, 2.41E+03) |
| Male | 70 to 74 | stroke | 4.14E-02 | 0.0050513 | beta(64.22, 1.49E+03) |
| Male | 75 to 79 | stroke | 4.02E-02 | 0.0056329 | beta(48.78, 1.17E+03) |
| Male | 80 to 84 | stroke | 3.59E-02 | 0.0056982 | beta(38.18, 1.03E+03) |
| Male | 85 to 89 | stroke | 2.57E-02 | 0.0035219 | beta(51.84, 1.97E+03) |
| Female | 15 to 19 | stroke | 2.84E-03 | 0.0003982 | beta(50.87, 1.78E+04) |
| Female | 20 to 24 | stroke | 3.74E-03 | 0.0004980 | beta(56.14, 1.50E+04) |
| Female | 25 to 29 | stroke | 5.02E-03 | 0.0005344 | beta(87.66, 1.74E+04) |
| Female | 30 to 34 | stroke | 6.76E-03 | 0.0006988 | beta(93.05, 1.37E+04) |
| Female | 35 to 39 | stroke | 9.32E-03 | 0.0008708 | beta(113.55, 1.21E+04) |
| Female | 40 to 44 | stroke | 1.31E-02 | 0.0011044 | beta(139.47, 1.05E+04) |
| Female | 45 to 49 | stroke | 1.84E-02 | 0.0015576 | beta(136.53, 7.30E+03) |
| Female | 50 to 54 | stroke | 2.53E-02 | 0.0021793 | beta(131.39, 5.06E+03) |
| Female | 55 to 59 | stroke | 3.35E-02 | 0.0027162 | beta(146.83, 4.24E+03) |
| Female | 60 to 64 | stroke | 4.21E-02 | 0.0034636 | beta(141.47, 3.22E+03) |
| Female | 65 to 69 | stroke | 4.84E-02 | 0.0043409 | beta(118.26, 2.33E+03) |
| Female | 70 to 74 | stroke | 5.16E-02 | 0.0055197 | beta(82.69, 1.52E+03) |
| Female | 75 to 79 | stroke | 5.02E-02 | 0.0059623 | beta(67.21, 1.27E+03) |
| Female | 80 to 84 | stroke | 4.54E-02 | 0.0063006 | beta(49.61, 1.04E+03) |
| Female | 85 to 89 | stroke | 3.76E-02 | 0.0048539 | beta(57.74, 1.48E+03) |
| **Bangladesh** |  |  |  |  |  |
| Male | 15 to 19 | oesophageal cancer | 0 | 0.0000000 |  |
| Male | 20 to 24 | oesophageal cancer | 2.10E-06 | 0.0000009 | beta(5.61, 2.67E+06) |
| Male | 25 to 29 | oesophageal cancer | 4.16E-06 | 0.0000022 | beta(3.50, 8.40E+05) |
| Male | 30 to 34 | oesophageal cancer | 7.04E-06 | 0.0000033 | beta(4.50, 6.39E+05) |
| Male | 35 to 39 | oesophageal cancer | 1.50E-05 | 0.0000066 | beta(5.19, 3.47E+05) |
| Male | 40 to 44 | oesophageal cancer | 3.12E-05 | 0.0000136 | beta(5.25, 1.68E+05) |
| Male | 45 to 49 | oesophageal cancer | 8.33E-05 | 0.0000270 | beta(9.51, 1.14E+05) |
| Male | 50 to 54 | oesophageal cancer | 0.000173648 | 0.0000494 | beta(12.37, 7.12E+04) |
| Male | 55 to 59 | oesophageal cancer | 0.000242913 | 0.0000655 | beta(13.76, 5.66E+04) |
| Male | 60 to 64 | oesophageal cancer | 0.000303074 | 0.0000768 | beta(15.58, 5.14E+04) |
| Male | 65 to 69 | oesophageal cancer | 0.000330396 | 0.0000831 | beta(15.79, 4.78E+04) |
| Male | 70 to 74 | oesophageal cancer | 0.000298556 | 0.0000831 | beta(12.91, 4.32E+04) |
| Male | 75 to 79 | oesophageal cancer | 0.000294274 | 0.0000887 | beta(11.00, 3.74E+04) |
| Male | 80 to 84 | oesophageal cancer | 0.000291259 | 0.0000738 | beta(15.58, 5.35E+04) |
| Male | 85 to 89 | oesophageal cancer | 0.000331486 | 0.0000764 | beta(18.80, 5.67E+04) |
| Female | 15 to 19 | oesophageal cancer | 0 | 0.0000000 |  |
| Female | 20 to 24 | oesophageal cancer | 2.67E-06 | 0.0000013 | beta(4.08, 1.52E+06) |
| Female | 25 to 29 | oesophageal cancer | 4.85E-06 | 0.0000024 | beta(3.95, 8.14E+05) |
| Female | 30 to 34 | oesophageal cancer | 5.39E-06 | 0.0000027 | beta(3.97, 7.37E+05) |
| Female | 35 to 39 | oesophageal cancer | 1.03E-05 | 0.0000044 | beta(5.48, 5.34E+05) |
| Female | 40 to 44 | oesophageal cancer | 2.86E-05 | 0.0000108 | beta(7.09, 2.48E+05) |
| Female | 45 to 49 | oesophageal cancer | 5.66E-05 | 0.0000197 | beta(8.27, 1.46E+05) |
| Female | 50 to 54 | oesophageal cancer | 0.000116066 | 0.0000317 | beta(13.41, 1.16E+05) |
| Female | 55 to 59 | oesophageal cancer | 0.000145357 | 0.0000461 | beta(9.92, 6.83E+04) |
| Female | 60 to 64 | oesophageal cancer | 0.000146578 | 0.0000492 | beta(8.87, 6.05E+04) |
| Female | 65 to 69 | oesophageal cancer | 0.000169516 | 0.0000612 | beta(7.68, 4.53E+04) |
| Female | 70 to 74 | oesophageal cancer | 0.000209901 | 0.0000701 | beta(8.97, 4.27E+04) |
| Female | 75 to 79 | oesophageal cancer | 0.000208588 | 0.0000776 | beta(7.22, 3.46E+04) |
| Female | 80 to 84 | oesophageal cancer | 0.000205488 | 0.0000717 | beta(8.20, 3.99E+04) |
| Female | 85 to 89 | oesophageal cancer | 0.000235588 | 0.0000752 | beta(9.82, 4.17E+04) |
| Male | 15 to 19 | oral cancer | 2.51E-05 | 0.0000111 | beta(5.11, 2.03E+05) |
| Male | 20 to 24 | oral cancer | 2.74E-05 | 0.0000128 | beta(4.57, 1.67E+05) |
| Male | 25 to 29 | oral cancer | 4.57E-05 | 0.0000198 | beta(5.30, 1.16E+05) |
| Male | 30 to 34 | oral cancer | 7.09E-05 | 0.0000359 | beta(3.90, 5.50E+04) |
| Male | 35 to 39 | oral cancer | 0.000130523 | 0.0000698 | beta(3.49, 2.67E+04) |
| Male | 40 to 44 | oral cancer | 0.000178905 | 0.0000867 | beta(4.26, 2.38E+04) |
| Male | 45 to 49 | oral cancer | 0.000306689 | 0.0001166 | beta(6.92, 2.26E+04) |
| Male | 50 to 54 | oral cancer | 0.000554695 | 0.0001686 | beta(10.82, 1.95E+04) |
| Male | 55 to 59 | oral cancer | 0.000677668 | 0.0002072 | beta(10.69, 1.58E+04) |
| Male | 60 to 64 | oral cancer | 0.000833339 | 0.0002687 | beta(9.61, 1.15E+04) |
| Male | 65 to 69 | oral cancer | 0.000884998 | 0.0002585 | beta(11.71, 1.32E+04) |
| Male | 70 to 74 | oral cancer | 0.000800707 | 0.0002605 | beta(9.44, 1.18E+04) |
| Male | 75 to 79 | oral cancer | 0.000781317 | 0.0002526 | beta(9.56, 1.22E+04) |
| Male | 80 to 84 | oral cancer | 0.000544818 | 0.0001552 | beta(12.32, 2.26E+04) |
| Male | 85 to 89 | oral cancer | 0.000454909 | 0.0001210 | beta(14.14, 3.11E+04) |
| Female | 15 to 19 | oral cancer | 2.63E-05 | 0.0000101 | beta(6.73, 2.56E+05) |
| Female | 20 to 24 | oral cancer | 4.14E-05 | 0.0000168 | beta(6.05, 1.46E+05) |
| Female | 25 to 29 | oral cancer | 5.09E-05 | 0.0000216 | beta(5.53, 1.09E+05) |
| Female | 30 to 34 | oral cancer | 7.66E-05 | 0.0000302 | beta(6.43, 8.39E+04) |
| Female | 35 to 39 | oral cancer | 0.000114253 | 0.0000394 | beta(8.42, 7.37E+04) |
| Female | 40 to 44 | oral cancer | 0.000162733 | 0.0000501 | beta(10.54, 6.47E+04) |
| Female | 45 to 49 | oral cancer | 0.000235337 | 0.0000666 | beta(12.48, 5.30E+04) |
| Female | 50 to 54 | oral cancer | 0.000384175 | 0.0001068 | beta(12.94, 3.37E+04) |
| Female | 55 to 59 | oral cancer | 0.000469849 | 0.0001300 | beta(13.05, 2.78E+04) |
| Female | 60 to 64 | oral cancer | 0.000554558 | 0.0001661 | beta(11.14, 2.01E+04) |
| Female | 65 to 69 | oral cancer | 0.000622485 | 0.0001897 | beta(10.77, 1.73E+04) |
| Female | 70 to 74 | oral cancer | 0.000665667 | 0.0002078 | beta(10.26, 1.54E+04) |
| Female | 75 to 79 | oral cancer | 0.000689391 | 0.0002298 | beta(9.00, 1.30E+04) |
| Female | 80 to 84 | oral cancer | 0.00050026 | 0.0001505 | beta(11.04, 2.21E+04) |
| Female | 85 to 89 | oral cancer | 0.000440612 | 0.0001296 | beta(11.55, 2.62E+04) |
| Male | 15 to 19 | nasopharyngeal cancer | 7.36E-06 | 0.0000032 | beta(5.17, 7.03E+05) |
| Male | 20 to 24 | nasopharyngeal cancer | 6.64E-06 | 0.0000031 | beta(4.52, 6.80E+05) |
| Male | 25 to 29 | nasopharyngeal cancer | 1.08E-05 | 0.0000052 | beta(4.32, 4.02E+05) |
| Male | 30 to 34 | nasopharyngeal cancer | 1.54E-05 | 0.0000066 | beta(5.47, 3.56E+05) |
| Male | 35 to 39 | nasopharyngeal cancer | 2.39E-05 | 0.0000100 | beta(5.68, 2.38E+05) |
| Male | 40 to 44 | nasopharyngeal cancer | 3.73E-05 | 0.0000141 | beta(6.97, 1.87E+05) |
| Male | 45 to 49 | nasopharyngeal cancer | 6.55E-05 | 0.0000228 | beta(8.28, 1.26E+05) |
| Male | 50 to 54 | nasopharyngeal cancer | 9.85E-05 | 0.0000318 | beta(9.59, 9.73E+04) |
| Male | 55 to 59 | nasopharyngeal cancer | 0.000120515 | 0.0000397 | beta(9.21, 7.64E+04) |
| Male | 60 to 64 | nasopharyngeal cancer | 0.000122503 | 0.0000374 | beta(10.71, 8.75E+04) |
| Male | 65 to 69 | nasopharyngeal cancer | 0.000116155 | 0.0000345 | beta(11.36, 9.77E+04) |
| Male | 70 to 74 | nasopharyngeal cancer | 9.87E-05 | 0.0000308 | beta(10.24, 1.04E+05) |
| Male | 75 to 79 | nasopharyngeal cancer | 7.11E-05 | 0.0000232 | beta(9.43, 1.33E+05) |
| Male | 80 to 84 | nasopharyngeal cancer | 5.60E-05 | 0.0000176 | beta(10.16, 1.81E+05) |
| Male | 85 to 89 | nasopharyngeal cancer | 5.20E-05 | 0.0000183 | beta(8.06, 1.55E+05) |
| Female | 15 to 19 | nasopharyngeal cancer | 6.96E-06 | 0.0000026 | beta(7.16, 1.03E+06) |
| Female | 20 to 24 | nasopharyngeal cancer | 7.03E-06 | 0.0000034 | beta(4.35, 6.19E+05) |
| Female | 25 to 29 | nasopharyngeal cancer | 7.94E-06 | 0.0000030 | beta(6.90, 8.69E+05) |
| Female | 30 to 34 | nasopharyngeal cancer | 9.50E-06 | 0.0000038 | beta(6.31, 6.64E+05) |
| Female | 35 to 39 | nasopharyngeal cancer | 1.54E-05 | 0.0000055 | beta(7.91, 5.14E+05) |
| Female | 40 to 44 | nasopharyngeal cancer | 2.44E-05 | 0.0000085 | beta(8.29, 3.39E+05) |
| Female | 45 to 49 | nasopharyngeal cancer | 3.39E-05 | 0.0000098 | beta(11.91, 3.52E+05) |
| Female | 50 to 54 | nasopharyngeal cancer | 5.83E-05 | 0.0000164 | beta(12.57, 2.16E+05) |
| Female | 55 to 59 | nasopharyngeal cancer | 5.95E-05 | 0.0000165 | beta(12.95, 2.18E+05) |
| Female | 60 to 64 | nasopharyngeal cancer | 5.62E-05 | 0.0000176 | beta(10.23, 1.82E+05) |
| Female | 65 to 69 | nasopharyngeal cancer | 5.65E-05 | 0.0000180 | beta(9.82, 1.74E+05) |
| Female | 70 to 74 | nasopharyngeal cancer | 5.53E-05 | 0.0000162 | beta(11.67, 2.11E+05) |
| Female | 75 to 79 | nasopharyngeal cancer | 4.35E-05 | 0.0000139 | beta(9.87, 2.27E+05) |
| Female | 80 to 84 | nasopharyngeal cancer | 3.42E-05 | 0.0000108 | beta(9.97, 2.91E+05) |
| Female | 85 to 89 | nasopharyngeal cancer | 4.06E-05 | 0.0000140 | beta(8.41, 2.07E+05) |
| Male | 15 to 19 | other pharyngeal cancer | 0 | 0.0000000 |  |
| Male | 20 to 24 | other pharyngeal cancer | 1.96E-06 | 0.0000009 | beta(4.48, 2.28E+06) |
| Male | 25 to 29 | other pharyngeal cancer | 2.81E-06 | 0.0000014 | beta(4.15, 1.48E+06) |
| Male | 30 to 34 | other pharyngeal cancer | 4.88E-06 | 0.0000022 | beta(4.85, 9.95E+05) |
| Male | 35 to 39 | other pharyngeal cancer | 9.67E-06 | 0.0000046 | beta(4.39, 4.54E+05) |
| Male | 40 to 44 | other pharyngeal cancer | 2.25E-05 | 0.0000094 | beta(5.65, 2.52E+05) |
| Male | 45 to 49 | other pharyngeal cancer | 4.84E-05 | 0.0000179 | beta(7.33, 1.51E+05) |
| Male | 50 to 54 | other pharyngeal cancer | 8.88E-05 | 0.0000263 | beta(11.43, 1.29E+05) |
| Male | 55 to 59 | other pharyngeal cancer | 0.00011449 | 0.0000366 | beta(9.78, 8.54E+04) |
| Male | 60 to 64 | other pharyngeal cancer | 0.000146572 | 0.0000466 | beta(9.90, 6.76E+04) |
| Male | 65 to 69 | other pharyngeal cancer | 0.000150836 | 0.0000507 | beta(8.87, 5.88E+04) |
| Male | 70 to 74 | other pharyngeal cancer | 0.000137973 | 0.0000435 | beta(10.05, 7.28E+04) |
| Male | 75 to 79 | other pharyngeal cancer | 0.000121159 | 0.0000373 | beta(10.54, 8.69E+04) |
| Male | 80 to 84 | other pharyngeal cancer | 0.000130766 | 0.0000364 | beta(12.89, 9.85E+04) |
| Male | 85 to 89 | other pharyngeal cancer | 0.000129976 | 0.0000367 | beta(12.56, 9.66E+04) |
| Female | 15 to 19 | other pharyngeal cancer | 0 | 0.0000000 |  |
| Female | 20 to 24 | other pharyngeal cancer | 2.36E-06 | 0.0000012 | beta(3.92, 1.66E+06) |
| Female | 25 to 29 | other pharyngeal cancer | 3.33E-06 | 0.0000017 | beta(4.07, 1.22E+06) |
| Female | 30 to 34 | other pharyngeal cancer | 3.43E-06 | 0.0000015 | beta(4.95, 1.44E+06) |
| Female | 35 to 39 | other pharyngeal cancer | 6.23E-06 | 0.0000025 | beta(6.18, 9.93E+05) |
| Female | 40 to 44 | other pharyngeal cancer | 9.69E-06 | 0.0000033 | beta(8.66, 8.94E+05) |
| Female | 45 to 49 | other pharyngeal cancer | 1.57E-05 | 0.0000047 | beta(10.97, 7.01E+05) |
| Female | 50 to 54 | other pharyngeal cancer | 3.35E-05 | 0.0000093 | beta(12.89, 3.84E+05) |
| Female | 55 to 59 | other pharyngeal cancer | 3.59E-05 | 0.0000098 | beta(13.54, 3.77E+05) |
| Female | 60 to 64 | other pharyngeal cancer | 4.37E-05 | 0.0000116 | beta(14.15, 3.24E+05) |
| Female | 65 to 69 | other pharyngeal cancer | 4.22E-05 | 0.0000121 | beta(12.11, 2.87E+05) |
| Female | 70 to 74 | other pharyngeal cancer | 4.54E-05 | 0.0000133 | beta(11.69, 2.57E+05) |
| Female | 75 to 79 | other pharyngeal cancer | 3.69E-05 | 0.0000108 | beta(11.64, 3.15E+05) |
| Female | 80 to 84 | other pharyngeal cancer | 4.64E-05 | 0.0000149 | beta(9.73, 2.10E+05) |
| Female | 85 to 89 | other pharyngeal cancer | 5.42E-05 | 0.0000147 | beta(13.61, 2.51E+05) |
| Male | 15 to 19 | stroke | 0.001393669 | 0.0002968 | beta(22.01, 1.58E+04) |
| Male | 20 to 24 | stroke | 0.001821937 | 0.0003338 | beta(29.74, 1.63E+04) |
| Male | 25 to 29 | stroke | 0.002840366 | 0.0004396 | beta(41.63, 1.46E+04) |
| Male | 30 to 34 | stroke | 0.004190878 | 0.0005616 | beta(55.45, 1.32E+04) |
| Male | 35 to 39 | stroke | 0.006077287 | 0.0006679 | beta(82.28, 1.35E+04) |
| Male | 40 to 44 | stroke | 0.008715106 | 0.0008717 | beta(99.07, 1.13E+04) |
| Male | 45 to 49 | stroke | 0.012433831 | 0.0010151 | beta(148.15, 1.18E+04) |
| Male | 50 to 54 | stroke | 0.017283 | 0.0013317 | beta(165.50, 9.41E+03) |
| Male | 55 to 59 | stroke | 0.022992802 | 0.0015214 | beta(223.14, 9.48E+03) |
| Male | 60 to 64 | stroke | 0.028956249 | 0.0019039 | beta(224.59, 7.53E+03) |
| Male | 65 to 69 | stroke | 0.032114863 | 0.0023362 | beta(182.86, 5.51E+03) |
| Male | 70 to 74 | stroke | 0.03187347 | 0.0031630 | beta(98.28, 2.99E+03) |
| Male | 75 to 79 | stroke | 0.0294364 | 0.0030919 | beta(87.94, 2.90E+03) |
| Male | 80 to 84 | stroke | 0.02498142 | 0.0033312 | beta(54.81, 2.14E+03) |
| Male | 85 to 89 | stroke | 0.016792888 | 0.0023594 | beta(49.79, 2.92E+03) |
| Female | 15 to 19 | stroke | 0.002580649 | 0.0004338 | beta(35.30, 1.36E+04) |
| Female | 20 to 24 | stroke | 0.003325255 | 0.0005514 | beta(36.24, 1.09E+04) |
| Female | 25 to 29 | stroke | 0.004365076 | 0.0005804 | beta(56.30, 1.28E+04) |
| Female | 30 to 34 | stroke | 0.005941062 | 0.0007052 | beta(70.55, 1.18E+04) |
| Female | 35 to 39 | stroke | 0.008086791 | 0.0008953 | beta(80.91, 9.92E+03) |
| Female | 40 to 44 | stroke | 0.011359585 | 0.0010882 | beta(107.72, 9.37E+03) |
| Female | 45 to 49 | stroke | 0.016389562 | 0.0013782 | beta(139.09, 8.35E+03) |
| Female | 50 to 54 | stroke | 0.023088777 | 0.0019860 | beta(132.01, 5.59E+03) |
| Female | 55 to 59 | stroke | 0.03132448 | 0.0023972 | beta(165.37, 5.11E+03) |
| Female | 60 to 64 | stroke | 0.040211155 | 0.0030748 | beta(164.11, 3.92E+03) |
| Female | 65 to 69 | stroke | 0.047674799 | 0.0037679 | beta(152.41, 3.04E+03) |
| Female | 70 to 74 | stroke | 0.052729227 | 0.0048780 | beta(110.63, 1.99E+03) |
| Female | 75 to 79 | stroke | 0.05503839 | 0.0054219 | beta(97.32, 1.67E+03) |
| Female | 80 to 84 | stroke | 0.052425136 | 0.0057831 | beta(77.82, 1.41E+03) |
| Female | 85 to 89 | stroke | 0.040742651 | 0.0049244 | beta(65.62, 1.55E+03) |

# Appendix E: Disease Cost Estimation

Disease costs for India included healthcare expenditures for both inpatient and outpatient care including physician fees, medications, diagnostic test, hospital bed charges and ambulatory services. These estimates are from the 2017/2018 National Sample Survey (NSS) and include data regarding hospitalizations in the year prior to the interview date and for outpatient visits in the fifteen days prior to the response.^13^ The values for outpatient visits were scaled by a factor of 24.33 to reflect the annual costs consistent with the approach taken in two studies by John.^19,20^

The costs for Pakistan were assumed to be equivalent to those for India due to the lack of country specific data. Disease costs as per a recent report on the economic cost of tobacco use in Bangladesh were incorporated within the Bangladesh model.^9^ The cost of treatment for oesophageal and pharyngeal cancer were assumed to be equivalent to oral cancer. Included within the cost estimate were both the cost of inpatient hospital care and outpatient out of pocket expenditures over a 12-month period. In the absence of data regarding public healthcare costs, private healthcare expenditure estimates were implemented.

**Appendix F: Stroke Costs - Incident versus Prevalent Case**

As the model is prevalence based, the estimated number of cases of stroke includes both incident cases within the current cycle and prevalent cases from previous cycles. The estimated costs are specific to incident cases, therefore to estimate the costs of stroke the proportion of prevalent cases that were incident within a given cycle and the costs for treating prevalent cases of stroke are required.

Based on data from the Global Burden of Disease Study, the average ratio of incident to prevalent cases of stroke in India is 10%, i.e. one in ten cases of stroke was incident in the last year. ^12^ We chose India to base this estimate on as it has the most data and therefore likely the least uncertainty in the estimate.

We were unable to locate data regarding the cost of the treatment of prevalent cases of stroke for India, Bangladesh or Pakistan. In the absence of this data we explored information from studies in other countries to inform the relative costs of prevalent versus incident cases of stroke. We conservatively estimated the relative annual cost of prevalent stroke at 10% of the first year cost of incident stroke. This is at the low end of the estimates from other counties but attempts to take into consideration the limited number of well-organized stroke services, particularly within the public sector in India, Bangladesh and Pakistan.^14^ A high level of uncertainty was incorporated for this parameter estimate within the probabilistic sensitivity analysis.

The following is a summary of the studies we explored which reported the costs of incident and prevalent stroke.

A recent UK study estimated the mean annual cost per person from an NHS perspective of £13,269 in the first year post-stroke and a mean cost of £2,215 per annum for subsequent years, approximately 17% of the first year costs.^15^

An Australian study published in 2014 reported an average direct cost of incident stroke in the first year of USD $19,992 for ischemic stroke and USD $11,706 for hemorrhagic stroke.^16^ The annual direct costs for years 3 to 5 post stroke were estimated at USD $3,682 for ischemic stroke and $4,051 for hemorrhagic stroke, 18% and 35% of the incident costs, respectively.

In 1996 Taylor and colleagues estimated the life time costs of stroke in the United States.^17^ The annual long term follow up costs of prevalent stroke ranged from approximately 15% to 30% of the costs of incident stroke.

In 2018, Xu and colleagues published an estimate of the patient-level health economic outcomes of stroke using data from the UK national stroke register.^18^ The 1 year healthcare costs per patient were reported at £13,452 and the average annual healthcare costs for years 2 to 5 post stroke were £1,128, approximately 8% of the year 1 costs.

# Appendix G: Cost Conversion

| Country | Equivalent to One United States Dollar in 2019 |
| --- | --- |
| India | INR 70.42 |
| Bangladesh | BDT 84.45 |
| Pakistan | PKR 150.04 |

# Appendix H: Disability weights

The morbidity impact of ST related diseases was calculated as the number of disability adjusted life years lost using disability weights from the 2016 Global Burden of Disease study.^10^ As the disability weights for cancer are stage specific and the disability weights for stroke are severity specific, we calculated the average disability associated with a prevalent case of the cancer and stroke by weighting the disability weights by the time spent in each state for cancer and by the proportion of the prevalent cases with each severity for stroke. Details of the calculations are provided below.

We calculated the average duration of disease from the Global Burden of Disease study reported prevalence and incidence rates of diseases by age and sex for each country using the formula:

Prevalence = (Incidence Rate) x (Average Duration of Disease)

Source: Relationship Among Prevalence, Incidence Rate, and Average Duration of Disease, available at: [http://sphweb.bumc.bu.edu/otlt/MPH-](http://sphweb.bumc.bu.edu/otlt/MPH-Modules/EP/EP713_DiseaseFrequency/EP713_DiseaseFrequency7.html)

[Modules/EP/EP713_DiseaseFrequency/EP713_DiseaseFrequency7.html](http://sphweb.bumc.bu.edu/otlt/MPH-Modules/EP/EP713_DiseaseFrequency/EP713_DiseaseFrequency7.html)

For the cancers, to estimate the time spent in each stage of the disease we referenced a study conducted by the Global Burden of Disease Cancer Collaboration and published in 2016 which provided estimates of the duration of three of the cancer stages (diagnosis/treatment, metastases and terminal) with the remainder of the disease duration assigned to the control phase. We used the same estimates for the three disease stages as the collaboration and assigned the remainder of the disease duration to "controlled", thereby producing weights for calculating the average disability of a prevalent case.^11^

For stroke, we weighted the severity specific stroke disability weights by the estimated the percentage of individuals with mild, moderate and severe disease which were sourced from three epidemiological studies conducted in India.^12^

Finally, we combined the stage/severity weights for each of the diseases with the disability weights from the GBD study to estimate the average disability for prevalent cases of the disease. For the cancers these are specific to age group and sex. This approach is consistent with the approach taken by other researchers.^13^

# Appendix I: Key Assumptions

The risk of death was assumed to decrease with increased time since quitting ST with the risk in former users returning to that of never users ten years after quitting. The proportional decrease in risk with time since quitting was assumed to be equivalent to the reduction in risk with time since quitting smoked tobacco. Smoked tobacco was used to inform these risks due to the lack of data regarding ST. Based on a study by Cao and Kenfield the relative risk of mortality for current smokers versus never smokers was 2.04, for former smokers who had quit less than 5 years ago, it is 1.37 and for former smokers who quit 5 to 10 years ago it is 1.10.^10^ This relative decrease in risk was applied to the relative risk of mortality in current smokeless tobacco users (1.27) to estimate the relative risk for former users who quit less than 5 years ago (1.10) and for former users who quit 5 to 10 years ago (1.03).^9^

ST was assumed to not impact mortality or disease prevalence for people aged below 35 years. This assumption is justified by the lack of data regarding differential mortality and morbidity in this age group and the low disease prevalence. It therefore is both conservative, and will have minimal impact.

The prevalence of former ST users is reported at low levels across all three countries and across all age groups. For model projections, it was assumed to remain at currently reported age and sex specific rates over the life course. The most appropriate data on which to forecast future former ST prevalence was assumed to be the currently available reported prevalences. This assumption is supported by the observation of consistently low reported prevalences for former users between the 2009/2010 Indian GATS survey and the 2016/2017 Indian GATS survey. In the absence of similar data for other counties, the same assumption was incorporated.

Although there is published evidence supporting an increased risk of other diseases associated with ST including cardiovascular disease, respiratory diseases and other cancers, the impact of ST was assessed through its association with only four diseases, specifically oral cancer, oesophageal cancer, pharyngeal cancer and stroke. The decision to focus on four diseases was conservative.

The evidence for the four diseases was strongest with studies specific to the population of interest

(South East Asia) and reflecting the majority of the burden.

# Appendix J: Lifetime ST attributable discounted costs per individual (US$)

Men (India) Women (India)


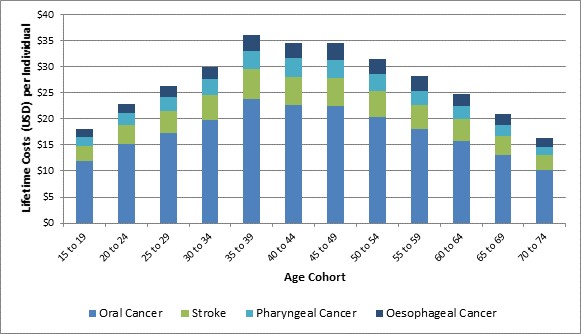

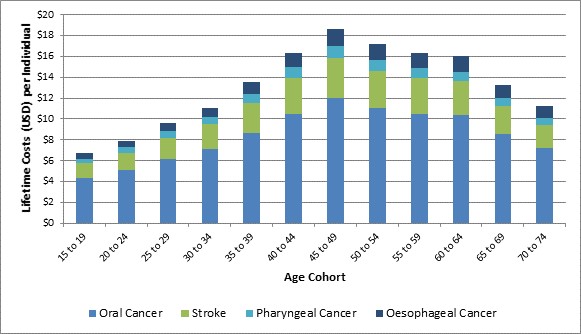


Men (Bangladesh) Women (Bangladesh)


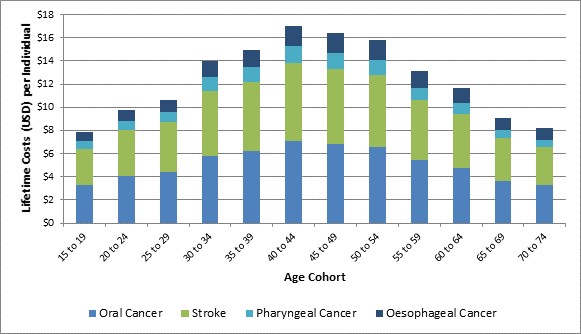

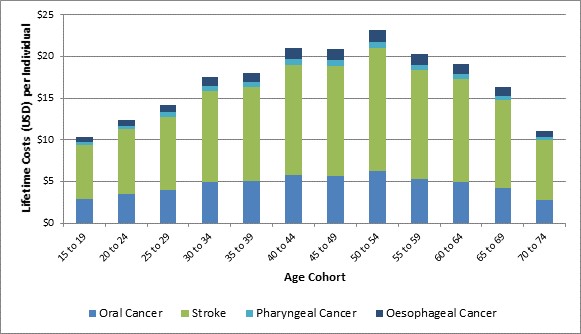


Men (Pakistan) Women (Pakistan)


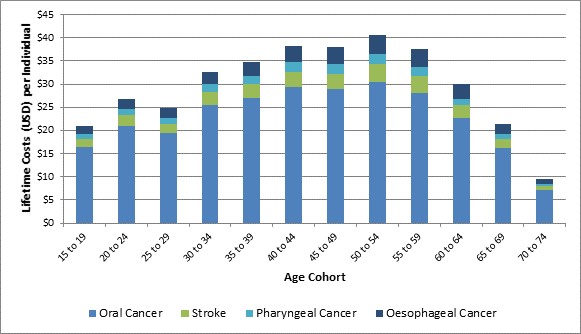

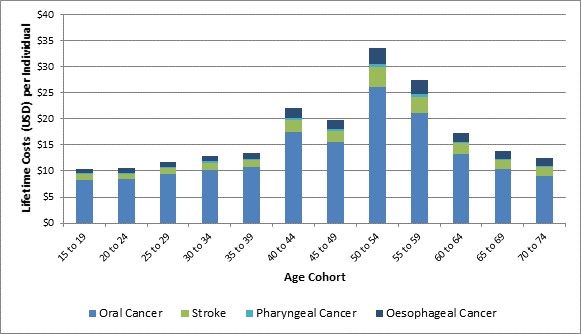


# Appendix K: DALYs (discounted) lost to smokeless tobacco

Men (India) Women (India)


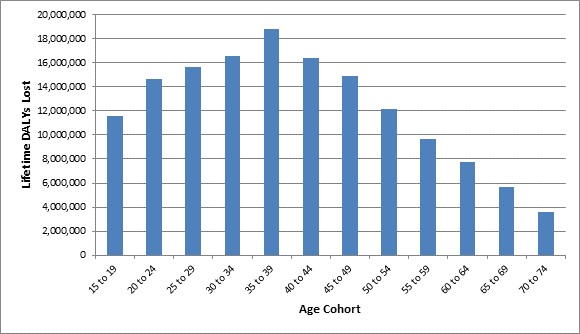

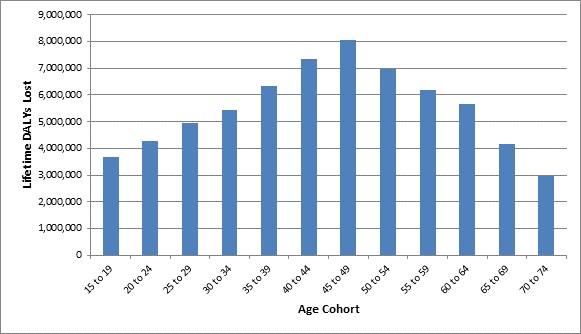


Men (Bangladesh) Women (Bangladesh)


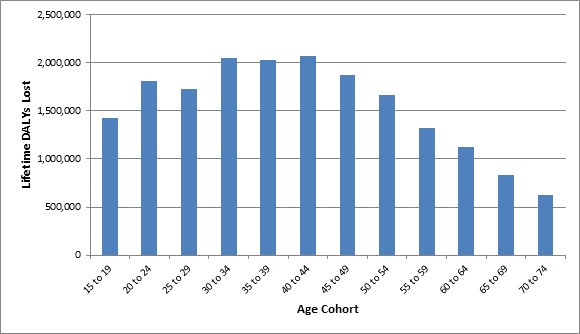

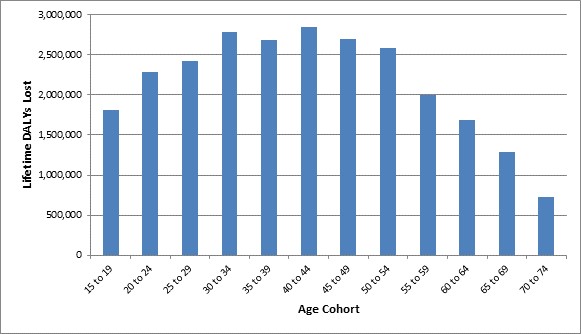


Men (Pakistan) Women (Pakistan)


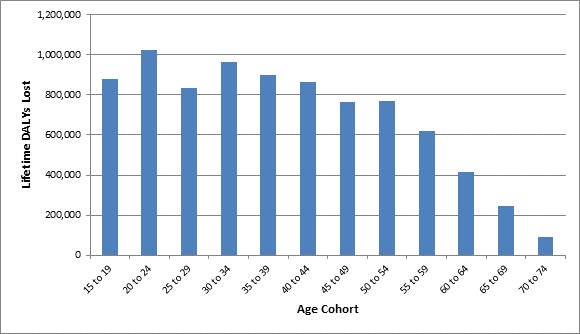

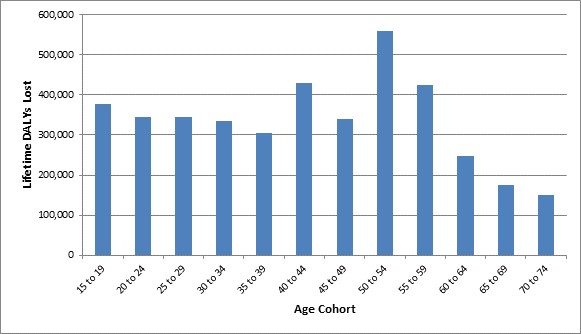


**Appendix L**: **DALYs (discounted) lost to smokeless tobacco per individual**

| Men (India)  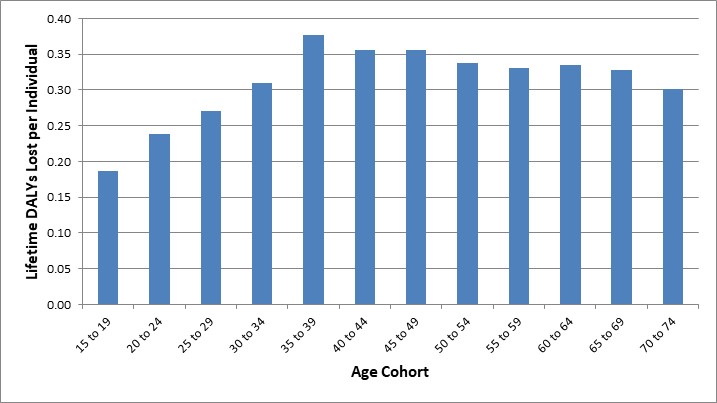 | Women (India)  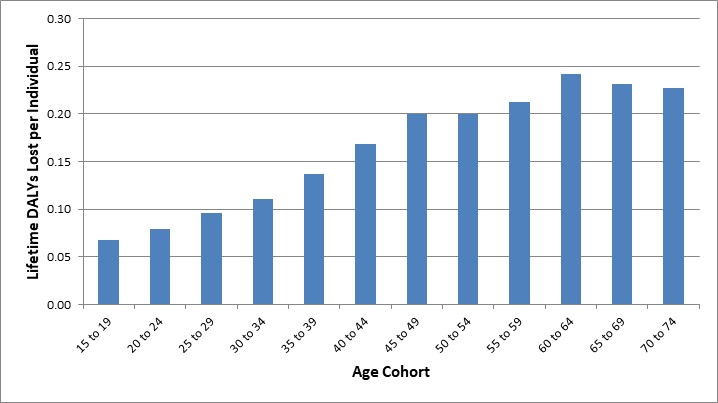 |
| --- | --- |
| Men (Bangladesh)  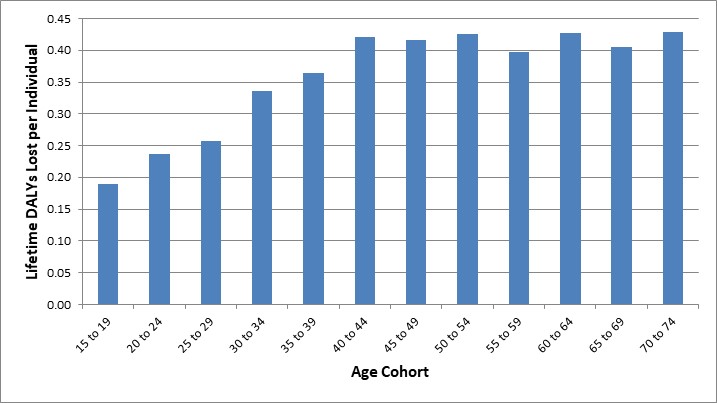 | Women (Bangladesh)  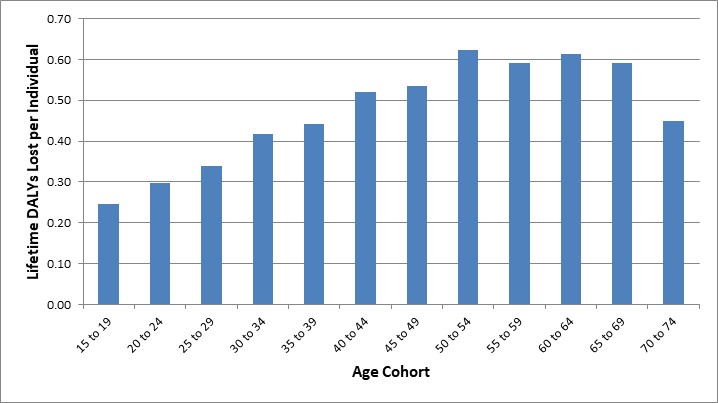 |
| Men (Pakistan)  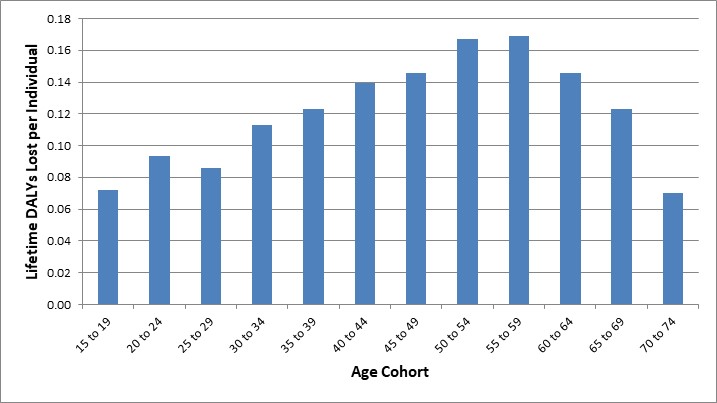 | Women (Pakistan)  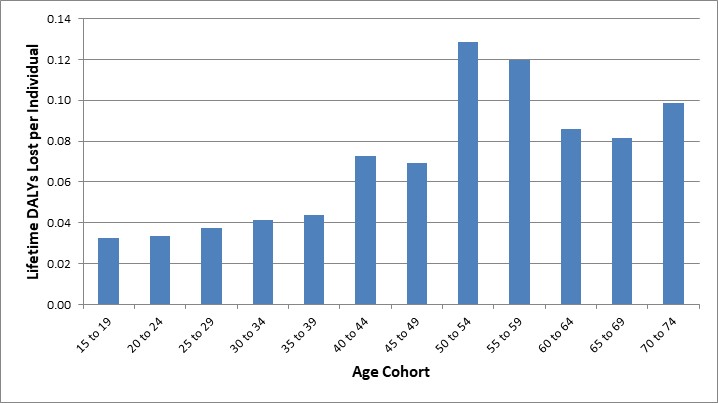 |

**Appendix M: Disability Adjusted Life Years Lost and Costs (2019 US dollars) Attributable**

# To Smokeless Tobacco (discounted)

| **Sex** | **Age** | **India** | **Pakistan** | **Bangladesh** |
| --- | --- | --- | --- | --- |
| **Disability adjusted life years (95% credibility interval)** | | | |  |
| **Female** | 15 to 19 | 3,681,387 (1,337,008 to 6,199,413) | 378,406 (120,515 to 799,893) | 1,806,008 (705,781 to 2,916,456) |
| **Female** | 20 to 24 | 4,280,893 (1,570,143 to 7,204,223) | 345,774 (114,895 to 729,341) | 2,280,266 (892,496 to 3,677,427) |
| **Female** | 25 to 29 | 4,928,774 (1,794,400 to 8,273,052) | 344,937 (111,652 to 743,844) | 2,416,078 (941,214 to 3,910,035) |
| **Female** | 30 to 34 | 5,429,876 (1,943,782 to 9,088,585) | 335,287 (110,413 to 735,866) | 2,789,608 (1,093,975  to 4,510,309) |
| **Female** | 35 to 39 | 6,349,167 (2,314,028 to 10,557,250) | 305,056 (90,676 to 702,396) | 2,679,356 (1,036,085  to 4,327,113) |
| **Female** | 40 to 44 | 7,343,127 (2,678,282 to 12,174,721) | 428,434 (151,312 to 866,225) | 2,845,960 (1,105,446  to 4,609,917) |
| **Female** | 45 to 49 | 8,060,291 (2,921,713 to 13,326,264) | 339,685 (113,504 to 726,532) | 2,699,249 (1,036,837  to 4,392,272) |
| **Female** | 50 to 54 | 6,985,614 (2,550,012 to 11,642,192) | 558,607 (194,875 to 1,121,515) | 2,587,635 (995,459  to 4,174,182) |
| **Female** | 55 to 59 | 6,169,111 (2,249,146 to 10,230,694) | 423,352 (144,335 to 803,886) | 1,992,876 (747,036 to 3,249,086) |

| **Female** | 60 to 64 | 5,646,086 (2,042,458 to 9,360,446) | 248,593 (79,712 to 514,765) | 1,680,567 (640,071 to 2,726,350) |
| --- | --- | --- | --- | --- |
| **Female** | 65 to 69 | 4,170,546 (1,502,973 to 6,933,244) | 175,467 (53,519 to 372,339) | 1,287,908 (492,472 to 2,088,613) |
| **Female** | 70 to 74 | 2,972,026 (1,088,590 to 4,939,335) | 148,965 (22,050 to 386,538) | 725,151 (267,611 to 1,199,410) |
| **Male** | 15 to 19 | 11,598,725 (4,107,411 to 18,986,654) | 880,418 (311,653 to 1,624,403) | 1,431,084 (529,225 to 2,371,169) |
| **Male** | 20 to 24 | 14,614,104 (5,157,893 to 24,115,758) | 1,021,989 (368,057 to 1,865,580) | 1,810,544 (664,412 to 3,011,939) |
| **Male** | 25 to 29 | 15,662,797 (5,524,405 to 25,758,207) | 833,416 (307,114 to 1,528,304) | 1,726,302 (628,558 to 2,872,650) |
| **Male** | 30 to 34 | 16,602,769 (5,972,203 to 27,268,877) | 916,844 (358,660 to 1,694,987) | 2,047,630 (755,634 to 3,402,793) |
| **Male** | 35 to 39 | 18,821,542 (6,686,806 to 30,939,896) | 897,289 (341,705 to 1,583,222) | 2,030,772 (739,125 to 3,340,344) |
| **Male** | 40 to 44 | 16,414,254 (5,837,193 to 27,120,423) | 866,424 (333,228 to 1,518,022) | 2,067,976 (759,553 to 3,415,503) |
| **Male** | 45 to 49 | 14,924,803 (5,270,893 to 24,550,875) | 763,082 (286,229 to 1,346,927) | 1,868,931 (689,474 to 3,110,085) |
| **Male** | 50 to 54 | 12,118,526 (4,267,649 to 20,028,341) | 766,992 (261,715 to 1,486,316) | 1,664,118 (604,509 to 2,756,304) |

| **Male** | 55 to 59 | 9,696,884 (3,476,546 to 16,008,234) | 621,395 (201,311 to 1,208,078) | 1,319,567 (478,953 to 2,181,167) |
| --- | --- | --- | --- | --- |
| **Male** | 60 to 64 | 7,740,056 (2,683,335 to 12,731,639) | 415,611 (111,196 to 927,896) | 1,121,611 (405,667 to 1,887,138) |
| **Male** | 65 to 69 | 5,689,453 (1,956,216 to 9,284,774) | 244,420 (63,296 to 535,784) | 830,137 (298,069 to 1,390,532) |
| **Male** | 70 to 74 | 3,618,464 (1,255,829 to 5,972,752) | 90,953 (20,892 to 206,639) | 628,808 (226,814 to 1,041,254) |
| **Incremental cost in thousands of US dollars (95% confidence interval)** | | | | |
| **Female** | 15 to 19 | $367,318 ($262,481 to $494,294) | $120,950 ($54,048 to $224,697) | $75,370 ($32,075 to $129,034) |
| **Female** | 20 to 24 | $427,267 ($306,845 to $574,184) | $109,269 ($51,086 to $199,053) | $95,332 ($40,512 to $163,019) |
| **Female** | 25 to 29 | $492,468 ($355,411 to $655,264) | $108,420 ($47,785 to $202,910) | $100,529 ($42,363 to $172,566) |
| **Female** | 30 to 34 | $542,370 ($394,934 to $724,188) | $104,723 ($48,159 to $196,783) | $116,579 ($49,562 to $199,154) |
| **Female** | 35 to 39 | $628,148 ($454,981 to $832,234) | $93,426 ($38,050 to $183,242) | $109,558 ($46,262 to $188,299) |
| **Female** | 40 to 44 | $711,989 ($514,242 to $937,920) | $129,762 ($68,422 to $224,439) | $114,762 ($48,304 to $199,240) |
| **Female** | 45 to 49 | $753,859 ($553,333 to $1,003,887) | $97,466 ($46,387 to $182,503) | $105,365 ($43,342 to $183,029) |

| **Female** | 50 to 54 | $602,969 ($438,147 to $803,187) | $146,022 ($72,565 to $257,430) | $96,243 ($38,918 to $168,425) |
| --- | --- | --- | --- | --- |
| **Female** | 55 to 59 | $473,870 ($337,820 to $640,303) | $96,840 ($48,475 to $167,106) | $68,204 ($26,149 to $120,766) |
| **Female** | 60 to 64 | $374,361 ($266,686 to $505,115) | $50,130 ($21,650 to $96,321) | $52,185 ($19,552 to $92,507) |
| **Female** | 65 to 69 | $239,075 ($166,486 to $325,901) | $29,899 ($12,495 to $57,362) | $35,443 ($12,733 to $63,373) |
| **Female** | 70 to 74 | $147,447 ($100,927 to $203,346) | $18,963 ($3,304 to $46,422) | $17,786 ($5,918 to $33,079) |
| **Male** | 15 to 19 | $1,122,498 ($856,526 to $1,443,839) | $254,684 ($147,200 to $400,004) | $59,343 ($34,831 to $90,437) |
| **Male** | 20 to 24 | $1,405,294 ($1,078,758 to $1,790,083) | $293,869 ($177,424 to $456,986) | $74,732 ($44,138 to $113,560) |
| **Male** | 25 to 29 | $1,515,264 ($1,170,057 to $1,934,334) | $241,287 ($147,827 to $369,774) | $71,462 ($42,234 to $109,625) |
| **Male** | 30 to 34 | $1,606,495 ($1,242,575 to $2,035,346) | $278,490 ($183,393 to $404,622) | $84,919 ($50,732 to $128,432) |
| **Male** | 35 to 39 | $1,803,343 ($1,398,912 to $2,287,491) | $253,916 ($165,814 to $366,893) | $83,276 ($49,252 to $126,979) |
| **Male** | 40 to 44 | $1,597,231 ($1,231,931 to $2,037,151) | $238,023 ($154,491 to $352,694) | $83,934 ($50,518 to $127,064) |
| **Male** | 45 to 49 | $1,449,171 ($1,117,987 to $1,856,432) | $199,730 ($128,569 to $295,492) | $73,934 ($43,965 to $112,296) |
| **Male** | 50 to 54 | $1,134,118 ($858,135 to $1,469,285) | $186,579 ($96,240 to $321,185) | $61,864 ($36,356 to $94,667) |
| **Male** | 55 to 59 | $825,817 ($612,466 to $1,068,337) | $138,271 ($71,453 to $239,473) | $43,570 ($25,809 to $66,894) |
| **Male** | 60 to 64 | $574,957 ($421,829 to $753,599) | $85,488 ($31,934 to $169,240) | $30,649 ($17,364 to $48,029) |
| **Male** | 65 to 69 | $361,329 ($262,079 to $483,220) | $42,630 ($15,485 to $84,109) | $18,566 ($10,107 to $29,959) |
| **Male** | 70 to 74 | $197,471 ($138,172 to $270,907) | $12,218 ($3,866 to $25,980) | $12,016 ($6,404 to $19,148) |

***References*** [to the citations in the Supplementary Materials, Appendices]

1. United States Census Bureau. International Programs - Information Gateway - International Database. Published 2020. Accessed December 8, 2020. https://www.census.gov/data-tools/demo/idb/informationGateway.php

2. Tata Institute of Social Sciences, Ministry of Health and Family Welfare Government of India. *Global Adult Tobacco Survey GATS 2 India 2016-2017*.; 2017. https://mohfw.gov.in/sites/default/files/GlobaltobacoJune2018.pdf

3. World Health Organization Country Office for Bangladesh, Ministry of Health and Family Welfare. *Global Adult Tobacco Survey: Bangladesh Report 2009*.; 2009. https://www.who.int/tobacco/surveillance/global_adult_tobacco_survey_bangladesh_report_2009.pdf?ua=1

4. Pakistan Health Research Council, World Health Organization. Global Adult Tobacco Survey: Pakistan 2014. Published online 2016. https://www.who.int/tobacco/surveillance/survey/gats/pak-report.pdf?ua=1

5. Ministry of Health and Family Welfare Government of India. *Global Adult Tobacco Survey (GATS) India 2009-2010*.; 2010. doi:10.1002/aehe.3640230702

6. Hughes JR, Peters EN, Naud S. Relapse to smoking after 1 year of abstinence: A meta-analysis. *Addict Behav*. 2008;33(12):1516-1520. doi:10.1016/j.addbeh.2008.05.012

7. Office of the Registrar General & Census Commissioner. SRS Based Abridged Life Tables 2013-2017. Published 2019. Accessed October 10, 2019. http://www.censusindia.gov.in/vital_statistics/Appendix_SRS_Based_Life_Table.html

8. United Nations / DESA / Population Division. World Population Prospects 2019. Published 2019. Accessed October 10, 2019. https://population.un.org/wpp/Download/Standard/Mortality/

9. Sinha DN, Suliankatchi RA, Gupta PC, et al. Global burden of all-cause and cause-specific mortality due to smokeless tobacco use: Systematic review and meta-analysis. *Tob Control*. 2018;27(1):35-42. doi:10.1136/tobaccocontrol-2016-053302

10. Cao Y, Kenfield S, Song Y, et al. Cigarette smoking cessation and total and cause-specific mortality: A 22-year follow-up study among US male physicians. *Arch Intern Med*. 2011;171(21):1956-1959. doi:10.1001/jama.299.17.2037.Smoking

11. Flack S, Taylor M, Consultant S, Trueman P. Cost-Effectiveness of Interventions for Smoking Cessation Final Report. 2007;(4144762). https://www.nice.org.uk/guidance/ph10/resources/smoking-cessation-services-economics-modelling-report-2

12. Institute for Health Metrics and Evaluation. Global Health Data Exchange, Global Burden of Disease Results Tool. Published 2020. Accessed November 9, 2020. http://ghdx.healthdata.org/gbd-results-tool

13. Ministry of Statistics and Programme Implementation. *National Sample Survey (NSS)*; 2020

14. Kaur P, Kwatra G, Kaur R, Pandian JD. Cost of stroke in low and middle income countries: A systematic review. *Int J Stroke*. 2014;9(6):678-682. doi:10.1111/ijs.12322

15. Patel A, Berdunov V, Quayyum Z, King D, Knapp M, Wittenberg R. Estimated societal costs of stroke in the UK based on a discrete event simulation. *Age Ageing*. 2020;49(2):270-276. doi:10.1093/ageing/afz162

16. Gloede T, Halbach S, Thrift A, Dewey H, Pfaff H, Cadilhac D. Long-term costs of stroke using 10-year longitudinal data from the north east melbourne stroke incidence study. *Stroke*. 2014;45(11):3389-3394.

17. Taylor TN, Davis PH, Torner JC, Holmes J, Meyer JW, Jacobson MF. Lifetime cost of stroke in the United States. *Stroke*. 1996;27(9):1459-1466. doi:10.1161/01.STR.27.9.1459

18. Xu XM, Vestesson E, Paley L, et al. The economic burden of stroke care in England, Wales and Northern Ireland: Using a national stroke register to estimate and report patient-level health economic outcomes in stroke. *Eur Stroke J*. 2018;3(1):82-91. doi:10.1177/2396987317746516

19. John RM, Sung HY, Max W. Economic cost of tobacco use in India, 2004. *Tob Control*. 2009;18(2):138-143. doi:10.1136/tc.2008.027466

20. John RM. Economic costs of diseases and deaths attributable to bidi smoking in India, 2017. *Tob Control*. 2019;28(5):513-518. doi:10.1136/tobaccocontrol-2018-054493
